# Exploring the mechanism of *Alisma orientale* for the treatment of pregnancy induced hypertension and potential hepato-nephrotoxicity by using network pharmacology, network toxicology, molecular docking and molecular dynamics simulation

**DOI:** 10.3389/fphar.2022.1027112

**Published:** 2022-11-15

**Authors:** Yilin Liao, Yiling Ding, Ling Yu, Cheng Xiang, Mengyuan Yang

**Affiliations:** ^1^ Department of Obstetrics and Gynecology, The Second Xiangya Hospital of Central South University, Changsha, Hunan, China; ^2^ Department of Orthopedics, The Second Xiangya Hospital of Central South University, Changsha, China

**Keywords:** pregnancy induced hypertension, *Alisma orientale*, network pharmacology, network toxicology, molecular docking

## Abstract

**Background:** Pregnancy-induced Hypertension (PIH) is a disease that causes serious maternal and fetal morbidity and mortality. Alisma Orientale (AO) has a long history of use as traditional Chinese medicine therapy for PIH. This study explores its potential mechanism and biosafety based on network pharmacology, network toxicology, molecular docking and molecular dynamics simulation.

**Methods:** Compounds of AO were screened in TCMSP, TCM-ID, TCM@Taiwan, BATMAN, TOXNET and CTD database; PharmMapper and SwissTargetPrediction, GeneCards, DisGeNET and OMIM databases were used to predict the targets of AO anti-PIH. The protein-protein interaction analysis and the KEGG/GO enrichment analysis were applied by STRING and Metascape databases, respectively. Then, we constructed the “herb-compound-target-pathway-disease” map in Cytoscape software to show the core regulatory network. Finally, molecular docking and molecular dynamics simulation were applied to analyze binding affinity and reliability. The same procedure was conducted for network toxicology to illustrate the mechanisms of AO hepatotoxicity and nephrotoxicity.

**Results:** 29 compounds with 78 potential targets associated with the therapeutic effect of AO on PIH, 10 compounds with 117 and 111 targets associated with AO induced hepatotoxicity and nephrotoxicity were obtained, respectively. The PPI network analysis showed that core therapeutic targets were IGF, MAPK1, AKT1 and EGFR, while PPARG and TNF were toxicity-related targets. Besides, GO/KEGG enrichment analysis showed that AO might modulate the PI3K-AKT and MAPK pathways in treating PIH and mainly interfere with the lipid and atherosclerosis pathways to induce liver and kidney injury. The “herb-compound-target-pathway-disease” network showed that triterpenoids were the main therapeutic compounds, such as Alisol B 23-Acetate and Alisol C, while emodin was the main toxic compounds. The results of molecular docking and molecular dynamics simulation also showed good binding affinity between core compounds and targets.

**Conclusion:** This research illustrated the mechanism underlying the therapeutic effects of AO against PIH and AO induced hepato-nephrotoxicity. However, further experimental verification is warranted for optimal use of AO during clinical practice.

## Introduction

Pregnancy-induced Hypertension (PIH) encompasses a series of serious pregnancy complications in the second trimester (after 20 weeks of gestation), including pre-eclampsia, eclampsia, chronic hypertension with pre-eclampsia and chronic hypertension with pregnancy (Chen et al., 2019). Little is currently known about the exact etiology, although it has been established that genetic, immunological and oxidative stress are risk factors (Magalhães et al., 2020). The pathological features include endothelial cell dysfunction, multiple cytokine stimulation and activation of the coagulation system with vasospasm and increased vascular reactivity, which lead to inadequate spiral arterioles and decreased invasion of trophoblasts into the maternal decidua of the uterus, leading to a series of clinical symptoms, such as generalized edema, vomiting, blurred vision, proteinuria and even disseminated intravascular coagulation (DIC) (Chaiworapongsa et al., 2014). Furthermore, it causes fetal growth retardation, premature birth and perinatal stillborn fetus (Al Khalaf et al., 2022). Current evidence suggests that PIH is a significant threat to more than 70,000 gravidas and half a million fetuses worldwide yearly, with a high maternal mortality rate ranging from 10 to 16% (Bruno et al., 2022).

The complexity of the interactions also limits the efficacy of treatment. Currently available management consists of symptomatic treatment, including spasmolytics (magnesium sulfate), anticoagulants (aspirin), antihypertensive drugs, and termination of pregnancy as a last resort (Zhang et al., 2022a). Nevertheless, the clinical curative efficacy is highly heterogeneous and has been reported to cause maternal and fetal adverse effects (Ni et al., 2022).

Traditional Chinese medicine (TCM) therapy is an ancient practice used for more than 2000 years in China (Tian et al., 2021). The application of TCM therapy in modern society provides a novel approach for complementary and alternative medicine (CAM) treatment and is recognized by more and more people worldwide (Chu et al., 2022). Zhong-jing Zhang originally described PIH in “Synopsis in the Golden Chamber” as “nausea in pregnancy” “eclampsia” and “edema during pregnancy” (Xue et al., 2016). According to the syndrome differentiation theory, PIH is caused by Yin deficiency and Yang excess, deficiency of both spleen and kidney and blood stasis (Yeh et al., 2009). Over the years, many therapeutic methods and prescriptions of TCM have been used to treat PIH, and countless patients have benefited from it (Perry et al., 2018).

Alisma Orientale (AO), also called Ze Xie in Chinese, is a high-efficacy and low-toxicity herb medicine primarily used in Southeast Asian countries. Its efficacy is mainly mediated by removing dampness and promoting water metabolism (Wang et al., 2022), accounting for its efficacy in treating oliguria, edema and hypertension. “Women’s prescription” also recorded that AO could relieve systemic edema of gravida. In recent years, pharmacological research has demonstrated that AO compounds exhibit diuretic, anti-atherosclerotic and immunomodulatory activities, which all suitable for the treatment of PIH (Loh et al., 2017). Chen et al. reported that the natural compound of AO, alisol B 23-acetate, could inhibit Ang II-induced RAS/Wnt/β-catenin axis and attenuate podocyte injury (Chen et al., 2017). Moreover, it could inhibit collagen I, vimentin and α-smooth muscle actin at the mRNA and protein levels in rats (Chen et al., 2020). Notwithstanding that hundreds of active compounds have been isolated from AO, the specific compounds and correlative mechanisms have been largely understudied. Therefore, a novel method is urgently needed to reveal the complex treatment network and identify compounds underlying the therapeutic effect of AO against PIH. Besides, toxicology studies on AO have revealed that chronic administration may induce mild nephrotoxicity and hepatotoxicity (Yuen et al., 2006), emphasizing the need to verify the toxicity and safety profile of AO compounds and their molecular mechanisms.

The concept of network pharmacology was first officially reported by the British Pharmacologist Hopkins in 2007 (Hopkins, 2008), while network toxicology was originally proposed by Academician Liu of Chinese in 2011 (Li et al., 2019a). Both methods were based on the theories of “multi-target” and “multi-pathway” between the drug and disease, which are consistent with the characteristics of TCM therapy (Li et al., 2022).

**FIGURE 1:**
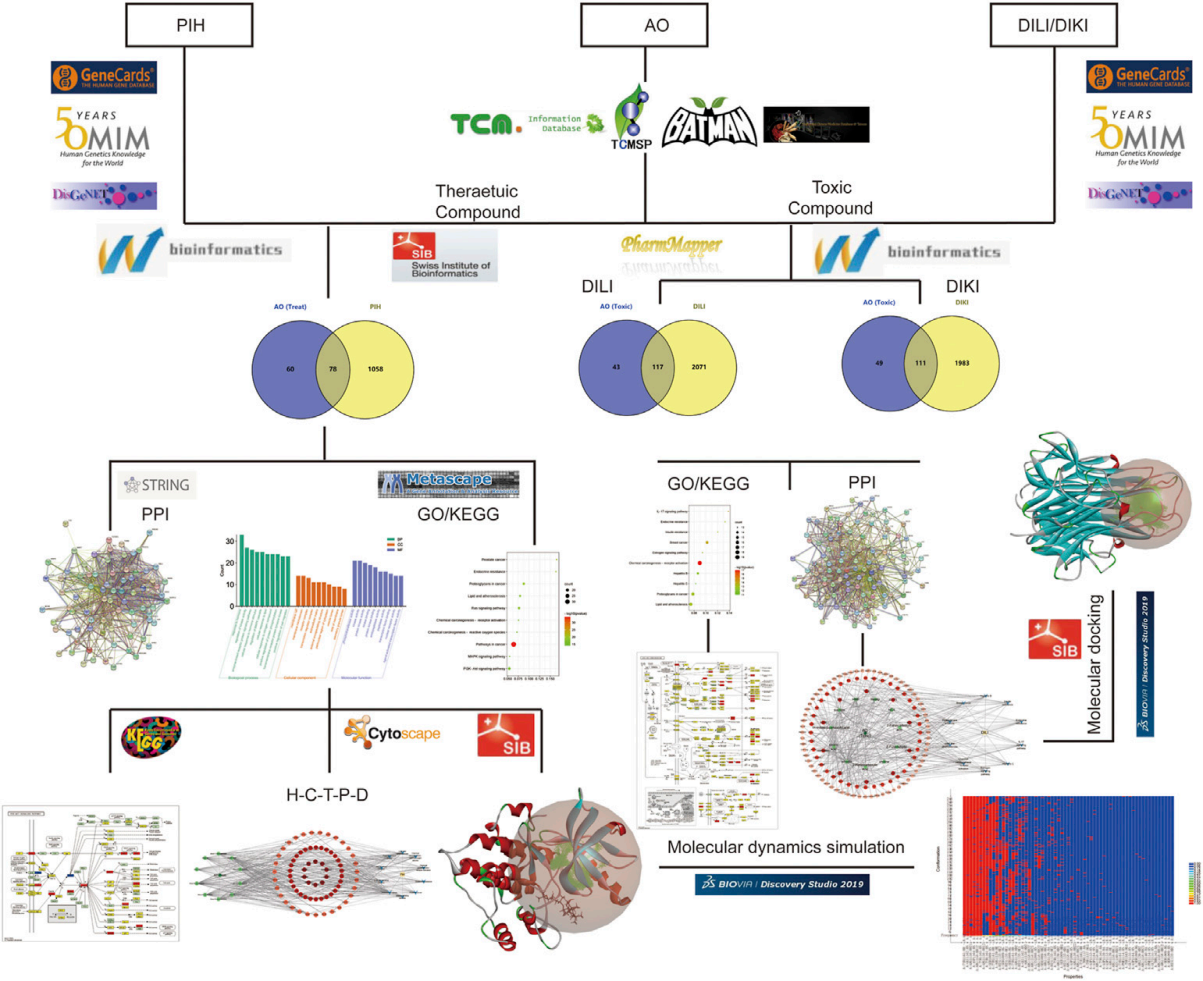
The flow chart of this study to explore the potential molecular mechanism of AO in the treatment of PIH and toxic compounds induced hepato-nephrotoxicity.

In this study, we applied network pharmacology to screen the main therapeutic compounds of AO and predicted the potential mechanism. In addition, we analyzed the potential hepato-nephrotoxicity mechanism of AO compounds by network toxicology during the treatment of PIH. Finally, molecular docking and molecular dynamics simulation analyses were used to estimate the binding stability. Overall, this study improves our current understanding of the potential pharmacodynamic and toxicity mechanism of AO in PIH patients and explores how to enhance efficacy and reduce toxicity. The detailed research content of this study is presented in the flow chart (Figure 1).

## Methods

### Obtaining therapeutic and toxicity-related compounds

The Traditional Chinese Medicine Systems Pharmacology Database and Analysis Platform (TCMSP http://lsp.nwu.edu.cn/tcmsp.php) database (Ru et al., 2014), Traditional Chinese Medicine Information Database (Kang et al., 2013) (TCM-ID http://bidd.group), BATMAN-TCM (Liu et al., 2016) (a bioinformatics analysis Tool for molecular mechanism of traditional Chinese medicine http://bionet.ncpsb.org.cn/) and TCM@Taiwa (Chen, 2011) (http://tcm.cmu.edu.tw/zh-tw/) database were used to acquire detailed information on identified AO compounds. Oral bioavailability (OB) refers to the dosage of drugs that enter the blood circulation through oral absorption and act on local tissues and organs to produce corresponding pharmacological effects. Drug-likeness (DL) is defined as the similarity of the chemical structure of a drug compared with known drugs. Both characteristics are important to assess the potential medicinal value of compounds (Dai et al., 2022). We selected the biologically active compounds of AO in the TCMSP database using the screening criteria: OB ≥ 30 and DL ≥ 0.18 (Gao et al., 2022). Similarly, compounds from other database sources were screened in SwissADME (Daina et al., 2017) (http://www.swissadme.ch) database according to their pharmacokinetic parameters. The toxicity compound of AO was further searched in Toxicology data network (TOXNET, http://toxnet.nlm.nih.gov/index.html) database (Fowler and Schnall, 2014) and Comparative Toxicogenomics Database (CTD, https://toxnet.nlm.nih.gov/newtoxnet/ctd.htm) to analyze the biosafety profile of AO in the treatment of PIH (Stanic et al., 2021).

### Potential targets related to AO compounds, PIH, liver and kidney injury

The structural formulas of therapeutic and toxic compounds obtained in the previous step were input into the PharmMapper (Liu et al., 2010) (http://www.lilab-ecust.cn/pharmmapper/) and SwissTargetPrediction database (Daina et al., 2019) (http://www.swisstargetprediction.ch/) to predict the possible targets based on the spatial conformation. The uniport ID of the target was converted to the standardized gene name by Uniport (Holzhüter and Geertsma, 2022) (https://www.uniprot.org/) database. MeSH database (https://www.ncbi.nlm.nih.gov/mesh) was utilized to verify the standard name of the disease as “Pregnancy-induced Hypertension” “drug-induced liver injury” (DILI) and “drug-induced kidney injury” (DIKI) (Zouhal et al., 2021). Then, GeneCards database (Barshir et al., 2021) (https://www.genecards.org/), DisGeNET database (Piñero et al., 2020) (https://www.disgenet.org) and Online Mendelian Inheritance in Man (Li et al., 2012) (OMIM, https://omim.org) database were retrieved to obtain potential targets using the keywords “Pregnancy-induced Hypertension” “drug-induced liver injury” and “drug-induced kidney injury”. After merging and removing duplicate targets, the intersected targets in the Venn plot were defined as potential targets of OA compounds during PIH treatment and toxic compounds associated with hepato-nephrotoxicity.

### Constructing PPI network to screen core target

A protein-protein interaction (PPI) network was generated to identify interacting proteins. STRING (Szklarczyk et al., 2021) (http://string-db.org, Version 11.5) database was used to construct a PPI network of the anti-PIH effect of AO, with species limited to “*Homo sapiens*” and interaction score >0.4. The Cytoscape plug-ins Cytohubba and MCODE (molecular complex detection) were used to analyze the topological parameters and select the core targets of AO anti-HIP with high accuracy and exhibit the complex relationship between disease and herb (Ye et al., 2022). The same steps were used to construct the PPI network of network toxicology.

### GO and KEGG pathway enrichment analysis

Functional enrichment analysis was conducted to better understand the functions of the screened genes. Gene Ontology (GO) enables the analysis of gene function based on the cellular compound (CC), molecular function (MF) and biological process (BP). Kyoto Encyclopedia of Genes and Genomes (KEGG) enables an understanding of the biological pathways associated with genes. Both were used to illustrate the core pathway and mechanism of AO anti-HIP. Targets were inputted in the Metascape database (Zhou et al., 2019) (http://www.metascape.org/), and the cut-off *p*-value, min overlap and enrichment value were set to 0.01, 3 and 1.5, respectively. And *q*-value < 0.05 (Benjamini–Hochberg method) was used to remove the false positive enrichment results (Zou et al., 2016). The R package available on the bioinformatics website (http://www.bioinformatics.com.cn/) was used to visualize the enriched results as bar and bubble plots. Then, the KEGG (Kanehisa and Sato, 2020) (http://www.kegg.jp.org/) database was used to map and color the detailed target messages in the most significantly enriched pathway. Finally, the complex regulatory network of AO in the treatment of PIH was shown by the “herb-compound-target-pathway-disease” network, incorporating the elements of AO, therapeutic compound, potential target, and top 10 enriched pathway, and visualized by Cytoscape software (v.3.9.1, https://cytoscape.org/). The same steps were used to analyze the core pathways of toxic AO compounds associated with hepato-nephrotoxicity.

### Molecular dock verified the binding affinity

The Swiss dock platform (Grosdidier et al., 2011) (http://www.swissdock.ch/) is an online molecular docking (MD) tool that enables the calculation of the binding affinity of each binding site between small molecule ligand and receptor protein. The X-ray diffraction of the protein crystal structure of core targets was downloaded from the Protein Data Bank (PDB) database (www.rcsb.org) (Nakamura et al., 2022). The results were ranked by the binding affinity score, and the lowest binding affinity score value corresponded to the best binding site. The binding affinity < -7 kcal/mol indicates a strong combination possibility, which was visualized in Discovery Studio 2019 software (https://www.3ds.com/) (Sultana et al., 2022).

### Molecular dynamics simulation verified the binding stability

Molecular Dynamics simulations (MDS) were further conducted to investigate the stability of small molecule ligands in proteins by the “Standard Dynamics Cascade” unit of Discovery Studio2019 software for ligand-protein complexes with the lowest binding affinity after molecular docking (Hu et al., 2022). The ligand-protein complex was placed in a solvent chamber, filled with water molecules and stabilized the electrically neutral system with Cl− and Na+. After balancing the system by the NPT ensemble (fixed the pressure, temperature and number of particles), the simulation time value was set to 500 ps and heating, equilibrium and production phases were conducted. Finally, the result was derived by trajectories analyzed with Root Mean Square Deviation (RMSD), Root Mean Square Fluctuation (RMSF) and hydrogen bond properties.

## Result

### Target prediction of AO compounds, PIH, liver and kidney injury

**FIGURE 2:**
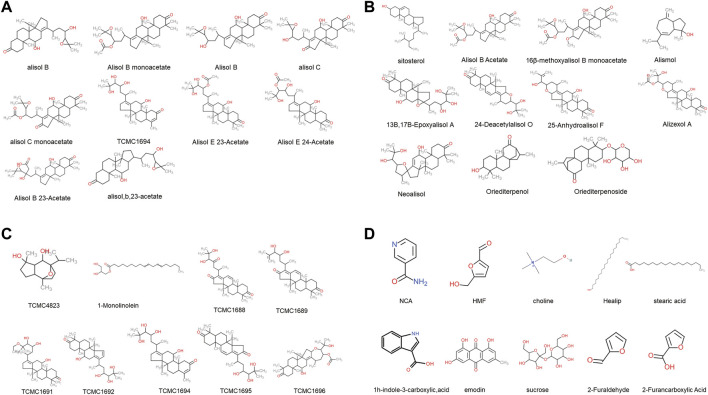
The chemical structure of 39 AO bioactive compounds obtained from TCMSP, TCM-ID, TCM@Taiwan and BATMAN-TCM database **(A)**, The chemical structure of top 10 therapeutic compounds **(B)** and **(C)**, The chemical structure of remaining 19 therapeutic compounds **(D)**, The chemical structure of 10 toxic compounds.

**FIGURE 3:**
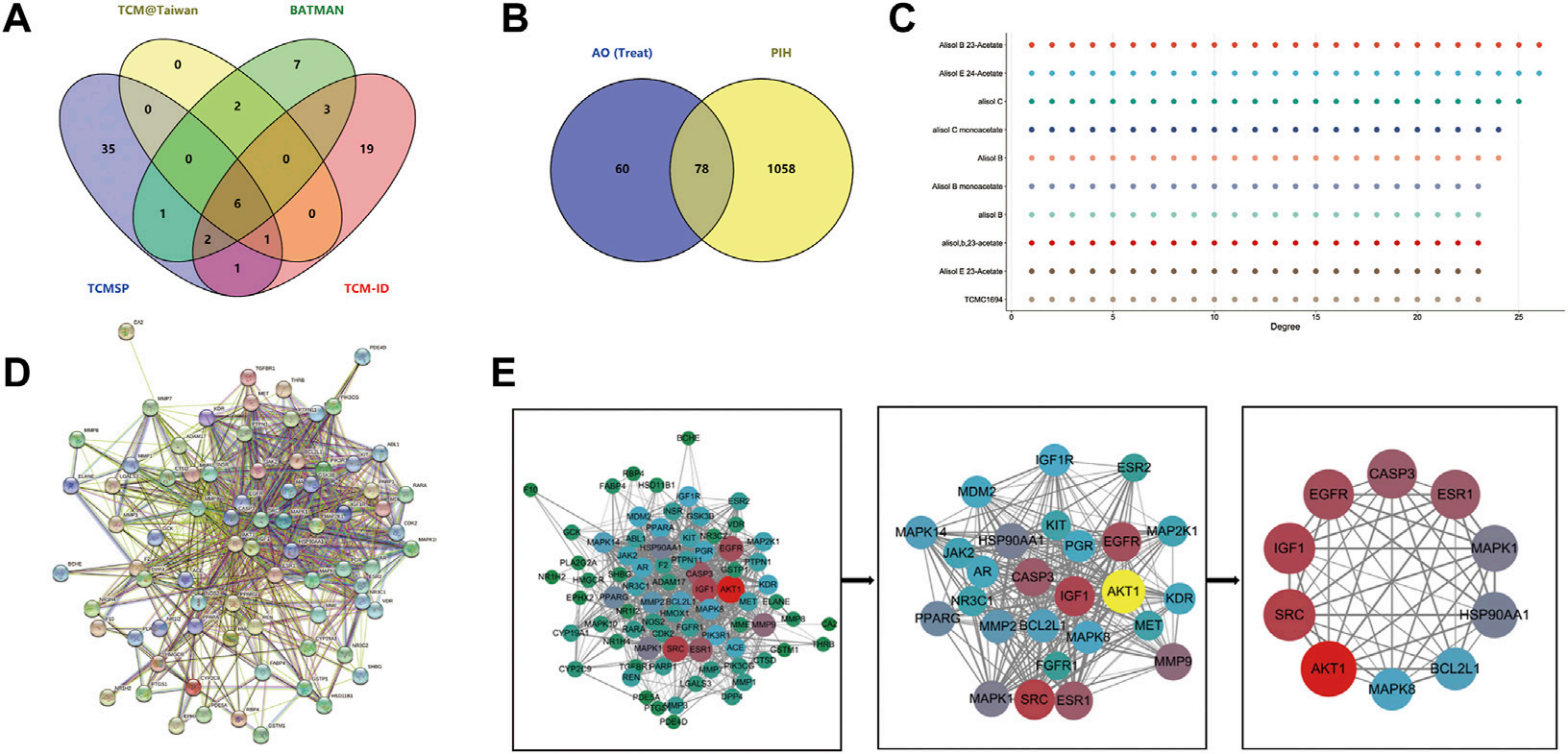
The PPI network of AO active compounds in the treatment of PIH **(A)**, Venn diagram of AO identified compounds from TCMSP, TCM-ID, TCM@Taiwan and BATMAN-TCM database **(B)**, Venn diagram of potential targets to the AO treat PIH **(C)**, The degree value of compound potential targets **(D)**, The 78 targets PPI network gain from STRING database **(E)**, Plug-in of Cytoscape to screen core targets.

There were 77 compounds of AO obtained from TCMSP, TCM-ID, TCM@Taiwan and BATMAN database (Figure 3A) (Details were listed in Supplementary Table S1). Based on the screening criteria OB ≥ 30 and DL ≥ 0.18, 10 eligible active compounds were obtained from the TCMSP database, including sitosterol (MOL000359), Alisol B (MOL000830), Alisol B monoacetate (MOL000831), alisol b 23 acetate (MOL000832), 16β-methoxyalisol B monoacetate (MOL000849), alisol B (MOL000853), alisol C (MOL000854), alisol C monoacetate (MOL000856), 1-Monolinolein (MOL002464) and Alisol B acetate (MOL000862). There were 19 bioactive compounds from other database sources, including 13Β,17Β-Epoxyalisol A, 24-Deacetylalisol O, 25-Anhydroalisol F, Alismol, Alisol B 23-Acetate, Alisol E 23-Acetate, Alisol E 24-Acetate, Alizexol A, Neoalisol, Oriediterpenol, Oriediterpenoside and other eight compounds without formally name. The 10 toxic compounds retrieved from TOXNET and CTD databases were choline (MOL000394), emodin (MOL000472), 5-hydroxymethylfurfural (HMF, MOL000748), 1 h-indole-3-carboxylic acid (MOL000823), sucrose (MOL000842), nicotinamide (NCA, MOL000857), stearic acid (MOL000860), Healip (MOL000861), 2-Furaldehyde and 2-Furancarboxylic acid (details shown in Table1, chemistry structure shown in Figure 2).

**TABLE 1:** The chemical characteristics of 39 AO bioactive compounds obtained in TCMSP, TCM-ID, TCM@Taiwan, and BATMAN-TCM database.

No.	Molecule name	Molecular weight	InCHIKey ID	Compound	OB (%)	DL	Source
1	1-Monolinolein	354.59	WECGLUPZRHILCT-GSNKCQISSA-N	Treat	37.18	0.3	TCMSP
2	Sitosterol	414.79	KZJWDPNRJALLNS-ZFVHJZABSA-N	Treat	36.91	0.75	TCMSP
3	Alisol B	444.72	XOWUWSRAQZUPJP-ASMOWKBLSA-N	Treat	36.76	0.82	TCMSP
4	Alisol B monoacetate	514.82	NLOAQXKIIGTTRE-JOGPTBIUSA-N	Treat	35.58	0.81	TCMSP
5	Alisol B Acetate	514.82	NLOAQXKIIGTTRE-JSWHPQHOSA-N	Treat	35.58	0.81	TCMSP
6	Alisol B	472.78	GBJKHDVRXAVITG-HKXAQQBESA-N	Treat	34.47	0.82	TCMSP
7	Alisol C monoacetate	514.77	KOOCQNIPRJEMDH-QSKXMHMESA-N	Treat	33.06	0.83	TCMSP
8	Alisol C	486.76	DORJGGFFCMZTHW-KXVAGGRESA-N	Treat	32.7	0.82	TCMSP
9	Alisol,b,23-acetate	446.74	QIMPSOXWCKBEBD-PQZUBQKGSA-N	Treat	32.52	0.82	TCMSP
10	16β-methoxyalisol B monoacetate	544.85	UJRPGLKPBYUOLM-XBKPOWCMSA-N	Treat	32.43	0.77	TCMSP
11	NCA	122.14	DFPAKSUCGFBDDF-UHFFFAOYSA-N	Toxic	71.13	0.02	TCMSP
12	HMF	126.12	NOEGNKMFWQHSLB-UHFFFAOYSA-N	Toxic	45.07	0.02	TCMSP
13	Choline	104.2	GDPPXFUBIJJIKR-UHFFFAOYSA-N	Toxic	0.47	0.01	TCMSP
14	Healip	326.68	NOPFSRXAKWQILS-UHFFFAOYSA-N	Toxic	11.65	0.22	TCMSP
15	Emodin	270.25	RHMXXJGYXNZAPX-UHFFFAOYSA-N	Toxic	24.4	0.24	TCMSP
16	1h-indole-3-carboxylic,acid	161.17	KUQQAVBKLJHQJI-UHFFFAOYSA-O	Toxic	25.83	0.05	TCMSP
17	Stearic acid	284.54	QIQXTHQIDYTFRH-UHFFFAOYSA-N	Toxic	17.83	0.14	TCMSP
18	Sucrose	342.34	CZMRCDWAGMRECN-UGDNZRGBSA-N	Toxic	7.17	0.23	TCMSP
NO.	Molecule name	Molecular weight	InCHIKey ID	Compound	GI absorption	Lipinski RO5	Source
19	13Β,17Β-Epoxyalisol A	506.7	BLYMKZZHAHHGBK-KOMKTNACSA-N	Treat	High	Yes	TCM-ID
20	24-Deacetylalisol O	470.34	ZQFQEWOYCZTQFY-OZBSICFQSA-N	Treat	High	Yes	TCM-ID
21	25-Anhydroalisol F	470.34	MRBFVJVQQIVGMY-CDJAXXMBSA-N	Treat	High	Yes	TCM-ID
22	Alismol	220.35	BUPJOLXWQXEJSQ-GIJJTGMTSA-N	Treat	High	Yes	BATMAN
23	Alisol B 23-Acetate	516.35	CPYFCYMXHGAZTF-WXYIYAPGSA-N	Treat	High	Yes	TCM-ID
24	Alisol E 23-Acetate	532.8	KRZLECBBHPYBFK-GLHMJAHESA-N	Treat	High	Yes	TCM-ID
25	Alisol E 24-Acetate	532.8	WXHUQVMHWUQNTG-GLHMJAHESA-N	Treat	High	Yes	TCM-ID
26	Alizexol A	530.7	KFWYQAKZMXFEFB-XKFNBYHKSA-N	Treat	High	Yes	TCM-ID
27	Neoalisol	488.7	QOEBTWRYOBEBPF-FZUOWIMQSA-N	Treat	High	Yes	TCM-ID
28	Oriediterpenol	304.5	ZALNTAHRBOFRCM-SVGWTELYSA-N	Treat	High	Yes	BATMAN
29	Oriediterpenoside	436.6	ITBIOEXYDKFWDZ-SUMMCVSUSA-N	Treat	High	Yes	BATMAN
30	TCMC1688	484.32	GHJSMGTZWLBPHU-DSAUVPRUSA-N	Treat	High	Yes	TCM-ID
31	TCMC1689	468.32	MPDDCFALNXXKHF-AJFHEMKZSA-N	Treat	High	Yes	TCM-ID
32	TCMC1691	518.4	QVVRXENOONWNGX-IPAXGOGQSA-N	Treat	High	Yes	TCM-ID
33	TCMC1692	488.35	OVJNKAWWCRMIMP-UNPOXIGHSA-N	Treat	High	Yes	TCM-ID
34	TCMC1694	474.33	DCYOPZMJKBYLCO-QJDZRUNDSA-N	Treat	High	Yes	TCM-ID
35	TCMC1695	486.33	PGQUPZCFLHXEHV-KPYCMCGFSA-N	Treat	High	Yes	TCM-ID
36	TCMC1696	530.36	QJQGTCCCAJRIPR-QRPPABIJSA-N	Treat	High	Yes	TCM-ID
37	TCMC4823	254.36	JNTOHIOAISZSEJ-SAAWNECCSA-N	Treat	High	Yes	TCM-ID
38	2-Furaldehyde	96.08	HYBBIBNJHNGZAN-UHFFFAOYSA-N	Toxic	High	Yes	TCM-ID
39	2-Furancarboxylic acid	112.08	SMNDYUVBFMFKNZ-UHFFFAOYSA-N	Toxic	High	Yes	TCM-ID/TCM@Taiwan

OB, oral bioavailability; DL, drug-likeness; GI absorption, gastrointestinal absorption; HMF, 5-Hydroxymethyl-2-furaldehyde; NCA, nicotinamide; TCMC1688, (24R)-24,25-Dihydroxyprotosta-11,13(17)-Diene-3,16,23-Trione; TCMC1689, (23S,24R)-23,24-Dihydroxyprotosta-11,13(17),25-Triene-3,16-Dione; TCMC1691, (23S,24R)-11beta,23,24-Trihydroxy-25-Ethoxyprotosta-13(17)-Ene-3-One; TCMC1692, (23S,24R)-11beta,23,24,25-Tetrahydroxyprotosta-13(17),15-Diene-3-One; TCMC1694, (23S,24R)-11beta,23,24,25-Tetrahydroxy-29-Nor-3,4-Didehydroprotosta-13(17)-Ene-2-One; TCMC1695, (5R,8S,9S,10S,14R)-4,4,8,10,14-pentamethyl-17-[(2R,4S,5S)-4,5,6-trihydroxy-6-methylheptan-2-yl]-2,5,6,7,9,15-hexahydro-1H-cyclopenta[a]phenanthrene-3,16-dione; TCMC1696, [(1S,2R,4S,6S,7S,9R,13S,14S,15S,20R)-13-hydroxy-6-(2-hydroxypropan-2-yl)-1,2,9,15,19,19-hexamethyl-18-oxo-5-oxapentacyclo[12.8.0.02,11.04,10.015,20]docos-10-en-7-yl]; TCMC4823, (1S,2S,5R,6S,7R,8S)-1,5-dimethyl-8-propan-2-yl-11-oxatricyclo[6.2.1.02,6]undecane-5,7-diol.

After screening and removing duplicates, we identified 138 potential targets associated with 29 therapeutic compounds and 160 targets associated with 10 AO toxic compounds. Besides, 1136 putative targets of PIH, 2188 targets of drug-induced liver injury and 2094 targets of drug-induced kidney injury were retrieved from the GeneCards, DisGeNET and OMIM databases. 78 targets overlapped between therapeutic compounds of AO and PIH, while 117 and 111 targets overlapped between AO toxic compounds and liver and kidney injury were obtained by Venn (detailed targets provided in Table 2).

**TABLE 2:** The detail gene name of potential targets of AO in the treatment of PIH and AO toxic compounds induced hepato-nephrotoxicity.

No.	Target	Symbol	Entrez ID	Compound	NO.	Target	Symbol	Entrez ID	Compound
1	Angiotensin-converting enzyme	ACE	P12821	Treat/Toxic	79	Acetylcholinesterase	ACHE	P22303	Toxic
2	Androgen receptor	AR	P10275	Treat/Toxic	80	Long-chain-fatty-acid--CoA ligase 1	ACSL1	P33121	Toxic
3	Cholinesterase	BCHE	P06276	Treat/Toxic	81	Actin, aortic smooth muscle	ACTA2	P62736	Toxic
4	Carbonic anhydrase 2	CA2	P00918	Treat/Toxic	82	All-trans-retinol dehydrogenase	ADH1B	P00325	Toxic
5	Caspase-3	CASP3	P42574	Treat/Toxic	83	Alcohol dehydrogenase 1C	ADH1C	P00326	Toxic
6	Cyclin-dependent kinase 2	CDK2	P24941	Treat/Toxic	84	Adenosylhomocysteinase	AHCY	P23526	Toxic
7	Aromatase	CYP19A1	P11511	Treat/Toxic	85	Aldo-keto reductase family 1 member B1	AKR1B1	P15121	Toxic
8	Dipeptidyl peptidase 4	DPP4	P27487	Treat/Toxic	86	Aldehyde dehydrogenase, mitochondrial	ALDH2	P05091	Toxic
9	Epidermal growth factor receptor	EGFR	P00533	Treat/Toxic	87	Arginase-1	ARG1	P05089	Toxic
10	Neutrophil elastase	ELANE	P08246	Treat/Toxic	88	Beta-secretase 1	BACE1	P56817	Toxic
11	Bifunctional epoxide hydrolase 2	EPHX2	P34913	Treat/Toxic	89	Tyrosine-protein kinase BTK	BTK	Q06187	Toxic
12	Estrogen receptor	ESR1	P03372	Treat/Toxic	90	Cyclin-A2	CCNA2	P20248	Toxic
13	Estrogen receptor beta	ESR2	Q92731	Treat/Toxic	91	Cyclin-dependent kinase 6	CDK6	Q00534	Toxic
14	Coagulation factor X	F10	P00742	Treat/Toxic	92	Cyclin-dependent kinase inhibitor 1	CDKN1A	P38936	Toxic
15	Prothrombin	F2	P00734	Treat/Toxic	93	Liver carboxylesterase 1	CES1	P23141	Toxic
16	Fatty acid-binding protein, adipocyte	FABP4	P15090	Treat/Toxic	94	Serine/threonine-protein kinase Chk1	CHEK1	O14757	Toxic
17	Glycogen synthase kinase-3 beta	GSK3B	P49841	Treat/Toxic	95	Chitinase-3-like protein 1	CHI3L1	P36222	Toxic
18	3-hydroxy-3-methylglutaryl-coenzyme A reductase	HMGCR	P04035	Treat/Toxic	96	Muscarinic acetylcholine receptor M1	CHRM1	P11229	Toxic
19	11-beta-hydroxysteroid dehydrogenase 1	HSD11B1	P28845	Treat/Toxic	97	Muscarinic acetylcholine receptor M2	CHRM2	P08172	Toxic
20	Heat shock protein HSP 90-alpha	HSP90AA1	P07900	Treat/Toxic	98	Muscarinic acetylcholine receptor M3	CHRM3	P20309	Toxic
21	Tyrosine-protein kinase JAK2	JAK2	O60674	Treat/Toxic	99	Collagen alpha-1(I) chain	COL1A1	P02452	Toxic
22	Vascular endothelial growth factor receptor 2	KDR	P35968	Treat/Toxic	100	Collagen alpha-1(VII) chain	COL7A1	Q02388	Toxic
23	Mast/stem cell growth factor receptor Kit	KIT	P10721	Treat/Toxic	101	Granulocyte-macrophage colony-stimulating factor	CSF2	P04141	Toxic
24	Galectin-3	LGALS3	P17931	Treat/Toxic	102	Casein kinase II subunit alpha	CSNK2A1	P68400	Toxic
25	Mitogen-activated protein kinase 14	MAPK14	Q16539	Treat/Toxic	103	Cytochrome P450 1A1	CYP1A1	P04798	Toxic
26	Mitogen-activated protein kinase 8	MAPK8	P45983	Treat/Toxic	104	Cytochrome P450 1A2	CYP1A2	O77810	Toxic
27	Hepatocyte growth factor receptor	MET	P08581	Treat/Toxic	105	Dihydroorotate dehydrogenase (quinone)	DHODH	Q02127	Toxic
28	Interstitial collagenase	MMP1	P03956	Treat/Toxic	106	Pro-epidermal growth factor	EGF	P01133	Toxic
29	72 kDa type IV collagenase	MMP2	P08253	Treat/Toxic	107	Coagulation factor VII	F7	P08709	Toxic
30	Stromelysin-1	MMP3	P08254	Treat/Toxic	108	Vascular endothelial growth factor receptor 1	FLT1	P17948	Toxic
31	Matrix metalloproteinase-9	MMP9	P14780	Treat/Toxic	109	Vascular endothelial growth factor receptor 3	FLT4	P35916	Toxic
32	Oxysterols receptor LXR-beta	NR1H2	P55055	Treat/Toxic	110	Glutamate carboxypeptidase 2	FOLH1	Q04609	Toxic
33	Bile acid receptor	NR1H4	Q96RI1	Treat/Toxic	111	Protein c-Fos	FOS	P01100	Toxic
34	Glucocorticoid receptor	NR3C1	P59667	Treat/Toxic	112	Glucose-6-phosphatase catalytic subunit 1	G6PC1	P35575	Toxic
35	Mineralocorticoid receptor	NR3C2	P08235	Treat/Toxic	113	Gamma-aminobutyric acid receptor subunit alpha-1	GABRA1	P14867	Toxic
36	Poly [ADP-ribose] polymerase 1	PARP1	P09874	Treat/Toxic	114	Gamma-aminobutyric acid receptor subunit alpha-2	GABRA2	P47869	Toxic
37	Progesterone receptor	PGR	P06401	Treat/Toxic	115	Glutamine synthetase	GLUL	P15104	Toxic
38	Phosphatidylinositol 4,5-bisphosphate 3-kinase catalytic subunit gamma isoform	PIK3CG	P48736	Treat/Toxic	116	Glutamate receptor 2	GRIA2	P42262	Toxic
39	Peroxisome proliferator-activated receptor alpha	PPARA	Q07869	Treat/Toxic	117	Glutathione reductase, mitochondrial	GSR	P00390	Toxic
40	Peroxisome proliferator-activated receptor gamma	PPARG	P37231	Treat/Toxic	118	Histone deacetylase 8	HDAC8	Q9BY41	Toxic
41	Prostaglandin G/H synthase 1	PTGS1	P23219	Treat/Toxic	119	Hexokinase-1	HK1	P19367	Toxic
42	Tyrosine-protein phosphatase non-receptor type 1	PTPN1	P18031	Treat/Toxic	120	Immunoglobulin heavy constant gamma 1	IGHG1	P01857	Toxic
43	Retinoic acid receptor alpha	RARA	P10276	Treat/Toxic	121	Interleukin-1 beta	IL1B	P01584	Toxic
44	Retinol-binding protein 4	RBP4	P02753	Treat/Toxic	122	Interleukin-2	IL2	P60568	Toxic
45	Sex hormone-binding globulin	SHBG	P04278	Treat/Toxic	123	Integrin alpha-L	ITGAL	P20701	Toxic
46	Vitamin D3 receptor	VDR	F8VRJ4	Treat/Toxic	124	Kinesin-like protein KIF11	KIF11	P52732	Toxic
47	Tyrosine-protein kinase ABL1	ABL1	P00519	Treat	125	Tyrosine-protein kinase Lck	LCK	P06239	Toxic
48	Disintegrin and metalloproteinase domain-containing protein 17	ADAM17	P78536	Treat	126	Lysozyme C	LYZ	P61626	Toxic
49	RAC-alpha serine/threonine-protein kinase	AKT1	P31749	Treat	127	Amine oxidase [flavin-containing] B	MAOB	P27338	Toxic
50	Bcl-2-like protein 1	BCL2L1	Q07817	Treat	128	Macrophage migration inhibitory factor	MIF	P14174	Toxic
51	Cathepsin D	CTSD	P07339	Treat	129	Macrophage metalloelastase	MMP12	P39900	Toxic
52	Cytochrome P450 2C9	CYP2C9	P11712	Treat	130	Myc proto-oncogene protein	MYC	P01106	Toxic
53	Fibroblast growth factor receptor 1	FGFR1	P11362	Treat	131	Nuclear receptor subfamily 1 group I member 3	NR1I3	Q14994	Toxic
54	Hexokinase-4	GCK	P35557	Treat	132	Serine/threonine-protein kinase pim-1	PIM1	P11309	Toxic
55	Glutathione S-transferase Mu 1	GSTM1	P09488	Treat	133	Serine/threonine-protein kinase PLK1	PLK1	P53350	Toxic
56	Glutathione S-transferase P	GSTP1	P09211	Treat	134	Peroxisome proliferator-activated receptor delta	PPARD	Q03181	Toxic
57	Heme oxygenase 1	HMOX1	P09601	Treat	135	cAMP-dependent protein kinase catalytic subunit alpha	PRKACA	P17612	Toxic
58	Insulin-like growth factor I	IGF1	P05019	Treat	136	Protein kinase C delta type	PRKCD	Q05655	Toxic
59	Insulin-like growth factor 1 receptor	IGF1R	P08069	Treat	137	Protein kinase C epsilon type	PRKCE	Q02156	Toxic
60	Insulin receptor	INSR	P06213	Treat	138	Prostaglandin G/H synthase 2	PTGS2	P27607	Toxic
61	Dual specificity mitogen-activated protein kinase kinase 1	MAP2K1	Q02750	Treat	139	Glycogen phosphorylase, liver form	PYGL	P06737	Toxic
62	Mitogen-activated protein kinase 1	MAPK1	P28482	Treat	140	RAF proto-oncogene serine/threonine-protein kinase	RAF1	P04049	Toxic
63	Mitogen-activated protein kinase 10	MAPK10	P53779	Treat	141	Retinoic acid receptor beta	RARB	P10826	Toxic
64	E3 ubiquitin-protein ligase Mdm2	MDM2	Q00987	Treat	142	Retinoic acid receptor RXR-alpha	RXRA	P19793	Toxic
65	Neprilysin	MME	P08473	Treat	143	Solute carrier family 2, facilitated glucose transporter member 1	SLC2A1	P11166	Toxic
66	Matrilysin	MMP7	P09237	Treat	144	Solute carrier family 2, facilitated glucose transporter member 4	SLC2A4	P14672	Toxic
67	Neutrophil collagenase	MMP8	P22894	Treat	145	Transcription factor Sp1	SP1	P08047	Toxic
68	Nitric oxide synthase, inducible	NOS2	P35228	Treat	146	Sterol regulatory element-binding protein 1	SREBF1	P36956	Toxic
69	Nuclear receptor subfamily 1 group I member 2	NR1I2	O75469	Treat	147	Tyrosine-protein kinase SYK	SYK	P43405	Toxic
70	cAMP-specific 3′,5′-cyclic phosphodiesterase 4D	PDE4D	Q08499	Treat	148	Transforming growth factor beta-1 proprotein	TGFB1	P01137	Toxic
71	cGMP-specific 3′,5′-cyclic phosphodiesterase	PDE5A	O76074	Treat	149	Tumor necrosis factor	TNF	P01375	Toxic
72	Phosphatidylinositol 3-kinase regulatory subunit alpha	PIK3R1	P27986	Treat	150	DNA topoisomerase 2-alpha	TOP2A	P11388	Toxic
73	Phospholipase A2, membrane associated	PLA2G2A	P14555	Treat	151	Cellular tumor antigen p53	TP53	P04637	Toxic
74	Tyrosine-protein phosphatase non-receptor type 11	PTPN11	Q90687	Treat	152	Transthyretin	TTR	P02766	Toxic
75	Renin	REN	P00797	Treat	—	—	—	—	—
76	Proto-oncogene tyrosine-protein kinase Src	SRC	P12931	Treat	—	—	—	—	—
77	TGF-beta receptor type-1	TGFBR1	P36897	Treat	—	—	—	—	—
78	Thyroid hormone receptor beta	THRB	P10828	Treat	—	—	—	—	—

### PPI network analysis

The 78 targets of AO in the treatment of PIH were incorporated into the STRING database to construct the PPI network. 78 nodes and 771 edges of protein-protein interaction were visually displayed in Cytoscape software, topological analysis including degree centrality (DC) and combined score, and the maximal clique centrality (MCC) algorithm was further used to screen the core targets. The top 10 targets were AKT1, BCL2L1, CASP3, EGFR, ESR1, HSP90AA1, IGF1, MAPK1, MAPK8 and SRC (Figure 3). Besides, after removing the disconnect node, the PPI network of toxic compounds-induced liver injury included 116 nodes and 1176 edges, the top 10 targets were CASP3, EGF, EGFR, ESR1, HSP90AA1, IL1B, MYC, PPARG, TNF and TP53 (Figures 5A,B). And the PPI network of toxic compounds-induced kidney injury included 109 nodes and 1130 edges, the top 10 targets were CASP3, EGF, EGFR, FOS, HSP90AA1, MMP9, MYC, PTGS2, TNF and TP53 (Supplementary Figure S1A,B).

### GO and KEGG pathway enrichment analysis

**FIGURE 4:**
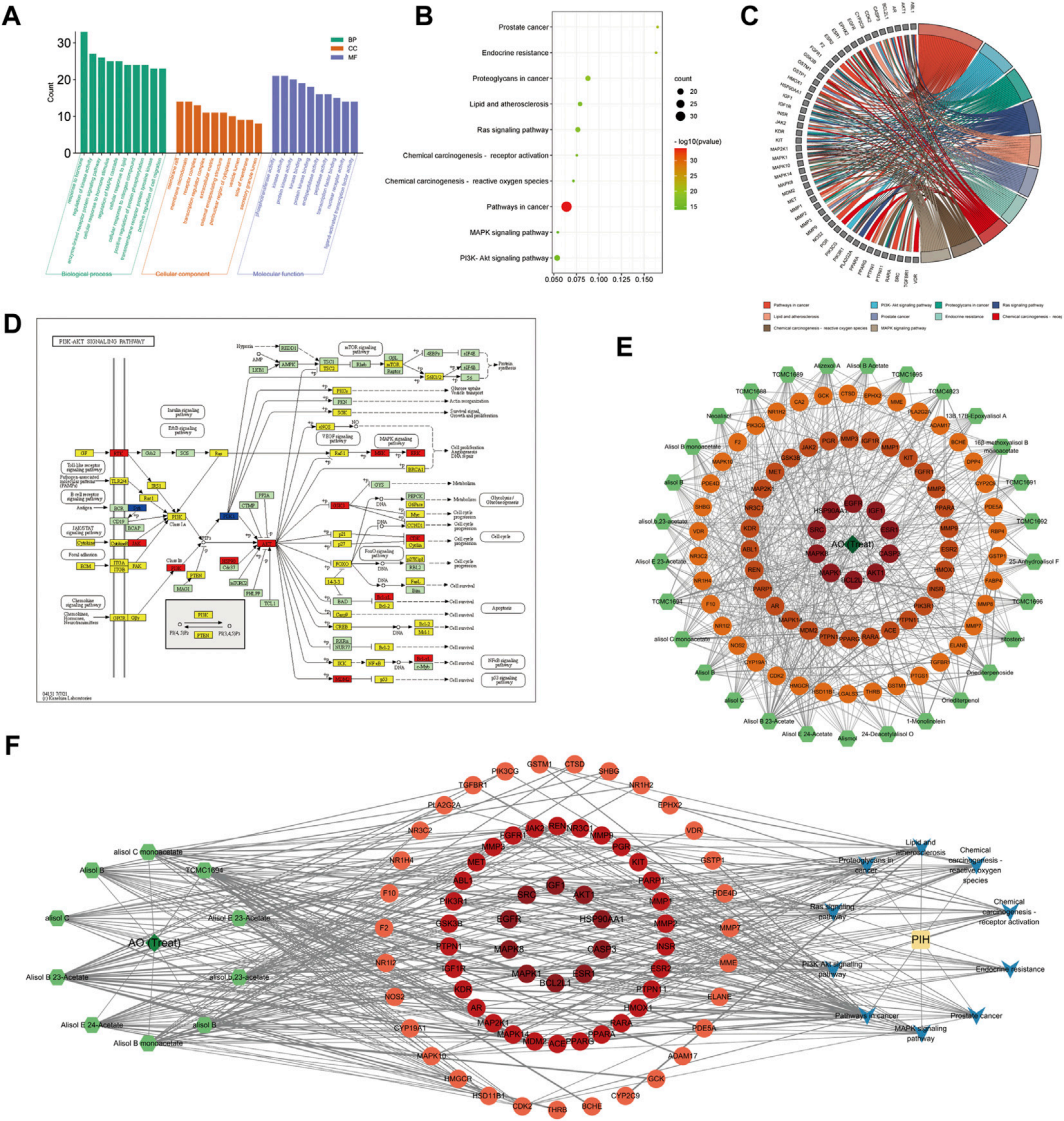
GO/KEGG enrichment analysis of AO treat PIH **(A)**, The bar graph of top 10 GO items, including biological process (BP), cellular compound (CC) and molecular function (MF) **(B)**, The bubble diagram of the top 10 KEGG pathway **(C)**, GO chord chart **(D)**, The core pathway, PI3K-AKT cascade mapped by KEGG Mapper database, whereby therapeutic targets were colored in red, targets of AO but not in the treatment of PIH were in blue, the other targets of PIH were yellow **(E)**, The H-C-T network map showed the importance of each AO compounds, whereby 29 compounds ranked by degree value **(F)**, The H-C-T-P-D network map showed the complex regulation of AO treat PIH. The deep green diamond represents herb (AO), the light green hexagon represents the therapeutic compound of AO, the red and orange circles represent targets based on the degree value, the blue V icon represents the pathway involved, and the yellow rectangle is the disease (PIH).

A total of 1022 biological process, 50 cellular compound and 93 molecular function items were obtained from the Metascape database, and 151 KEGG pathways were significantly enriched (*p* < 0.01, *q* < 0.05), demonstrating the characteristic of multi-pathway of AO in the treatment of PIH. The top 10 biological processes included the response to hormone, regulation of kinase activity, enzyme-linked receptor protein signaling pathway, cellular response to hormone stimulus, regulation of MAPK cascade cellular response to lipid, cellular response to nitrogen compound, positive regulation of protein phosphorylation, transmembrane receptor protein tyrosine kinase and positive regulation of cell migration (Figures 4A–C).

**FIGURE 5:**
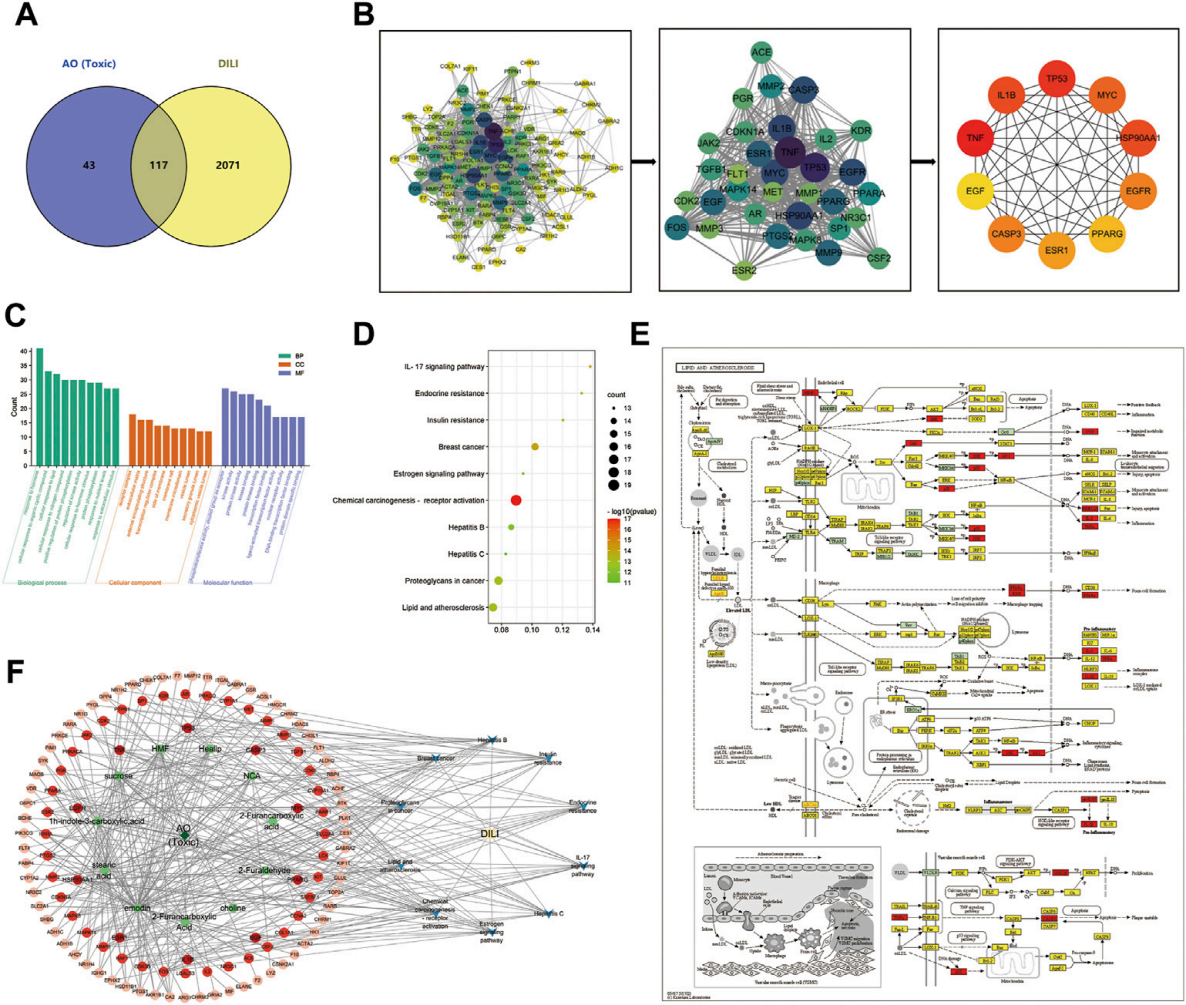
The PPI network and GO/KEGG enrichment analysis of AO-induced liver injury **(A)**, Venn diagram of potential targets to the AO-induced hepatotoxicity **(B)**, The 116 targets PPI network and plug-in of Cytoscape to screen core targets **(C)**, The bar graph of top 10 BP, CC and MF items **(D)**, The bubble diagram of the top 10 KEGG pathway **(E)**, The lipid and atherosclerosis pathway mapped by KEGG Mapper database **(F)**, The H-C-T-P-D network showed the regulation mechanism of AO induced liver injury.

The top 10 significantly enriched KEGG pathways included Pathways in cancer, MAPK signaling pathway, PI3K-Akt signaling pathway, Proteoglycans in cancer, Prostate cancer, Lipid and atherosclerosis, Ras signaling pathway, Endocrine resistance, Chemical carcinogenesis -receptor activation and reactive oxygen species. Based on the enrichment ratio of pathways and their correlation with diseases, the PI3K-Akt signaling pathway was the most important. We further colored the detailed targets of the PI3K-Akt pathway in the KEGG mapper database, whereby therapeutic targets were colored in red, targets of AO but not in the treatment of PIH were in blue, the other targets of PIH were yellow (Figure 4D). Besides, a total of 1262 biological process, 55 cellular compound and 117 molecular function and 168 KEGG pathway items were significantly enriched (*p* < 0.01, *q* < 0.05), and the lipid and atherosclerosis pathway was significantly associated with AO induced liver injury (Figures 5C–E). And a total of 1260BP, 52 CC, 115 MF items, and 161 KEGG pathway were enriched (*p* < 0.01, *q* < 0.05), the lipid and atherosclerosis pathway was also significantly associated with AO induced kidney injury (Supplementary Figures S1C,E).

### Herb-compound-target-pathway-disease network analysis

After identifying the core targets and the top 10 pathways from previous analysis, a “herb-compound-target-pathway-disease” (H-C-T-P-D) network was constructed by Cytoscape 3.9.1 to reveal the complex molecular mechanism and multi-compound, multi-target and multi-pathway characteristic of AO in the treatment of PIH (Figures 4E,F). The major active compounds from the network were Alisol B 23-Acetate, Alisol E 24-Acetate and alisol C. The deep green diamond represents herb (AO), the light green hexagon represents the therapeutic compound of AO, the red and orange circles represent targets based on the degree value, the blue V icon represents the pathway involved, and the yellow rectangle is the disease (PIH). Similarly, the “H-C-T-P-D” network was used to illustrate the mechanism of AO toxic compounds induced liver injury (Figure 5F) and kidney injury (Supplementary Figure S1F), whereby the green diamond, the light green hexagon, the red circle, the blue Vicon and the yellow rectangle represent the herb, the toxic compounds of AO, targets, pathways, and disease (AO induced hepato-nephrotoxicity). The main toxic compound was emodin (MOL000472).

### Molecular docking

**FIGURE 6:**
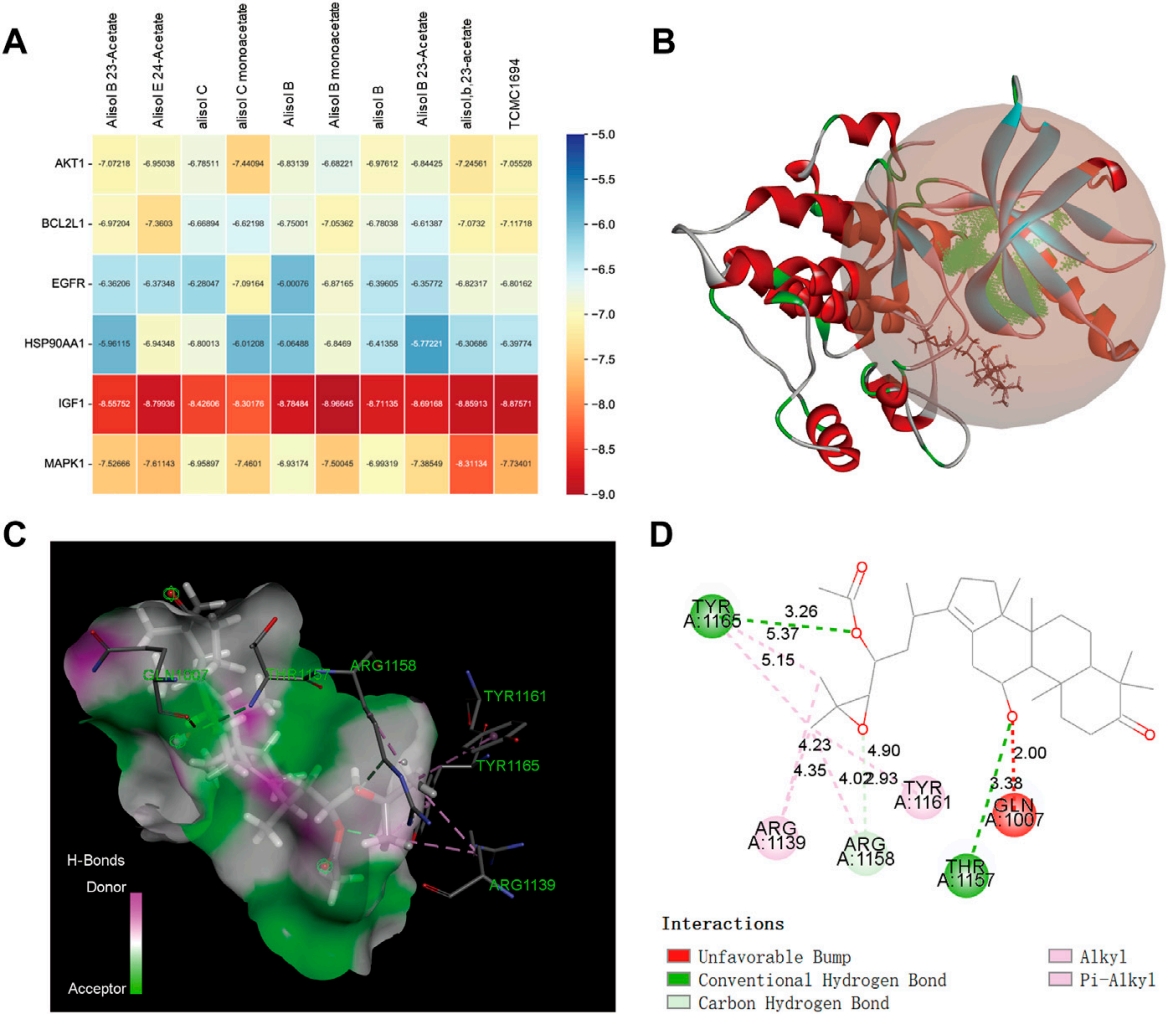
The molecular docking results of AO therapeutic compounds with core targets **(A)**, The binding affinity heatmap of ligand-receptor complex **(B)**, 3D structures of the Alisol B monoacetate-IGF1 complex **(C)**, spatial structure of Alisol B monoacetate-IGF1 show binding details **(D)**, 2D structure of Alisol B monoacetate-IGF1 complex show intermolecular force types.

**FIGURE 7:**
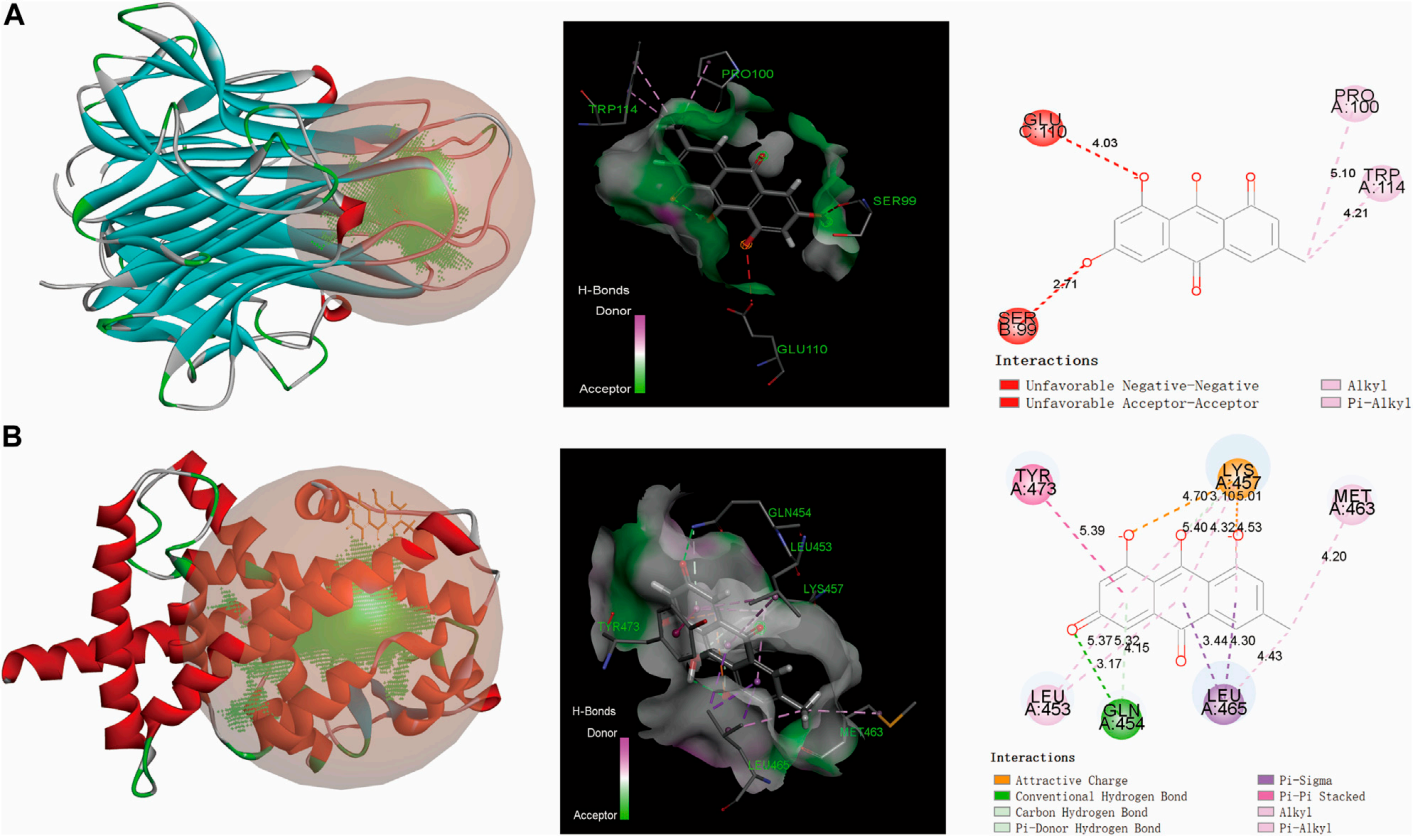
The molecular docking results of AO toxic compounds with corresponding core targets **(A)**, emodin docking with TNF **(B)**, emodin docking with PPARG (From left to right: 3D, spatial and 2D structure).

Supplementary Table S2 exhibits the binding affinity of the top 10 therapeutic compounds and emodin with their core targets, respectively. The results showed that the active compounds had good binding activities with all targets. A binding affinity < -5.0 kcal/mol indicated that the small molecule ligand has a good binding activity with the receptor protein, and binding affinity < -7.0 kcal/mol indicated stronger binding activity. Among them, we found a binding affinity < -7.0 kcal/mol between all compounds and IGF1 as well as emodin with TNF and PPARG, indicating that IGF1 may be an important target mediating the therapeutic effect against PIH, and PPARG and TNF may be the main targets of AO-induced liver injury. Figure 6A was the binding affinity heatmap of ligand-protein complexes, and Figures 6B–D shows the details of molecular docking between Alisol B monoacetate and IGF1 (lowest binding affinity); the intermolecular forces include alkyl bonding, π-alkyl bonding, conventional hydrogen bonding and carbon-hydrogen bonding. Figure 7 shows the results of emodin binding with PPARG and TNF.

### Molecular dynamics simulation

**FIGURE 8:**
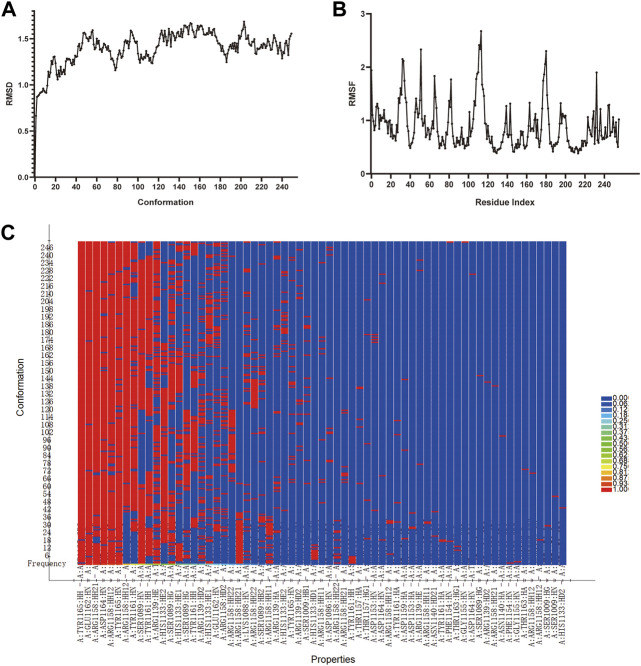
The molecular dynamic simulations results of Alisol B monoacetate binding with IGF1 **(A)**, RMSD and **(B)**, RMSF curves of MDS **(C)**, Hydrogen bond heat map of Alisol B monoacetate binding with IGF1.

Molecular dynamics simulation represents an important technology for analyzing the ligand-protein complex’s conformational change and stability after docking. To explore the binding stability of Alisol B monoacetate with IGF1, the RMSD, RMSF curves, energies alternate tendency and hydrogen bonding heat map were calculated. Figure 8 shows that after 50 ps, the trajectories of all molecules and energy levels tend to stabilized. The RMSD, RMSF curve and hydrogen bond heat map exhibited good stability.

## Discussion

Pregnancy-induced hypertension is a common disease during pregnancy, with a 10–12% incidence rate (Lan et al., 2022). Elevated arterial pressure and proteinuria are the most prominent clinical manifestations, and disease progression can cause reversible pathological damage to multiple systems and organs. Given the importance of the safety profile of drugs indicated for pregnant women and the unknown pathogenesis of PIH, significant emphasis has been placed on better understanding the underlying therapeutic and toxic mechanisms of these drugs (Reddy et al., 2022).

As mentioned above, oxidative stress, immune dysregulation, endothelial cell malfunction and other perplex factors have been associated with the pathogenesis of PIH. Unlike Western medicine which adopts a targeted approach, TCM fosters a more holistic approach based on the multi-compound, multi-target and multi-pathway of TCM drugs and has huge prospects for use as complementary and alternative medicine.

Alisma Orientale, also known as “Ze Xie” in Chinese, is the most important herbal compound in Ze Xie decoction indicated for treating hypertension, according to ancient records. Modern studies have isolated more than 100 active compounds, mainly terpenoids and small amounts of flavonoids, alkaloids, phytosterols and fatty acids. It is worth mentioning that protostane triterpenoids are the characteristic compounds, including alisol A-I and their derivatives (Shu et al., 2016). It has been established that Alisol B 23-acetate is an important compound with excellent bioactivities used to characterize AO in the “Pharmacopoeia of the People’s Republic of China” (Li et al., 2019b). Our findings showed that compounds with therapeutic effects included Alisol B 23-acetate, Alisol E 24-acetate and other derivatives, consistent with the “H-C-T-P-D” network.

Previous studies have demonstrated that AO plants, especially triterpene compounds, have diuretic activity. Zhang et al. reported that Alisol B, alisol B 23-acetate and other derivatives could interfere with the Na+, K+, and Cl− co-transport carrier on the luminal membrane of the Henle and sodium–chloride co-transporter in the renal distal convoluting tubule to exert diuretic action (Zhang et al., 2017). It could also significantly affect K+ excretion by competitively binding the receptor site in the collecting tube to influence sodium, potassium exchange and acid absorption. Besides, the immunomodulatory activity of the methanol extract of triterpenoids from the AO plant (alisol A, B and their acetate derivatives) significantly inhibited paw swelling in rats showing type I-IV hypersensitivity (Shao et al., 2018). In addition, alisol B and alisol B monoacetate could inhibit the complement system through the antigen–antibody-mediated process, exhibiting a good therapeutic effect against immune-related diseases (Lee et al., 2003). Further studies illustrated that alisol B 23-acetate could prevent lipid peroxidation and regulate inducible nitric oxide synthase (iNOS) expression, with significant reticuloendothelial system-potentiating activities (Matsuda et al., 1999). In conclusion, the active compounds of AO possess diuretic, immunomodulatory and vascular endothelial modulatory activities, exhibiting multi-compound, multi-target and cooperation characteristics as well as multi-compound amplification effects in the treatment of PIH.

Few studies have hitherto assessed the toxicity and side effects of AO and its compounds to determine its biosafety profile (Tian et al., 2014). Accordingly, network toxicology was applied to explore the potential mechanism in this research. Our findings showed that emodin belonging to flavonoids was the main compound that induced hepato-nephrotoxicity, and other toxic compounds with smaller proportions, including fatty acids and carbohydrates, such as stearic acid and sucrose. PPI network and GO/KEGG pathway enrichment analysis showed that AO toxic compounds mainly targeted PPARG and TNF and regulated the lipid and atherosclerosis signaling pathway. PPARG, also known as peroxisome proliferator-activated receptor gamma, is a nuclear receptor binding peroxisome proliferator that regulates adipocyte differentiation and controls the peroxisomal beta-oxidation pathway of fatty acids (Małodobra-Mazur et al., 2022). Tumor necrosis factor (TNF) is mainly secreted by macrophages and can induce insulin resistance through inhibition of Insulin receptor substrate 1 (IRS1), tyrosine phosphorylation and G kinase-anchoring protein 1 (GKAP42) degradation in adipocytes (Ando et al., 2015). Taken together, we provide preliminary evidence that the potential mechanism in AO-induced liver injury may mediate lipid metabolism *via* network toxicology.

PPI network analysis showed that IGF, AKT1, EGFR and MAPK1 were the main targets of AO in the treatment of PIH; the molecular docking and molecular dynamics simulation results also exhibited good binding affinity and stability. Besides, KEGG pathway enrichment analysis showed that MAPK and PI3K-AKT signal pathways were significantly enriched. Insulin-like growth factor I (IGF1) can bind to the alpha subunit of IGF1 receptor and modulate the tyrosine kinase activity to initiate the down-stream signaling events activation of PI3K-AKT and Ras-MAPK pathways (Mohindra et al., 2022). It has been reported that IGF1 is differentially expressed between PIH and normal pregnancy newborns, and IGF1 could stimulate extra-villous trophoblast (EVT) cell migration and invasion, given that insufficient invasion into the uterine endometrium may be the crucial reason for PIH (Seedorf et al., 2020). Epidermal growth factor receptor (EGFR) and RAC-alpha serine/threonine-protein kinase (AKT1) are important hub genes that regulate a series of biological functions, including angiogenesis (Zhang et al., 2022b). Mitogen-activated protein kinase 1 (MAPK1) has been associated with the transduction of endothelial inflammatory response and inflammatory factor expression action (Galganska et al., 2021). Wang Z et al. indicated that overexpressing miR-106 and downregulating the MAPK signaling cascade could attenuate oxidative stress injury and inflammatory response in the liver of mice with PIH (Wang et al., 2019). Li X et al. found significant Cx43 protein overexpression on human umbilical vein endothelial cells (HUVECs) in PIH patients, which may lead to monocyte-endothelial adhesion increase by PI3K-AKT signaling pathway activation and upregulate the downstream genes of VCAM-1 and ICAM-1 (Li et al., 2019c).

In conclusion, we found that the main compounds of AO in treating PIH are triterpenoids, especially Alisol B, Alisol C and their derivatives, which target IGF, AKT1, EGFR and MAPK1 and mediate the MAPK and PI3K-AKT signal pathways to produce diuretic and immunomodulatory activities and regulate the function of endothelial cells, anti-vascular inflammation and oxidative stress. In addition, our study preliminarily indicated that the toxic compounds in AO were mainly emodin and small amounts of fatty acids, which may produce hepato-nephrotoxicity by interfering with lipid metabolism. Furthermore, our study suggests that an optimal therapeutic effect against PIH may be achieved by increasing the triterpenoid content and reducing toxic compounds through appropriate methods. However, some shortcomings in the present study affect the robustness of our findings to some extent. First, our study was based on the identified and main bioactive compounds of AO, which may have some selection bias. Second, and crucially, further *in vivo* and *in vitro* basic research is needed to confirm and extend our conclusions. And this was exactly what we need to do next, to verify the mechanisms of AO core compounds with therapeutic efficacy in PIH and hepato-nephrotoxicity in future research.

## Conclusion

This study was based on the bioinformatics analysis of network pharmacology, network toxicology, molecular docking and molecular dynamics simulation. The finding shows that the triterpenoids were the main therapeutic compounds and emodin was the main toxic compound of Alisma Orientale. And also illustrated the potential mechanism underlying the therapeutic effects of AO against PIH and AO induced hepato-nephrotoxicity. In short, our research could guide directions for subsequent basic experimental verification and provide points of view for enhance efficacy and reduce toxicity in clinical.

## Data Availability

The datasets presented in this study can be found in online repositories. The names of the repository/repositories and accession number(s) can be found in the article/Supplementary Material.
