# Supplementary figures and images for: Exploring the mechanism of Alisma orientale for the treatment of pregnancy induced hypertension and potential hepato-nephrotoxicity by using network pharmacology, network toxicology, molecular docking and molecular dynamics simulation
Source: Front Pharmacol. 2022 Nov 15;13:1027112. doi: 10.3389/fphar.2022.1027112 (PMC9705790; doi:10.3389/fphar.2022.1027112)

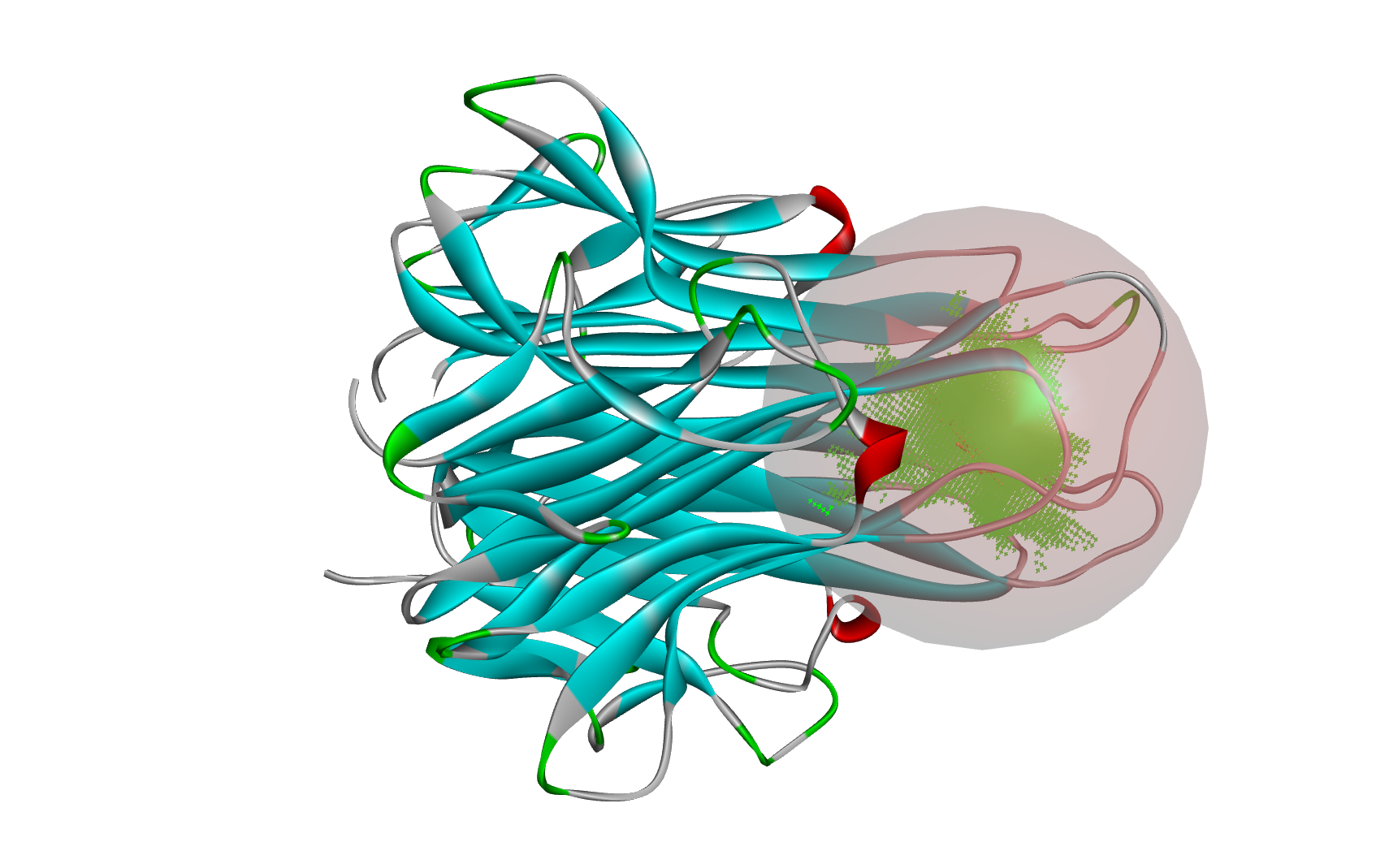

Supplement: Supplementary file 4 [file DataSheet8.ZIP › Supplement Materials/Molecular Docking/Toxic/2TNF 1.png]

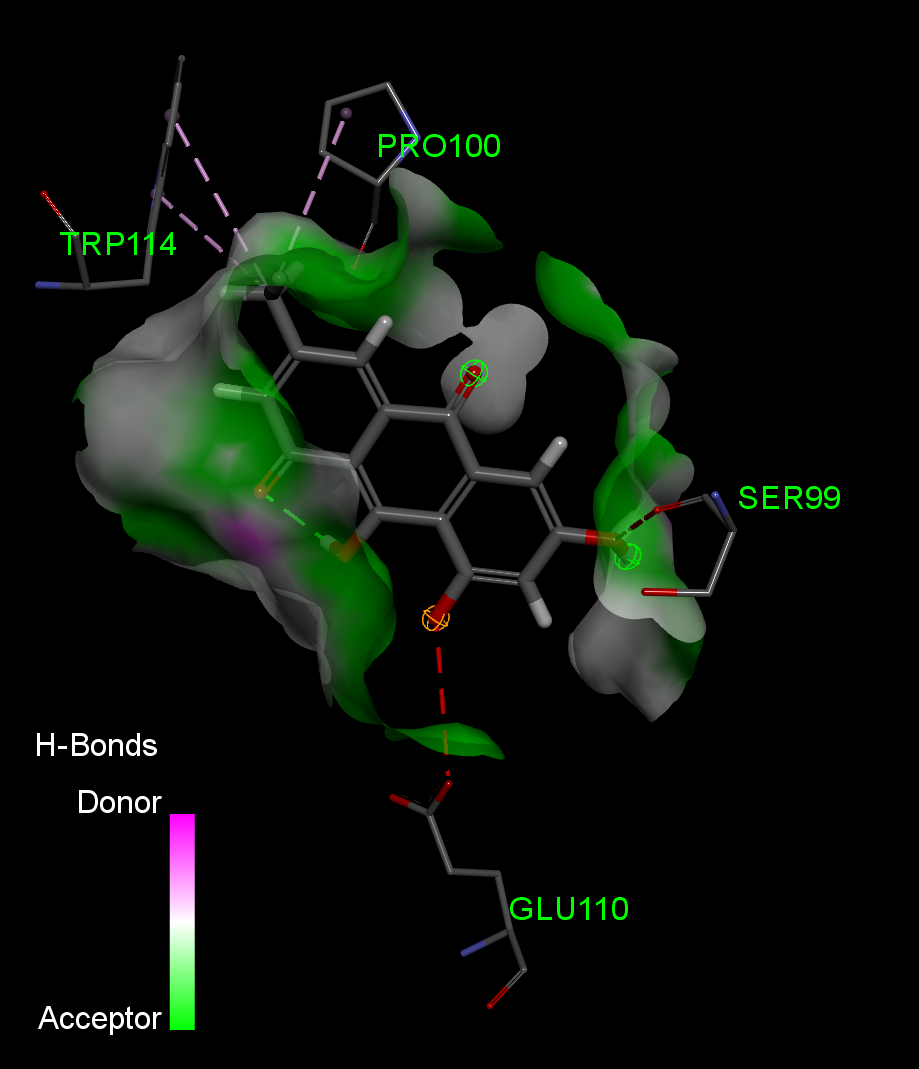

Supplement: Supplementary file 4 [file DataSheet8.ZIP › Supplement Materials/Molecular Docking/Toxic/2TNF 2.png]

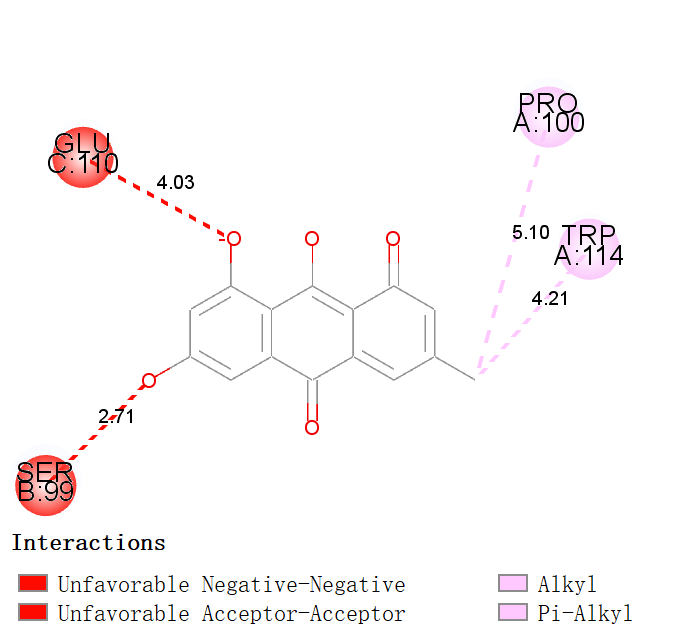

Supplement: Supplementary file 4 [file DataSheet8.ZIP › Supplement Materials/Molecular Docking/Toxic/2TNF 3.png]

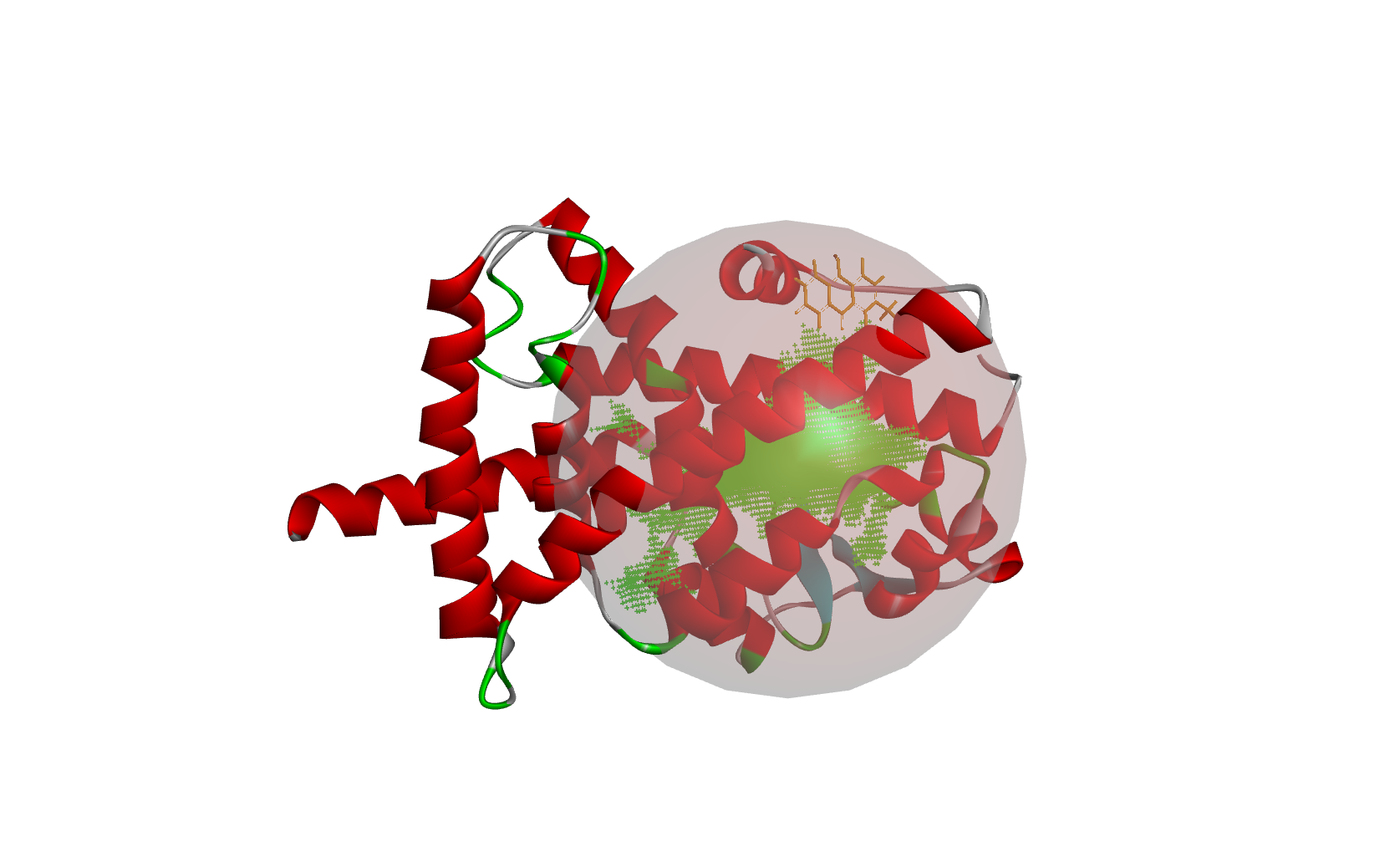

Supplement: Supplementary file 4 [file DataSheet8.ZIP › Supplement Materials/Molecular Docking/Toxic/6MS7 1.png]

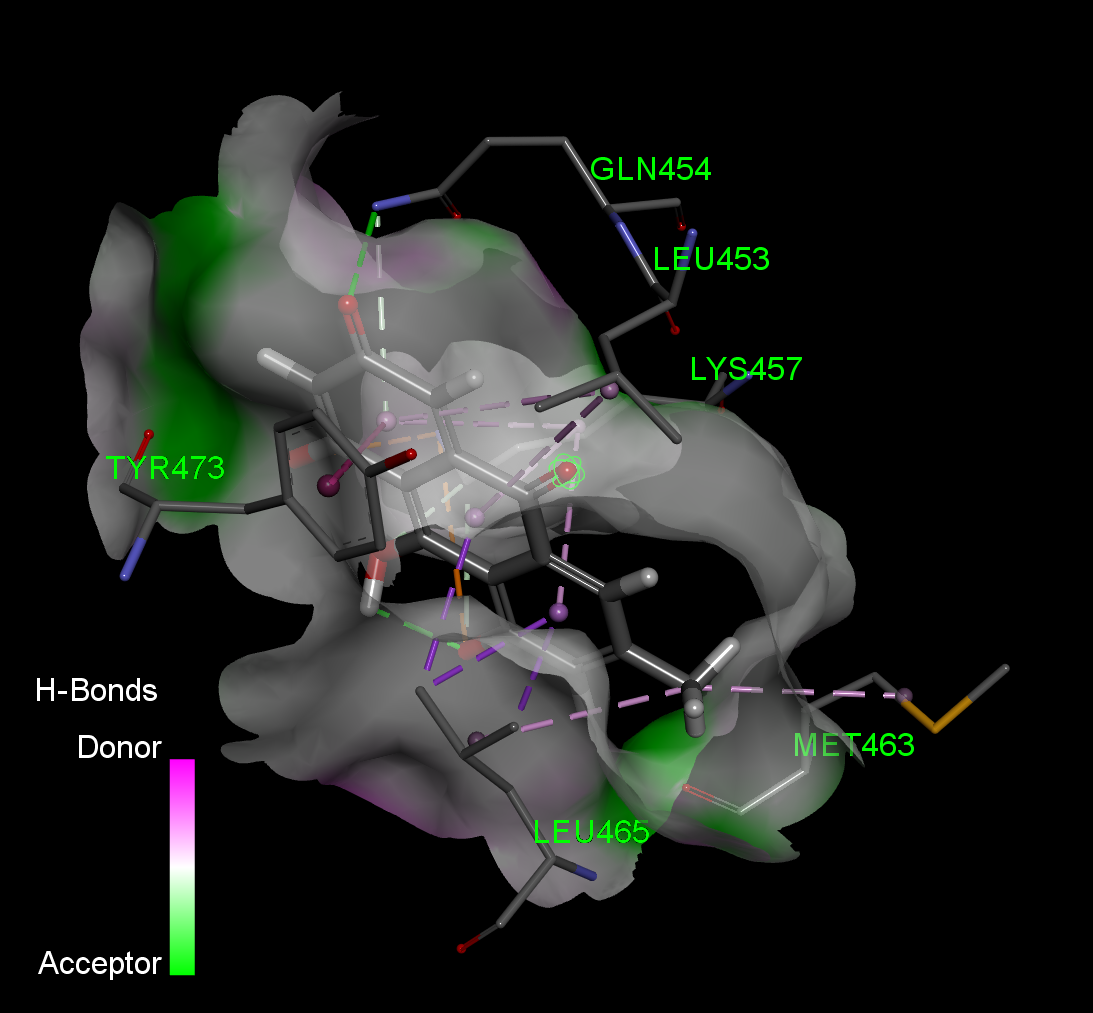

Supplement: Supplementary file 4 [file DataSheet8.ZIP › Supplement Materials/Molecular Docking/Toxic/6MS7 2.png]

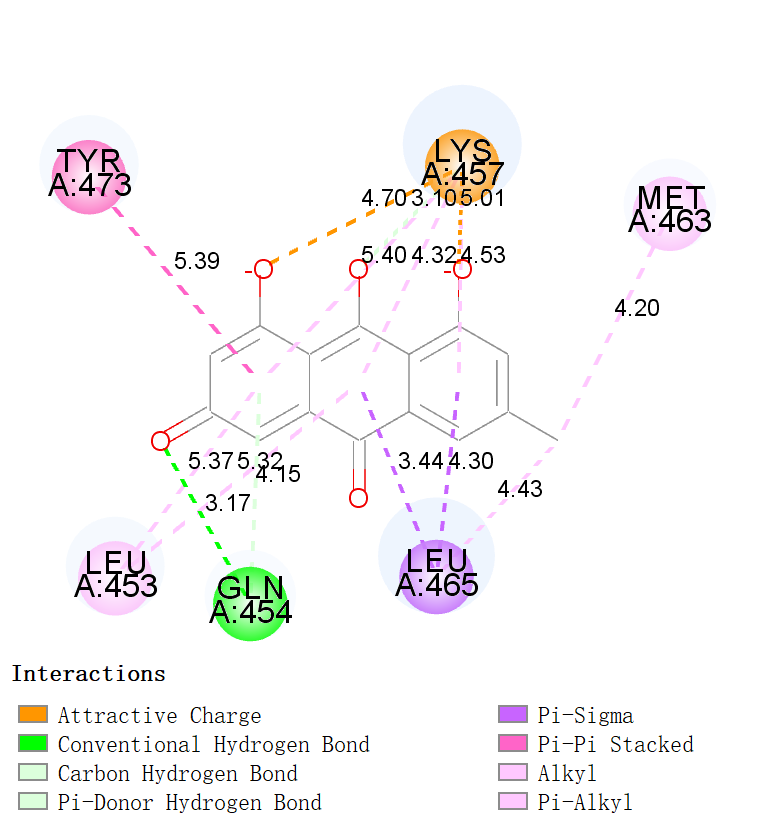

Supplement: Supplementary file 4 [file DataSheet8.ZIP › Supplement Materials/Molecular Docking/Toxic/6MS7 3.png]

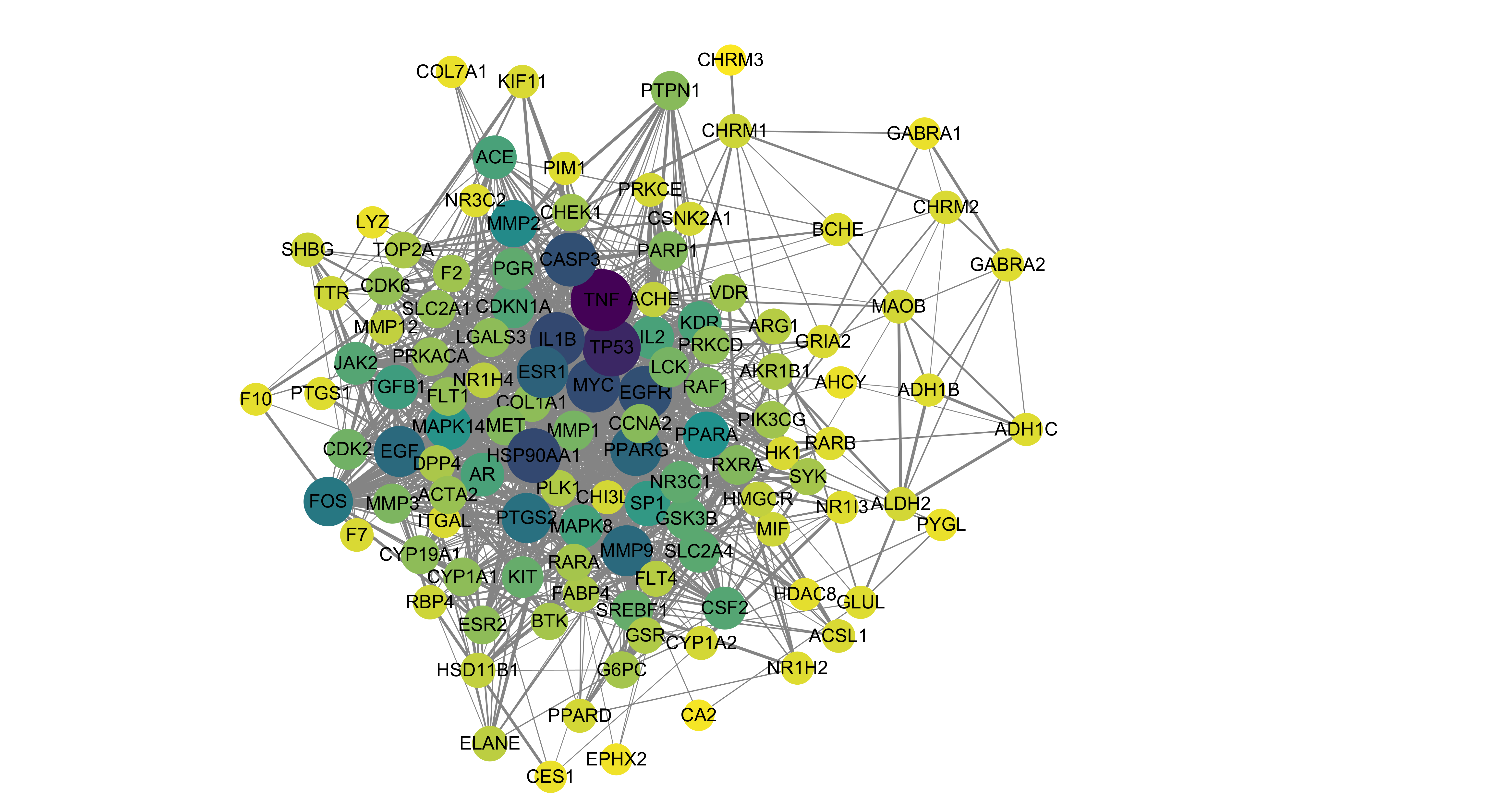

Supplement: Supplementary file 4 [file DataSheet8.ZIP › Supplement Materials/PPI/Toxic/string_cyto_image.png]

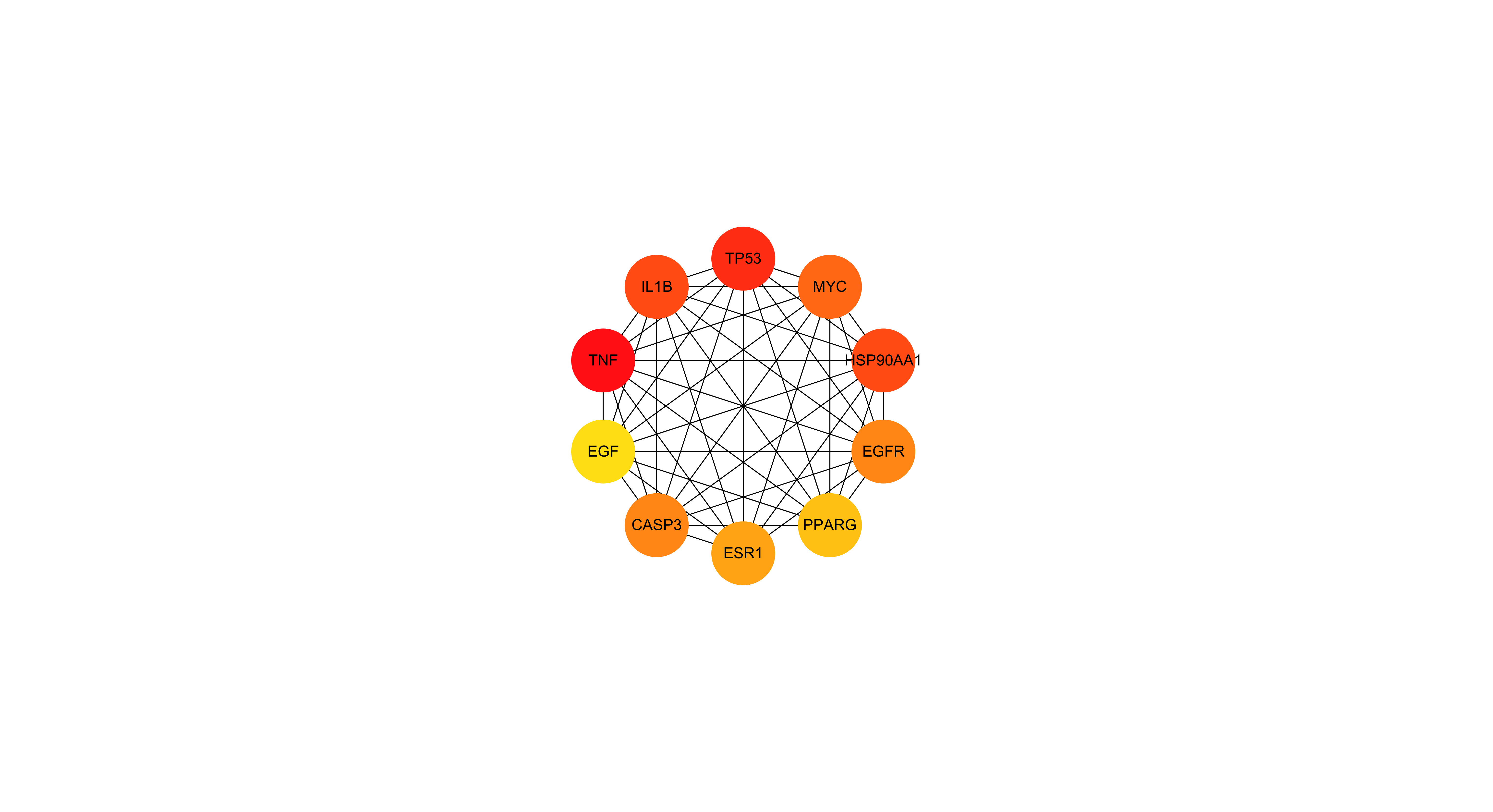

Supplement: Supplementary file 4 [file DataSheet8.ZIP › Supplement Materials/PPI/Toxic/string_hubba-image.png]

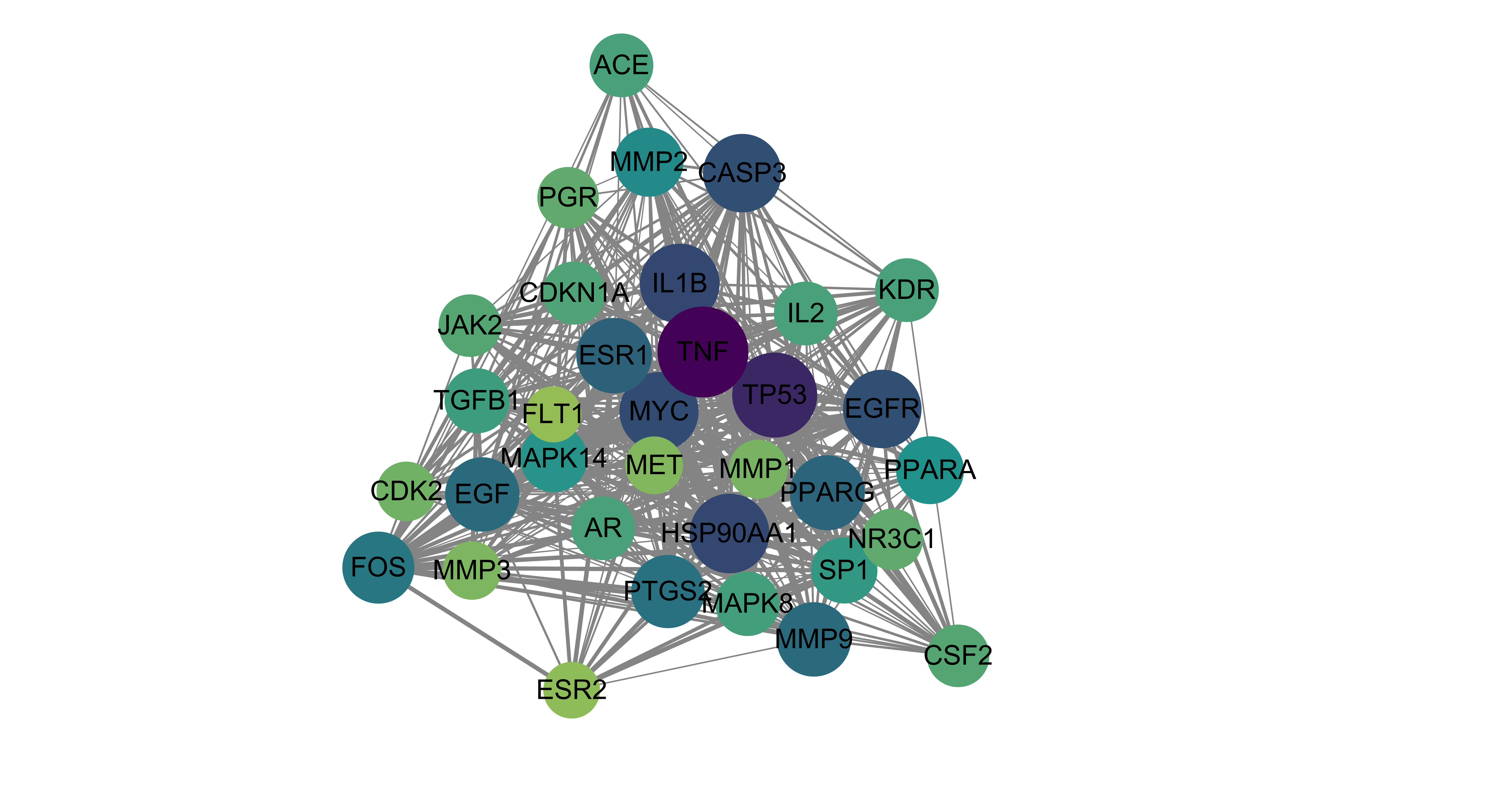

Supplement: Supplementary file 4 [file DataSheet8.ZIP › Supplement Materials/PPI/Toxic/string_mcode_image.png]

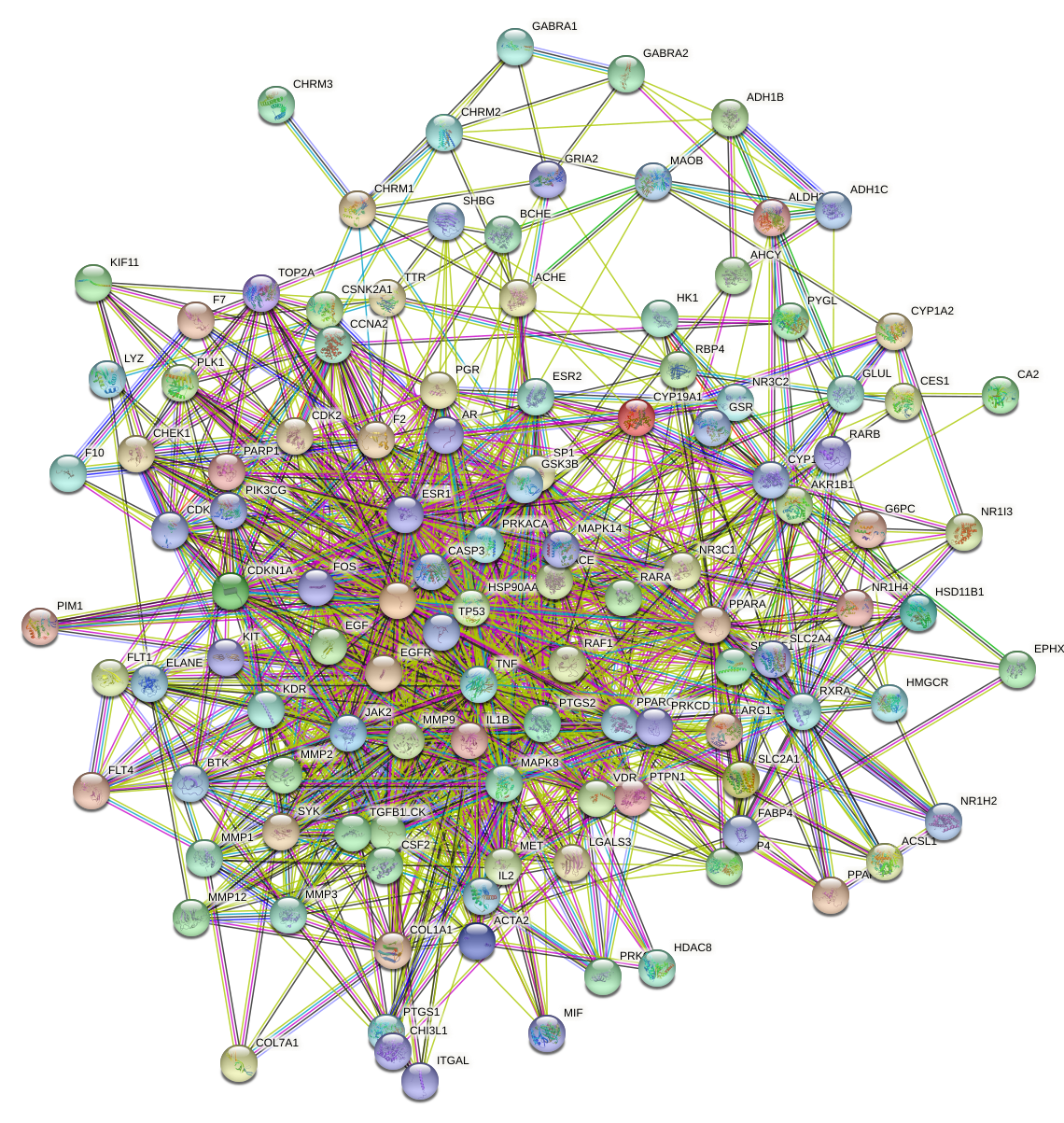

Supplement: Supplementary file 4 [file DataSheet8.ZIP › Supplement Materials/PPI/Toxic/string_normal_image.png]

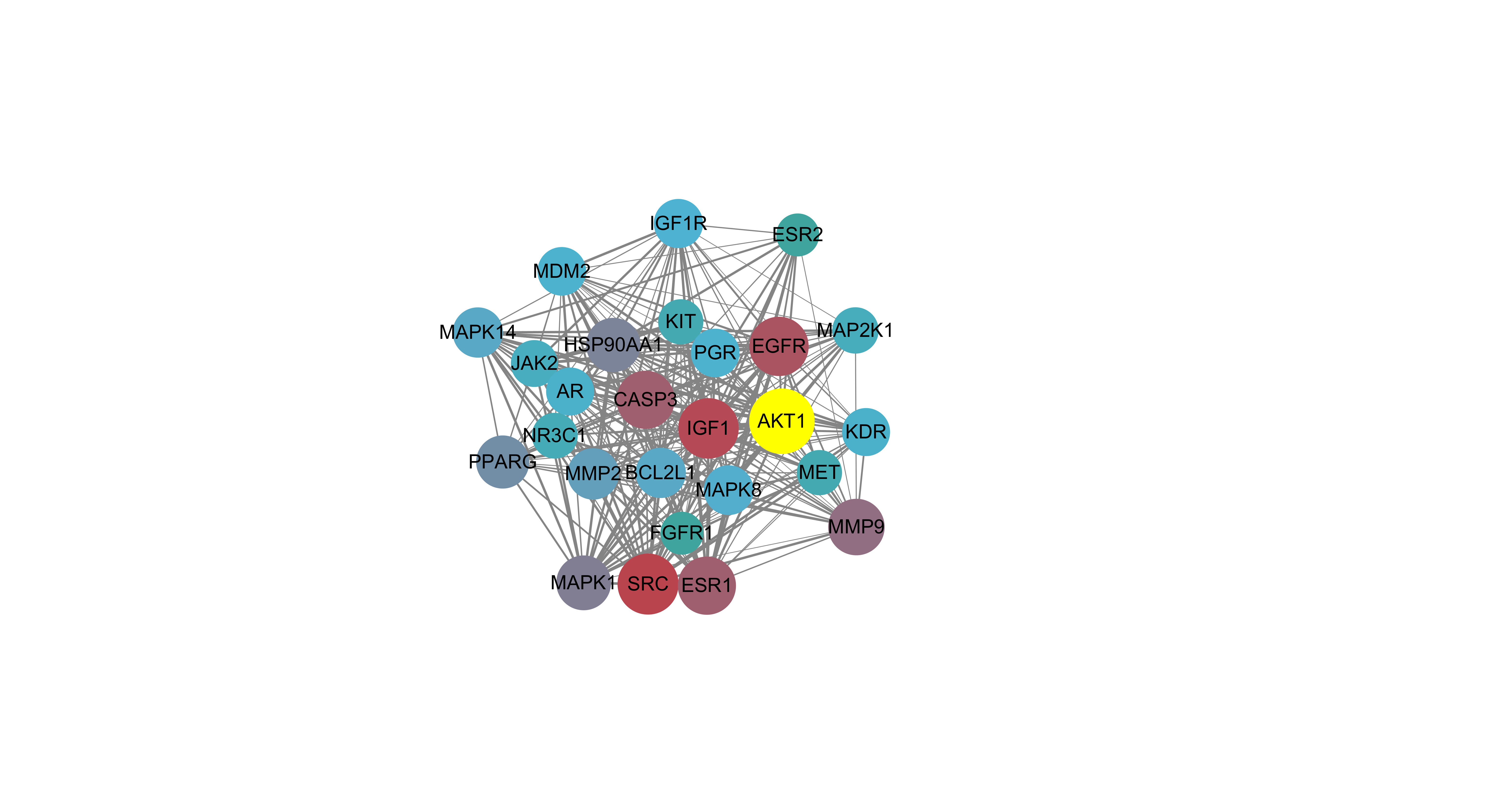

Supplement: Supplementary file 4 [file DataSheet8.ZIP › Supplement Materials/PPI/Treat/string_MCODE_image.png]

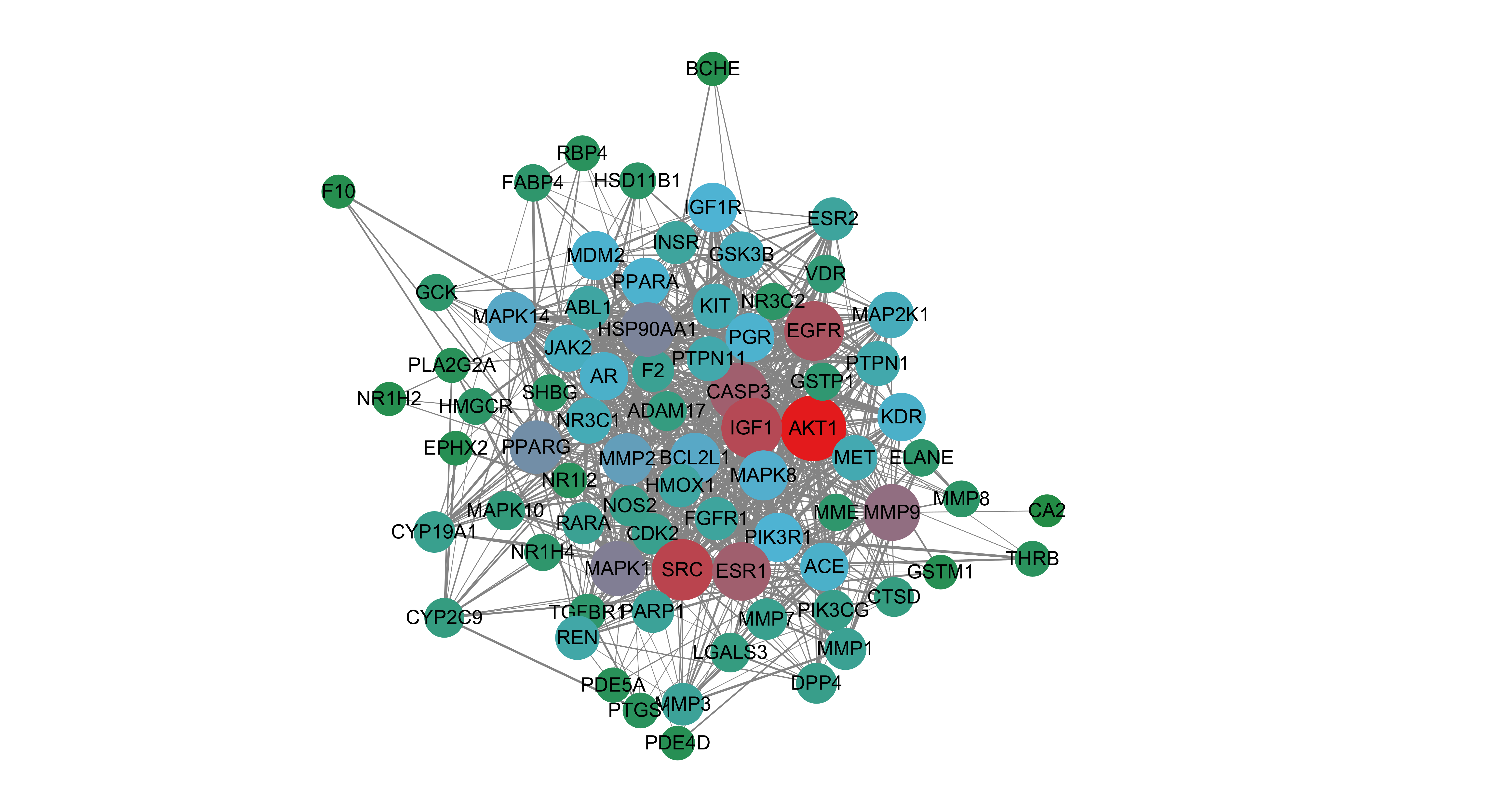

Supplement: Supplementary file 4 [file DataSheet8.ZIP › Supplement Materials/PPI/Treat/string_cyto_image.png]

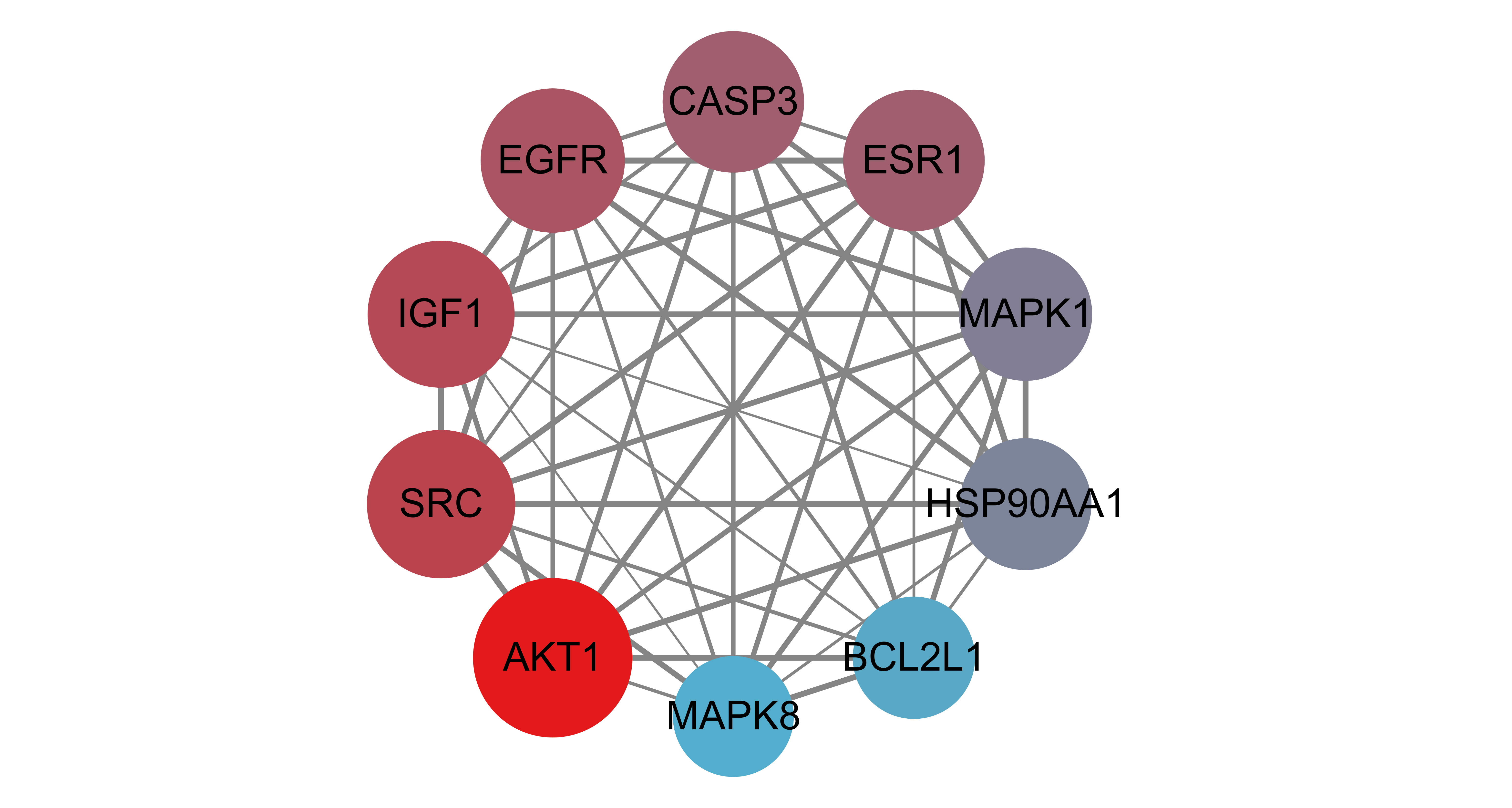

Supplement: Supplementary file 4 [file DataSheet8.ZIP › Supplement Materials/PPI/Treat/string_hubba_image.png]

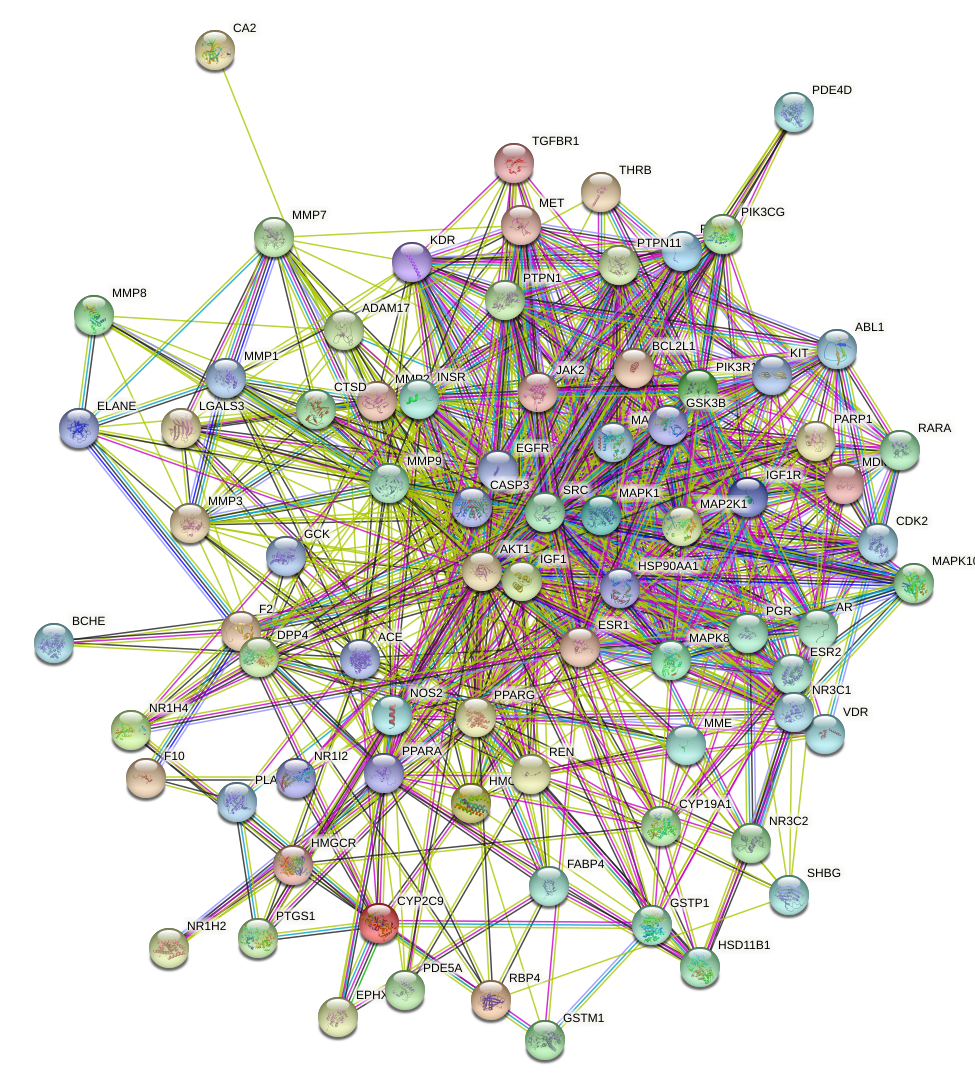

Supplement: Supplementary file 4 [file DataSheet8.ZIP › Supplement Materials/PPI/Treat/string_normal_image.png]

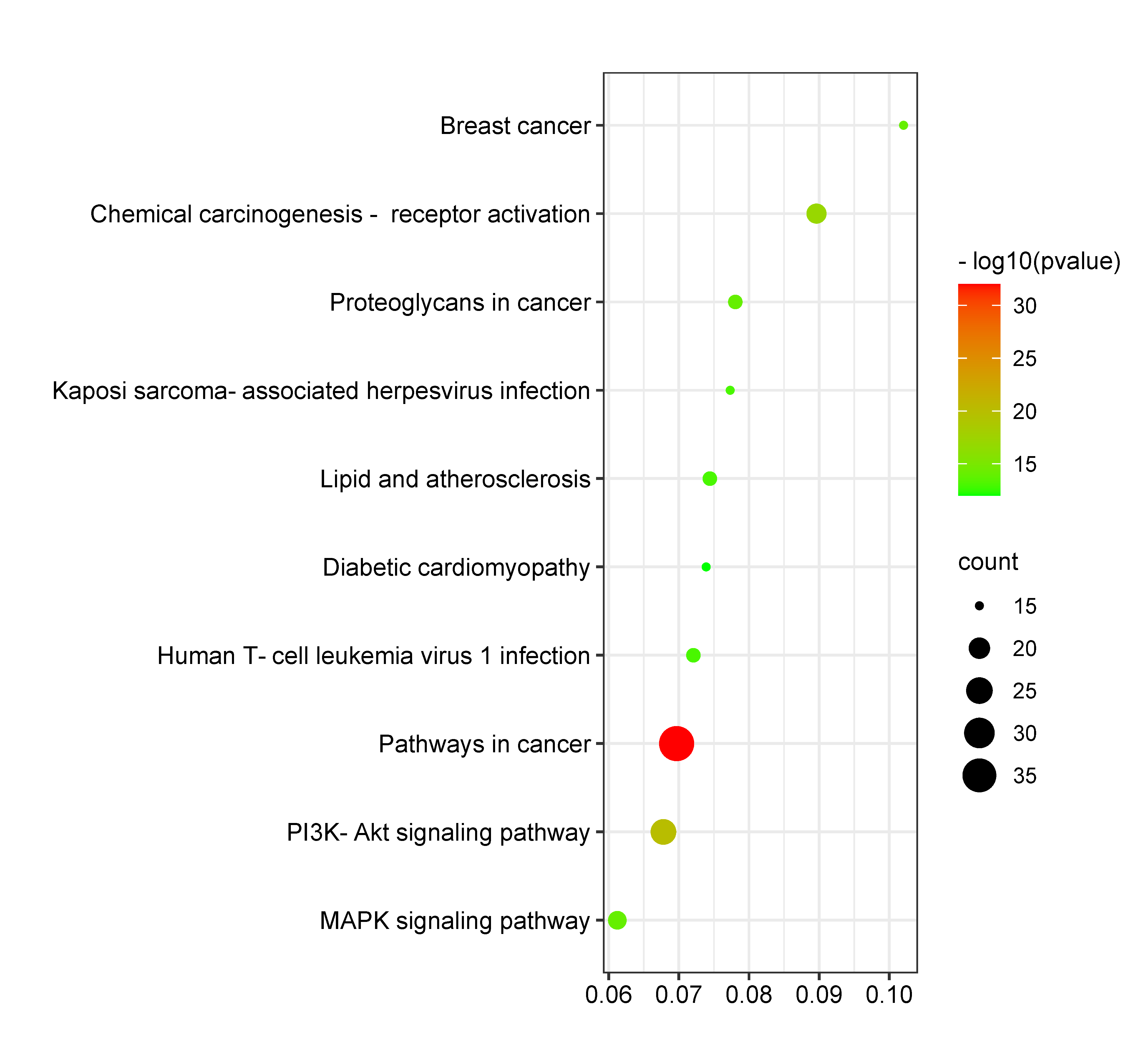

Supplement: Supplementary file 5 [file DataSheet9.ZIP › Revise Nephrotoxicity/307d9a2d45df9160.tiff]

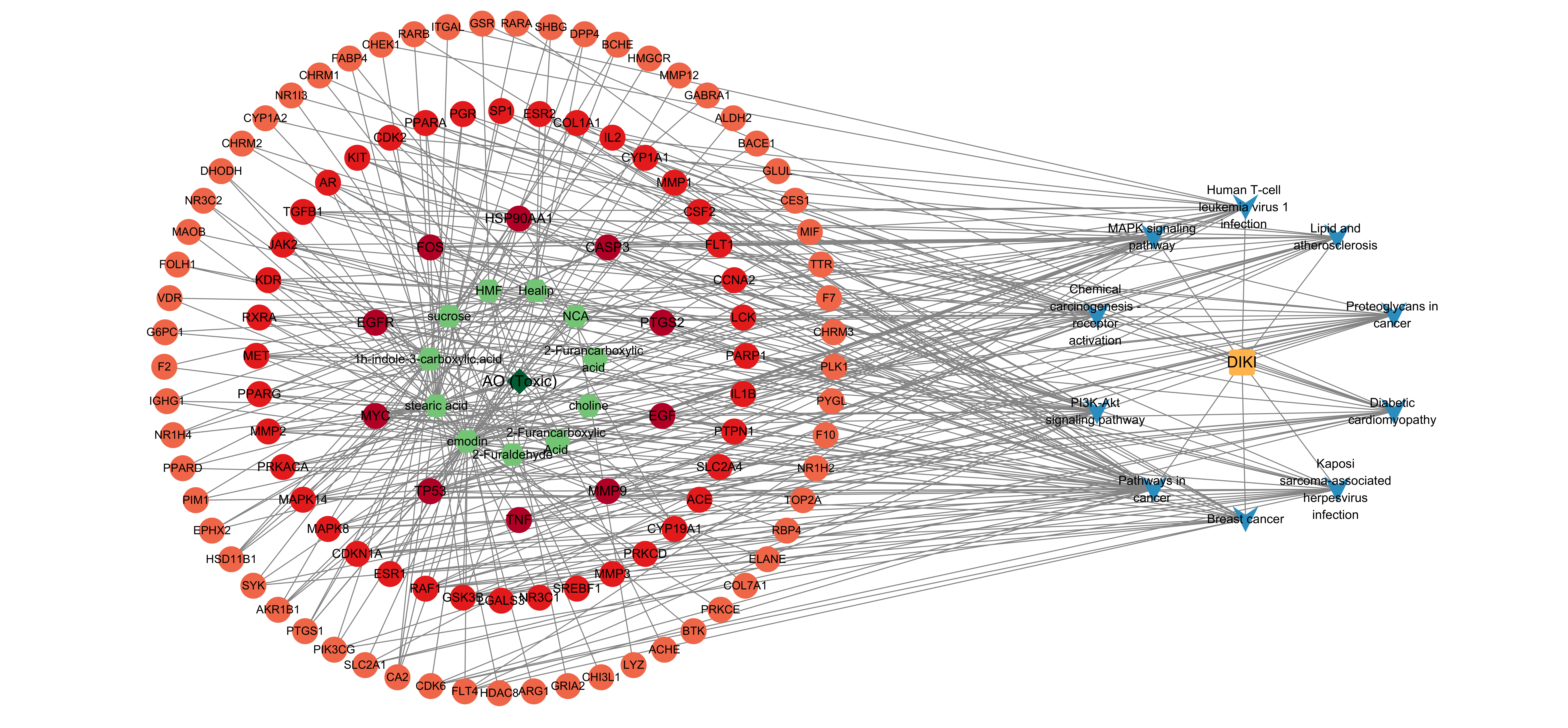

Supplement: Supplementary file 5 [file DataSheet9.ZIP › Revise Nephrotoxicity/D-C-T-P-D.png]

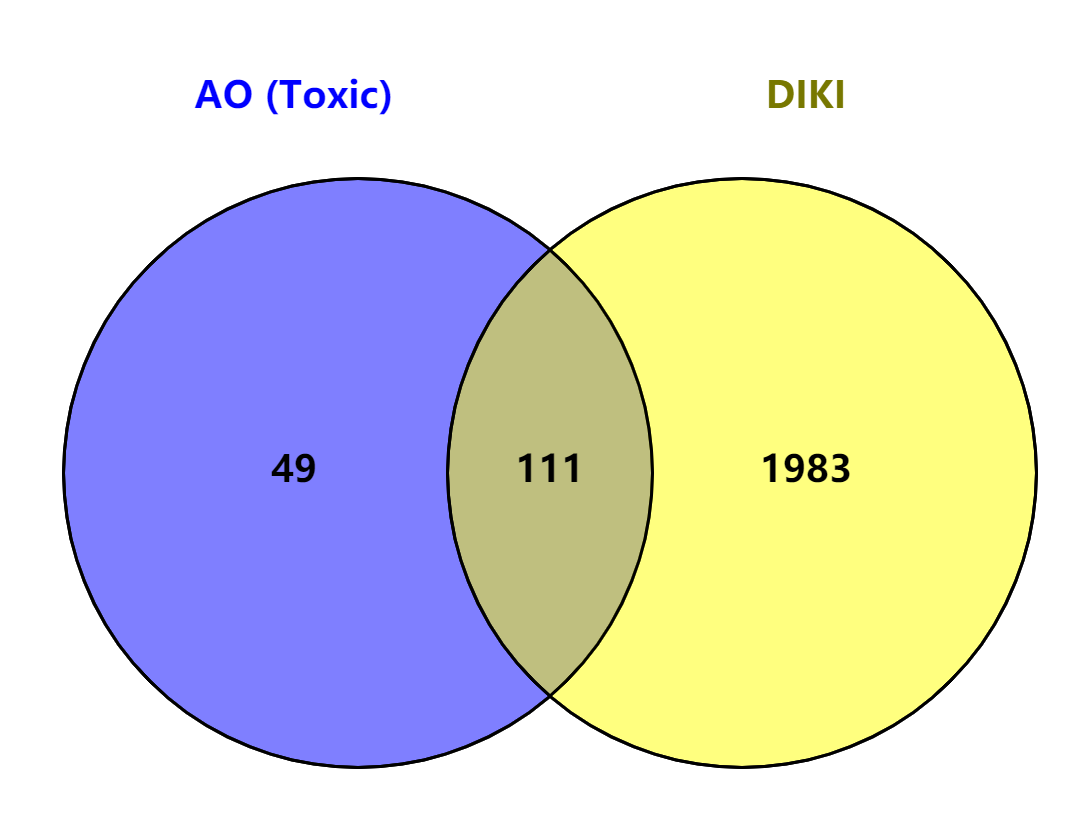

Supplement: Supplementary file 5 [file DataSheet9.ZIP › Revise Nephrotoxicity/Venn.png]

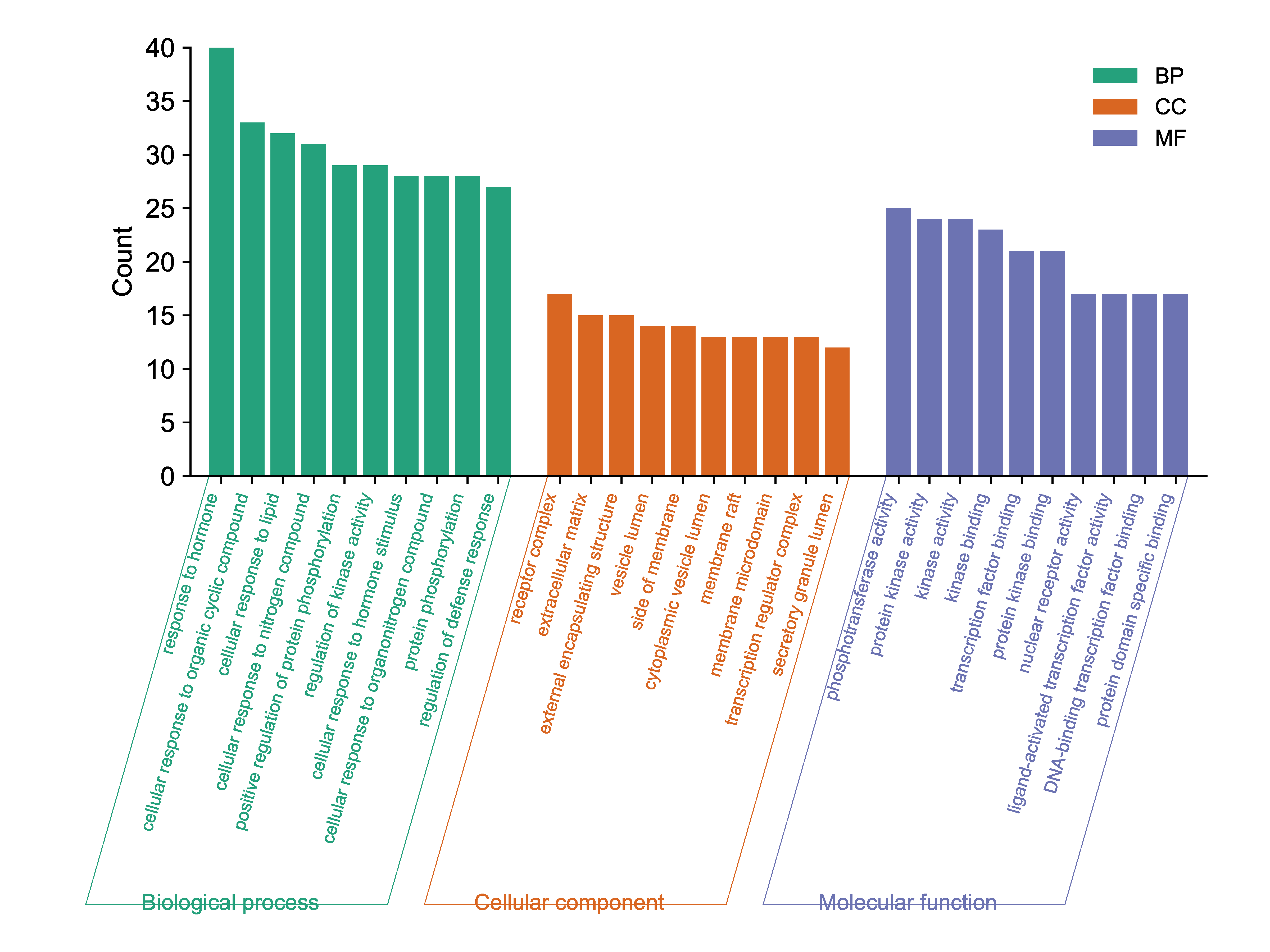

Supplement: Supplementary file 5 [file DataSheet9.ZIP › Revise Nephrotoxicity/b25fe629a1166dc8.tiff]

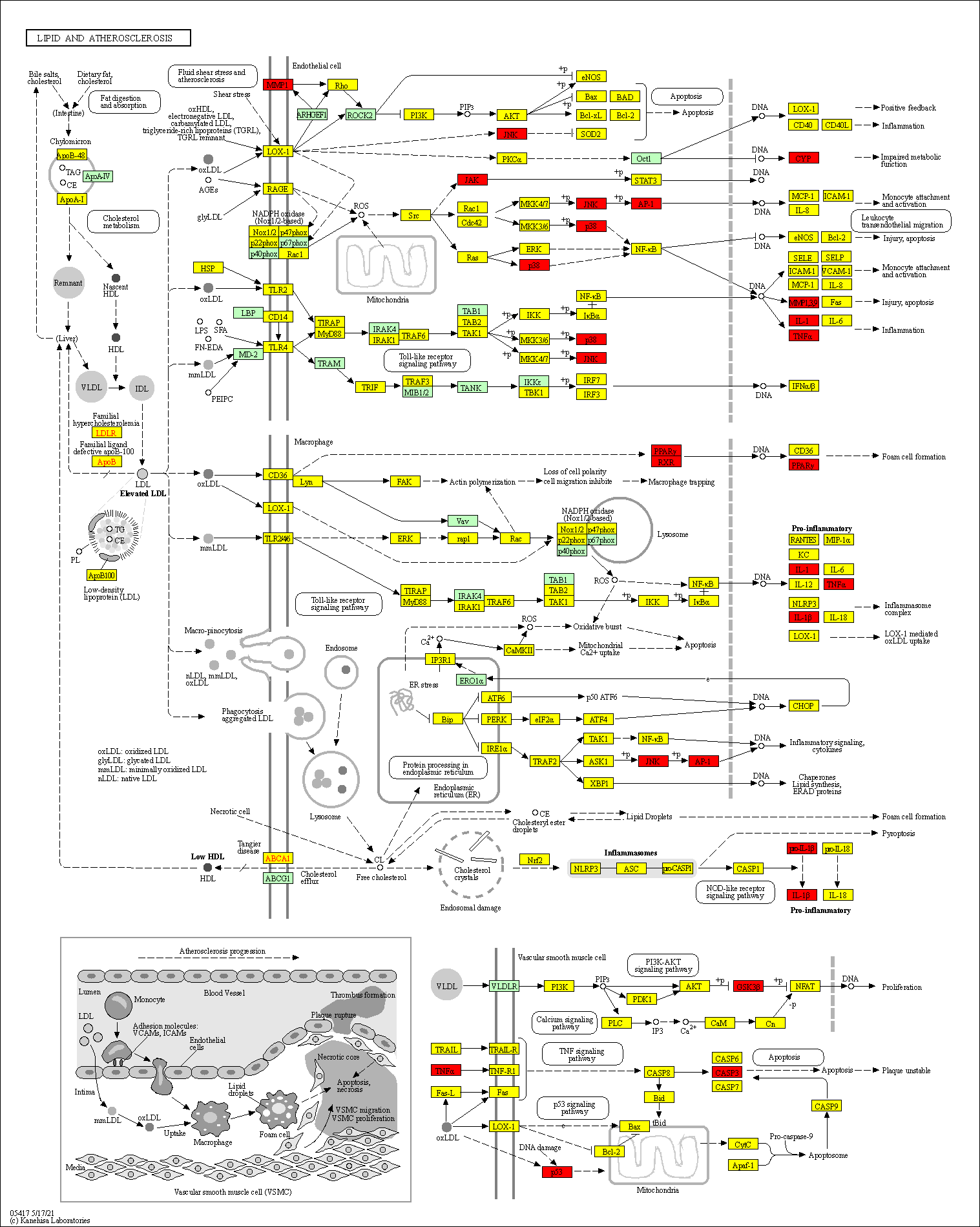

Supplement: Supplementary file 5 [file DataSheet9.ZIP › Revise Nephrotoxicity/hsa05417.png]

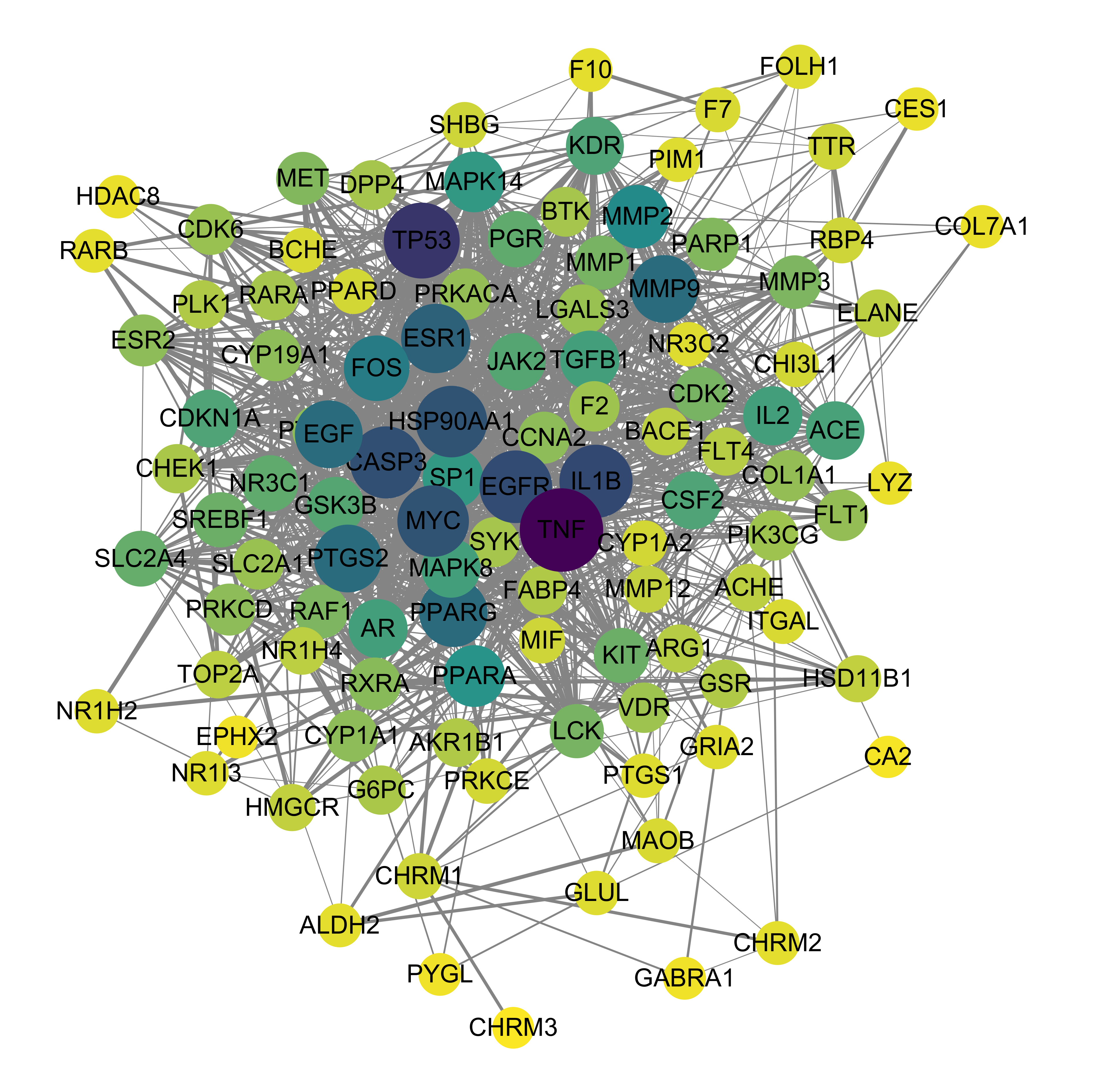

Supplement: Supplementary file 5 [file DataSheet9.ZIP › Revise Nephrotoxicity/string_CYTO_image.png]

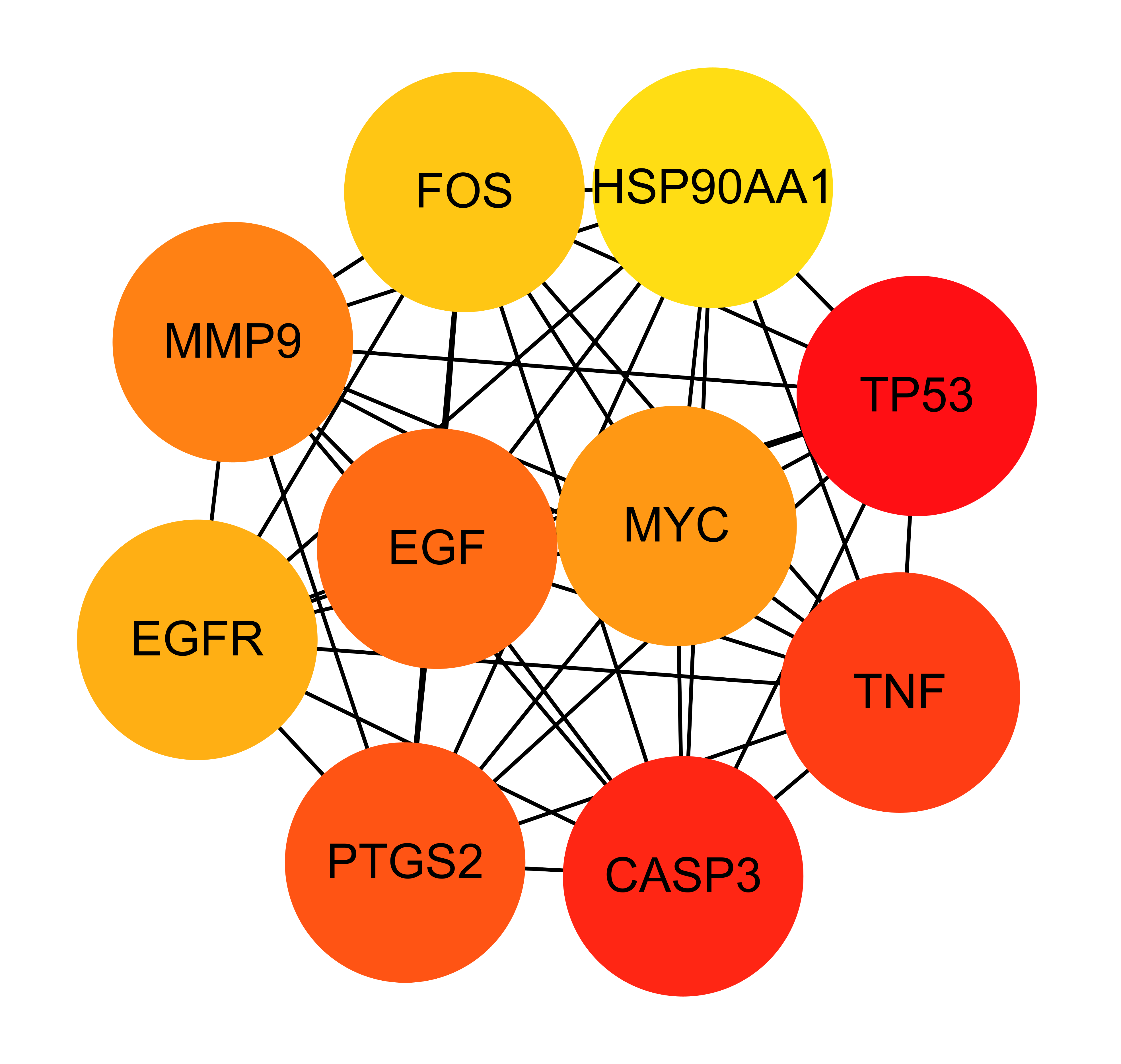

Supplement: Supplementary file 5 [file DataSheet9.ZIP › Revise Nephrotoxicity/string_hubba_image.png]

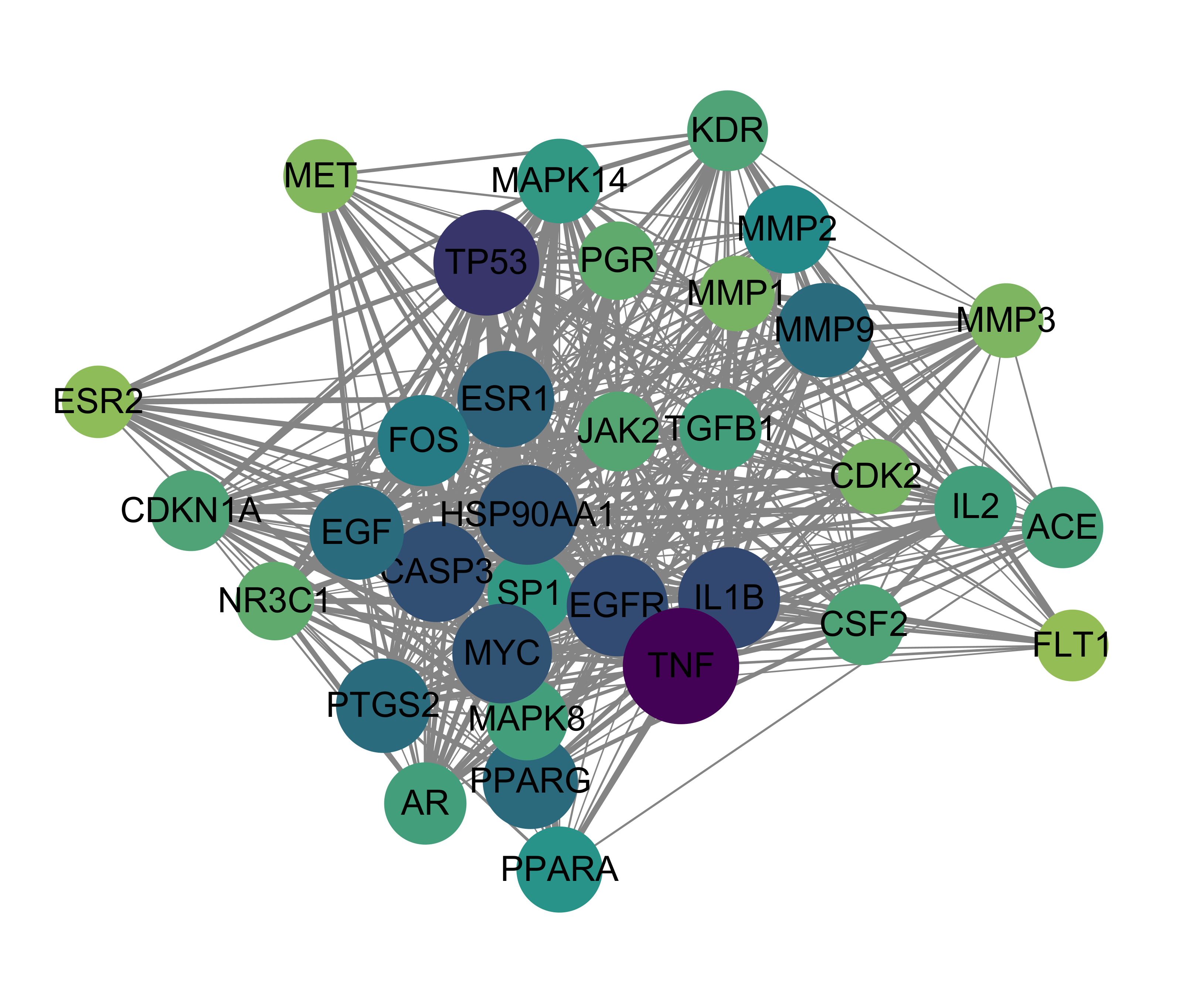

Supplement: Supplementary file 5 [file DataSheet9.ZIP › Revise Nephrotoxicity/string_mcode_image.png]

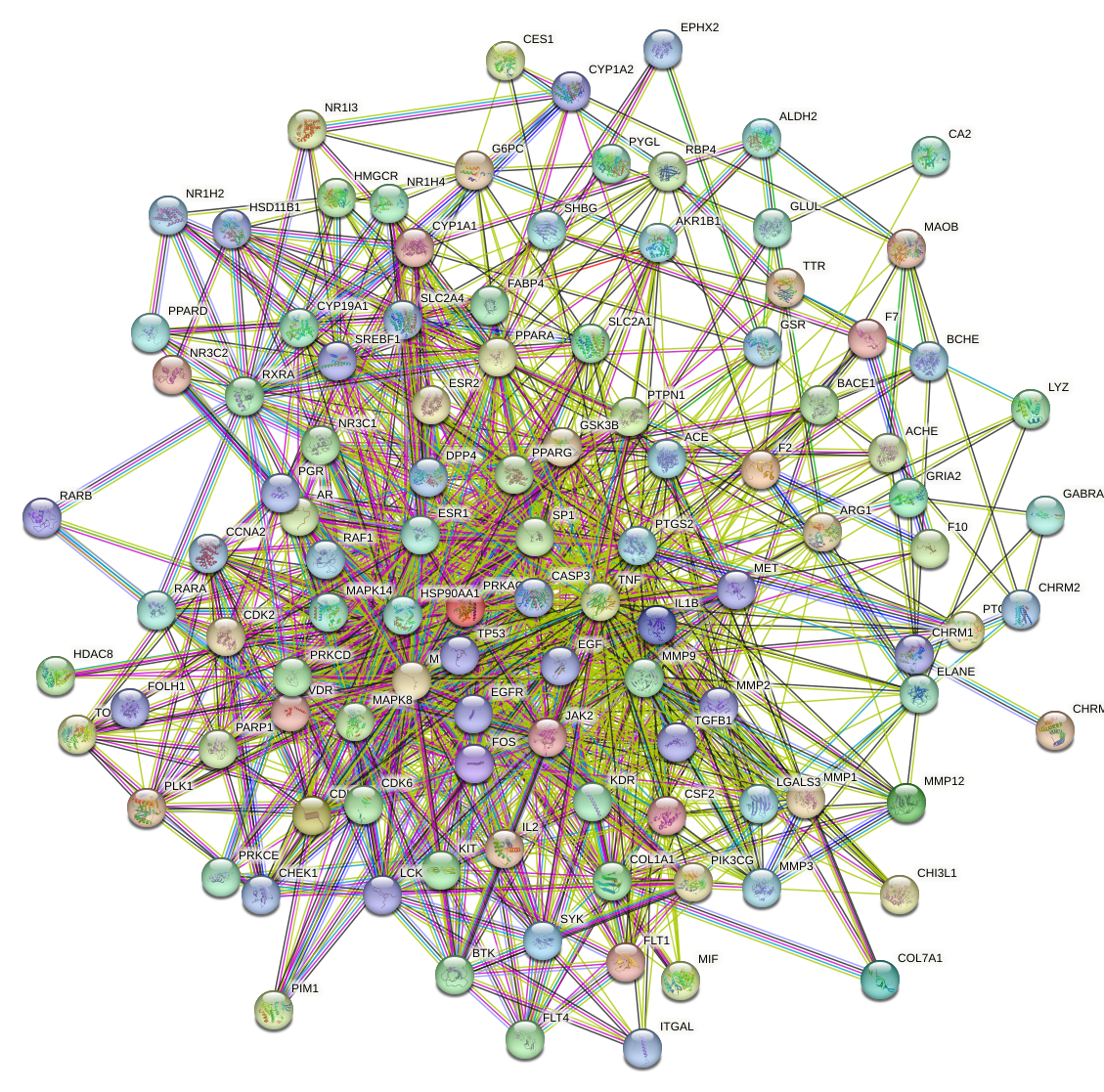

Supplement: Supplementary file 5 [file DataSheet9.ZIP › Revise Nephrotoxicity/string_normal_image.png]

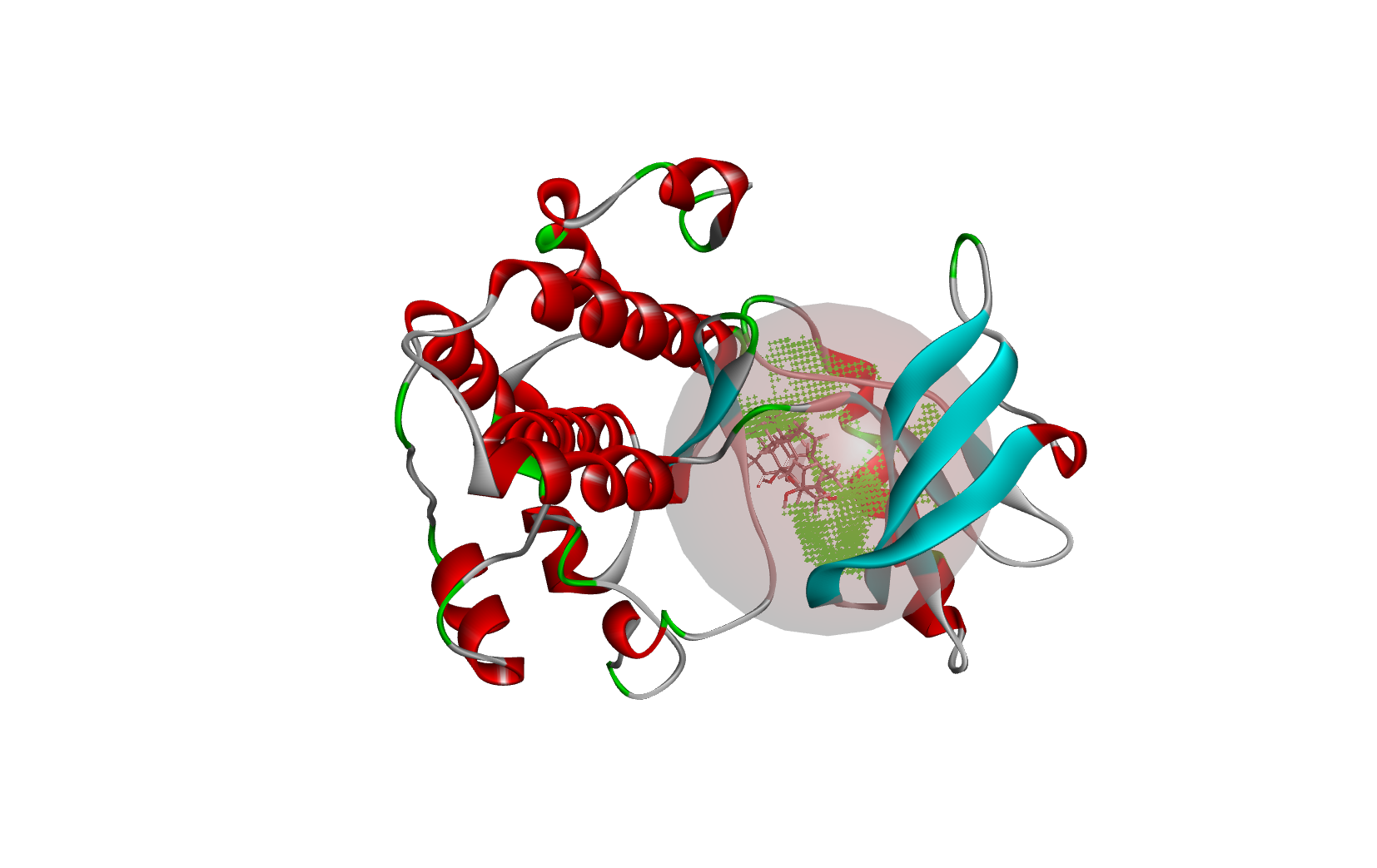

Supplement: Supplementary file 10 [file DataSheet2.zip › Treat/IGF1694 1.png]

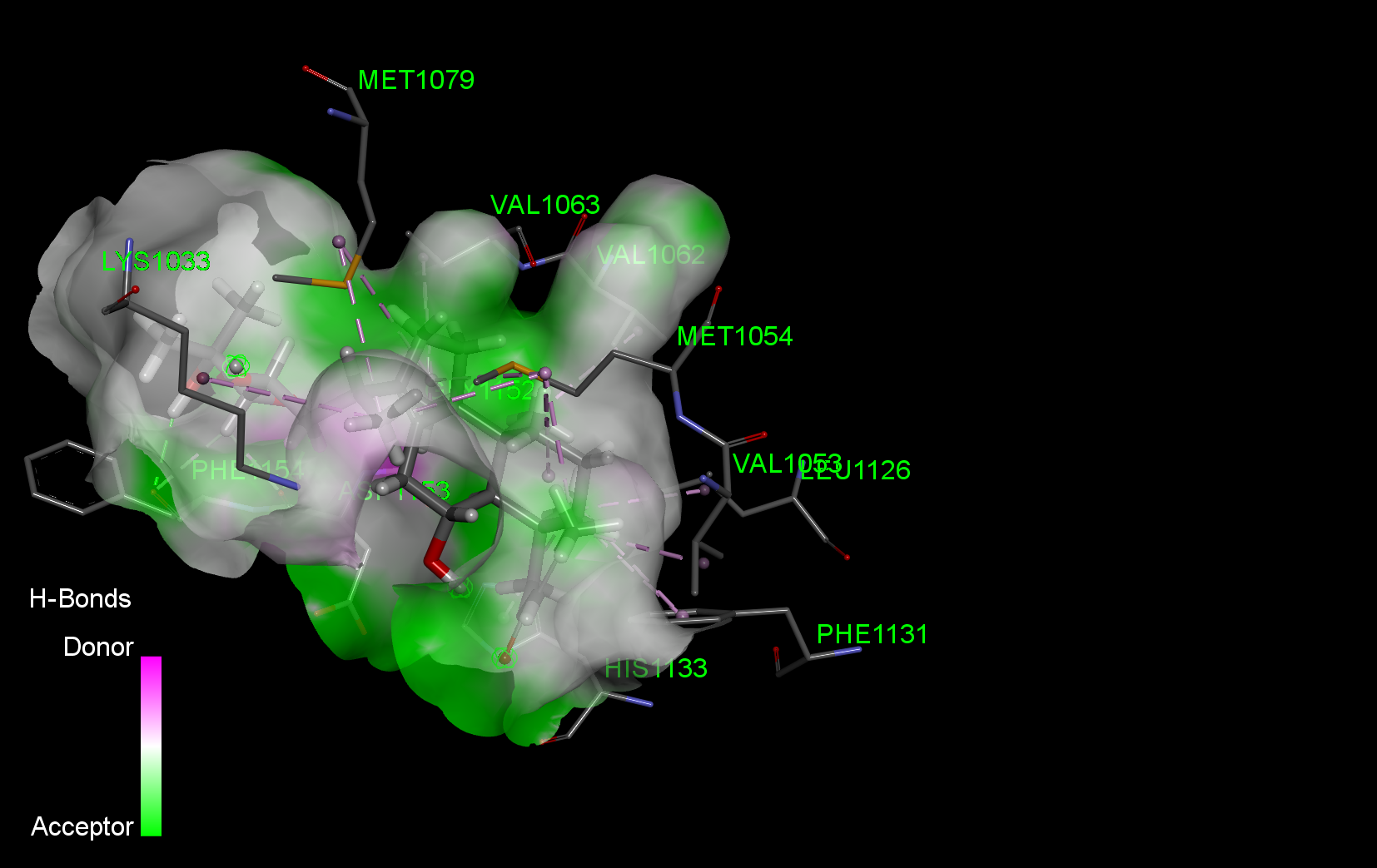

Supplement: Supplementary file 10 [file DataSheet2.zip › Treat/IGF1694 2.png]

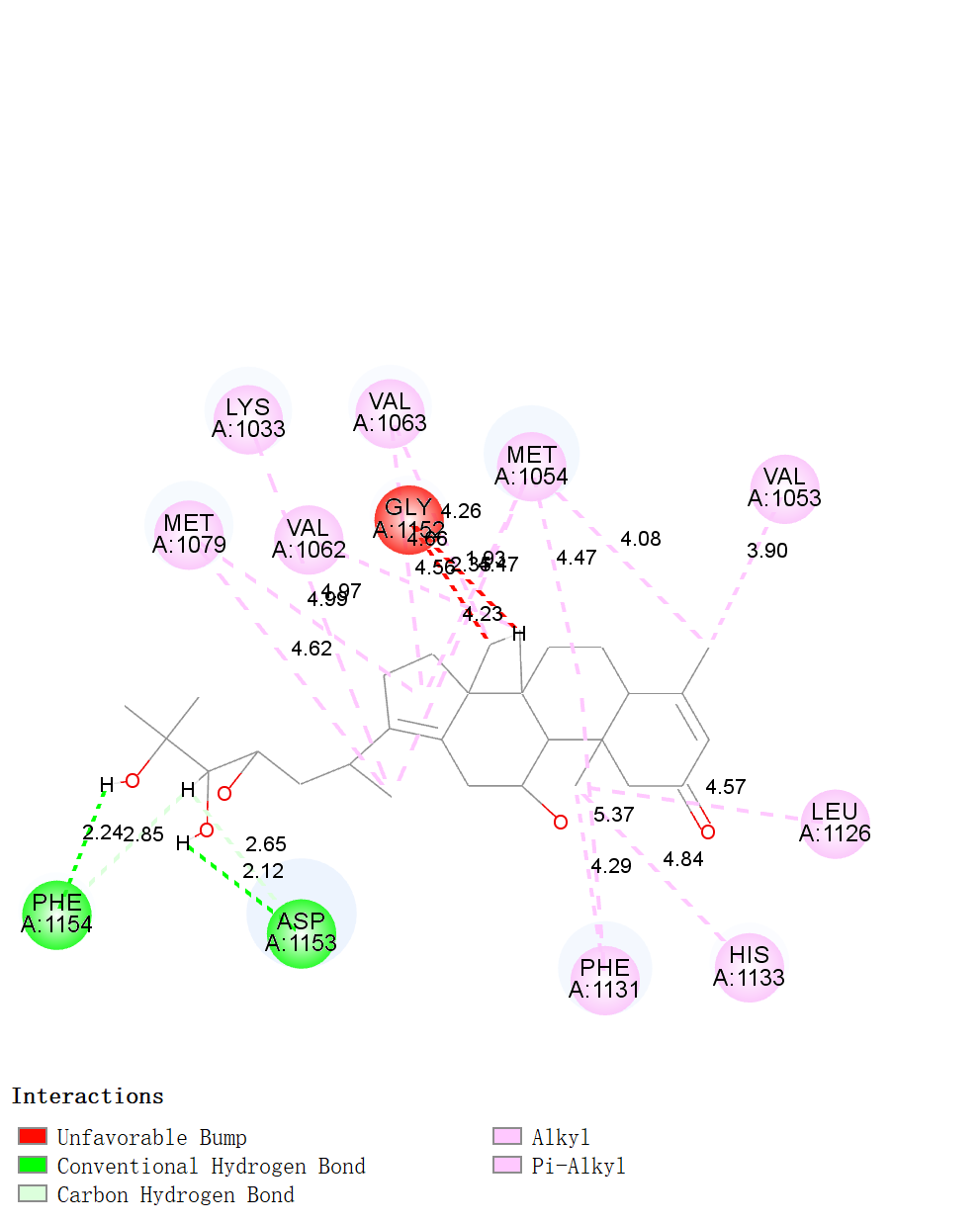

Supplement: Supplementary file 10 [file DataSheet2.zip › Treat/IGF1694 3.png]

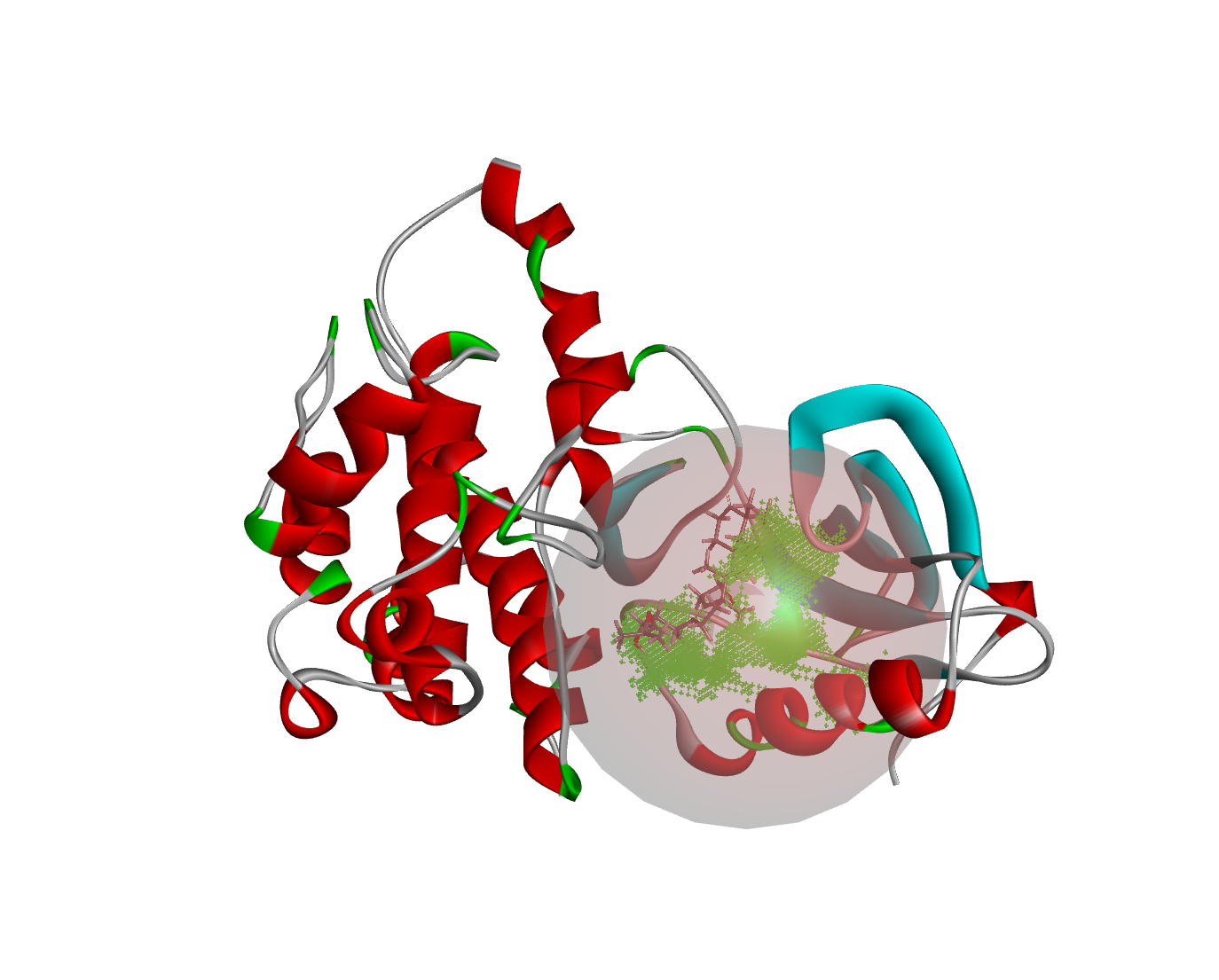

Supplement: Supplementary file 10 [file DataSheet2.zip › Treat/IGF830 1.png]

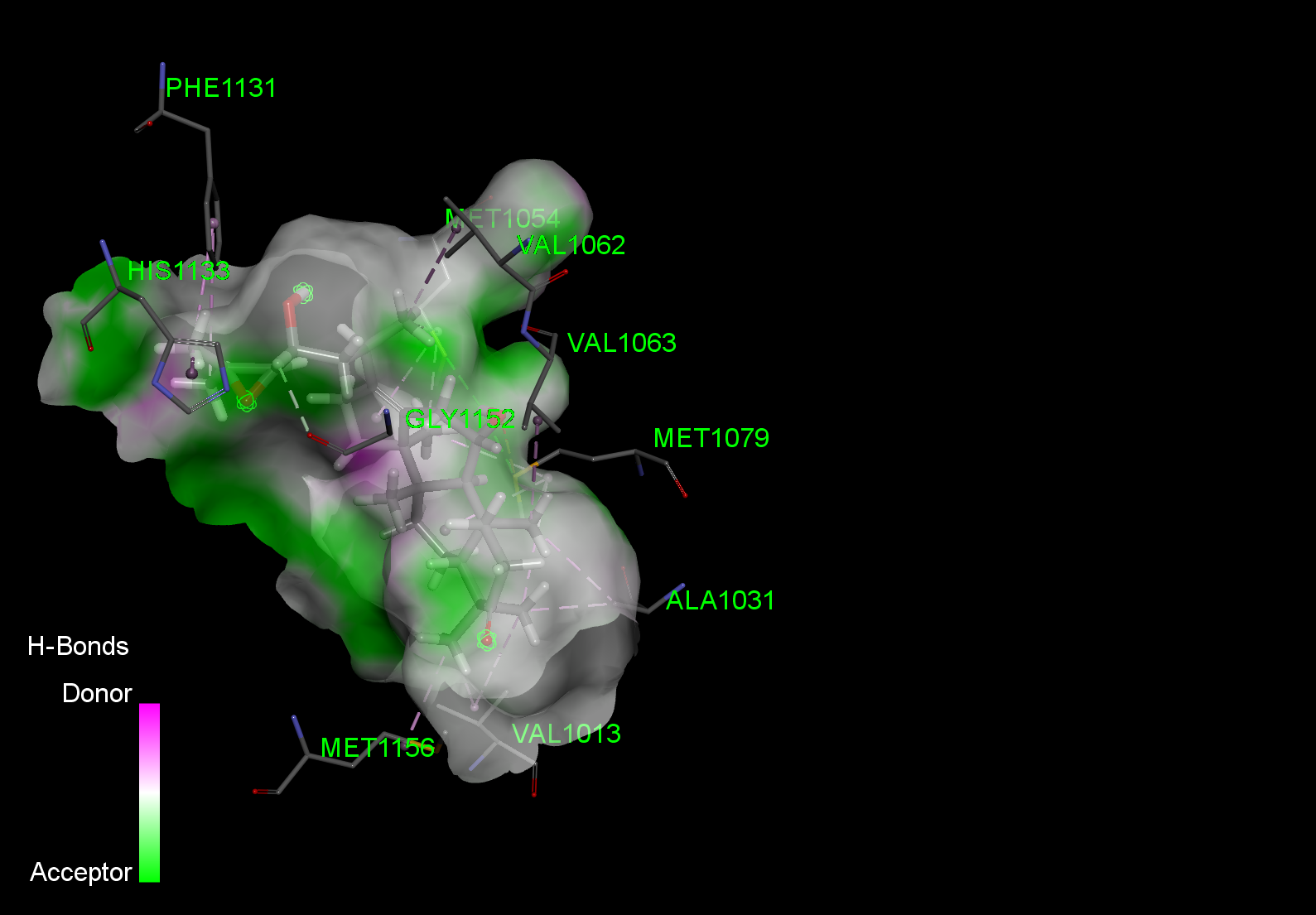

Supplement: Supplementary file 10 [file DataSheet2.zip › Treat/IGF830 2.png]

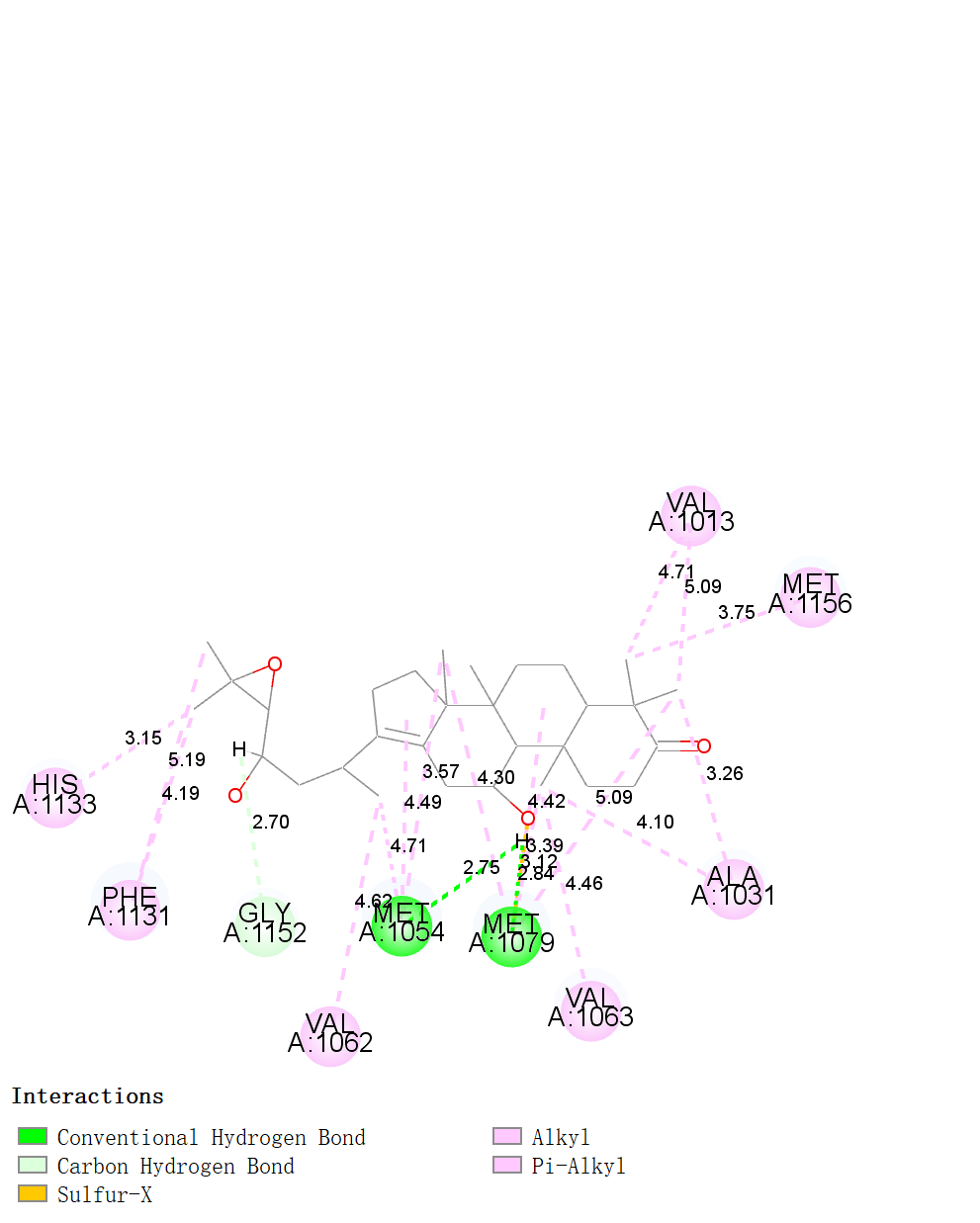

Supplement: Supplementary file 10 [file DataSheet2.zip › Treat/IGF830 3.png]

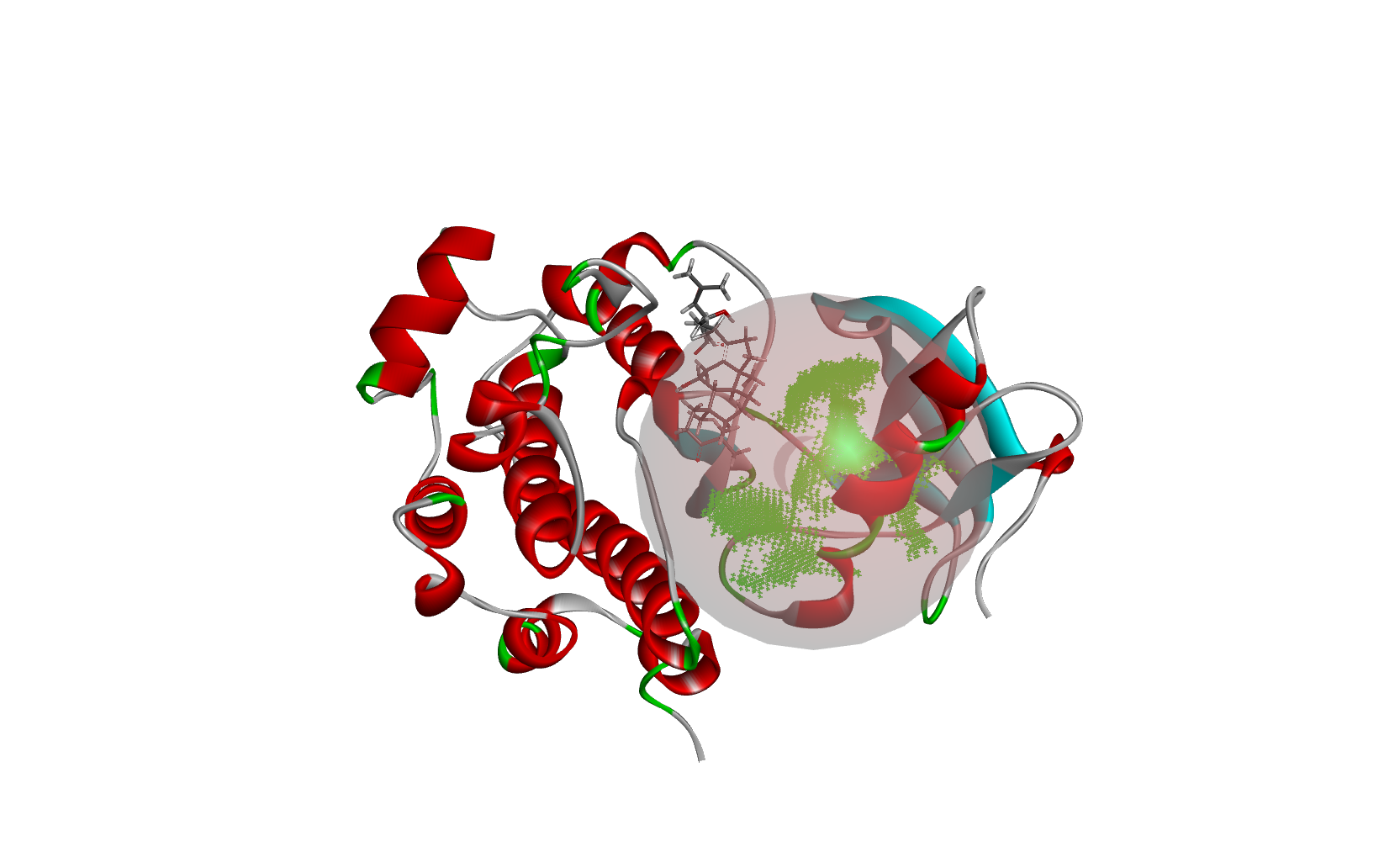

Supplement: Supplementary file 10 [file DataSheet2.zip › Treat/IGFBIG 1.png]

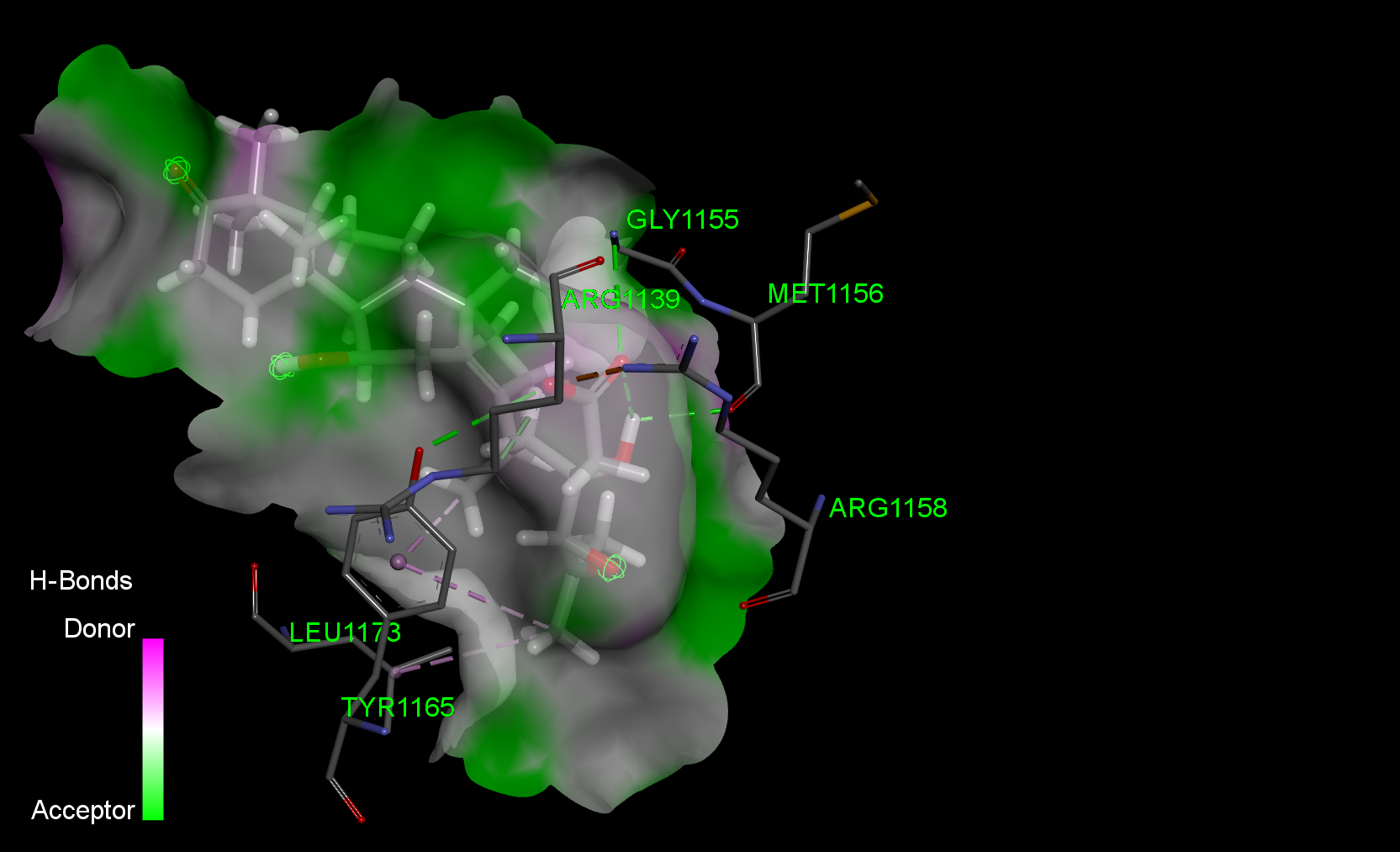

Supplement: Supplementary file 10 [file DataSheet2.zip › Treat/IGFBIG 2.png]

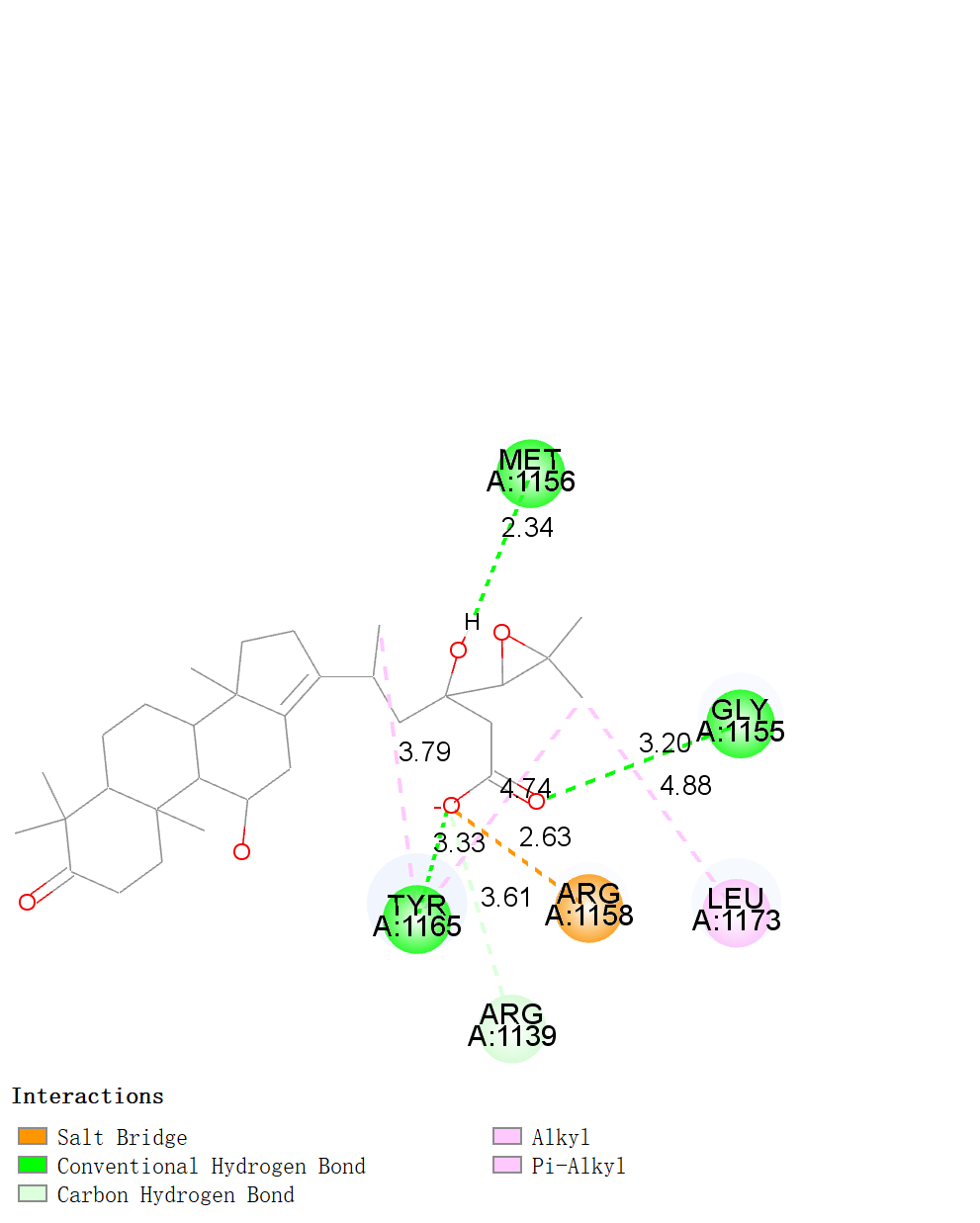

Supplement: Supplementary file 10 [file DataSheet2.zip › Treat/IGFBIG 3.png]

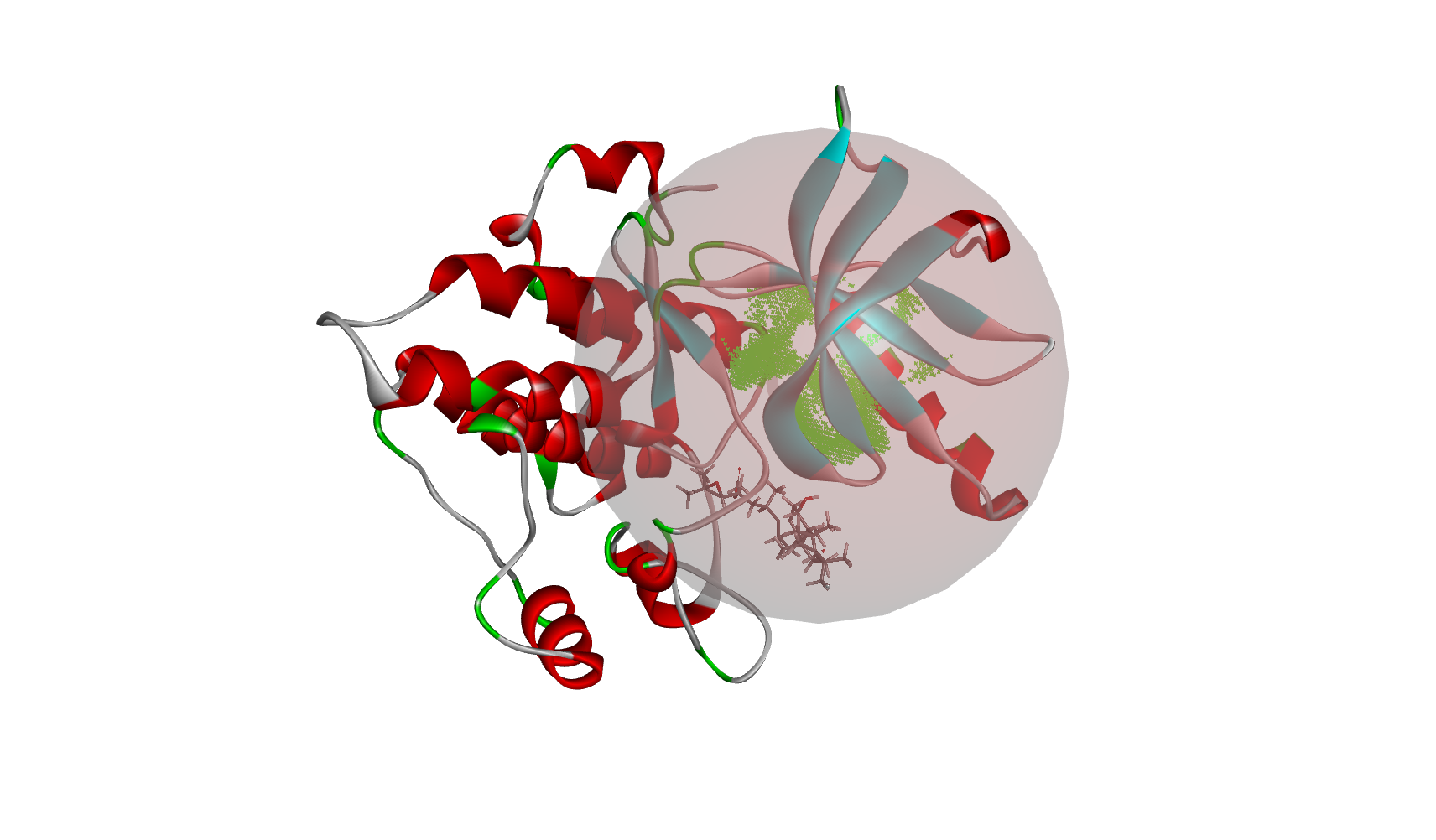

Supplement: Supplementary file 10 [file DataSheet2.zip › Treat/IGFBM1.png]

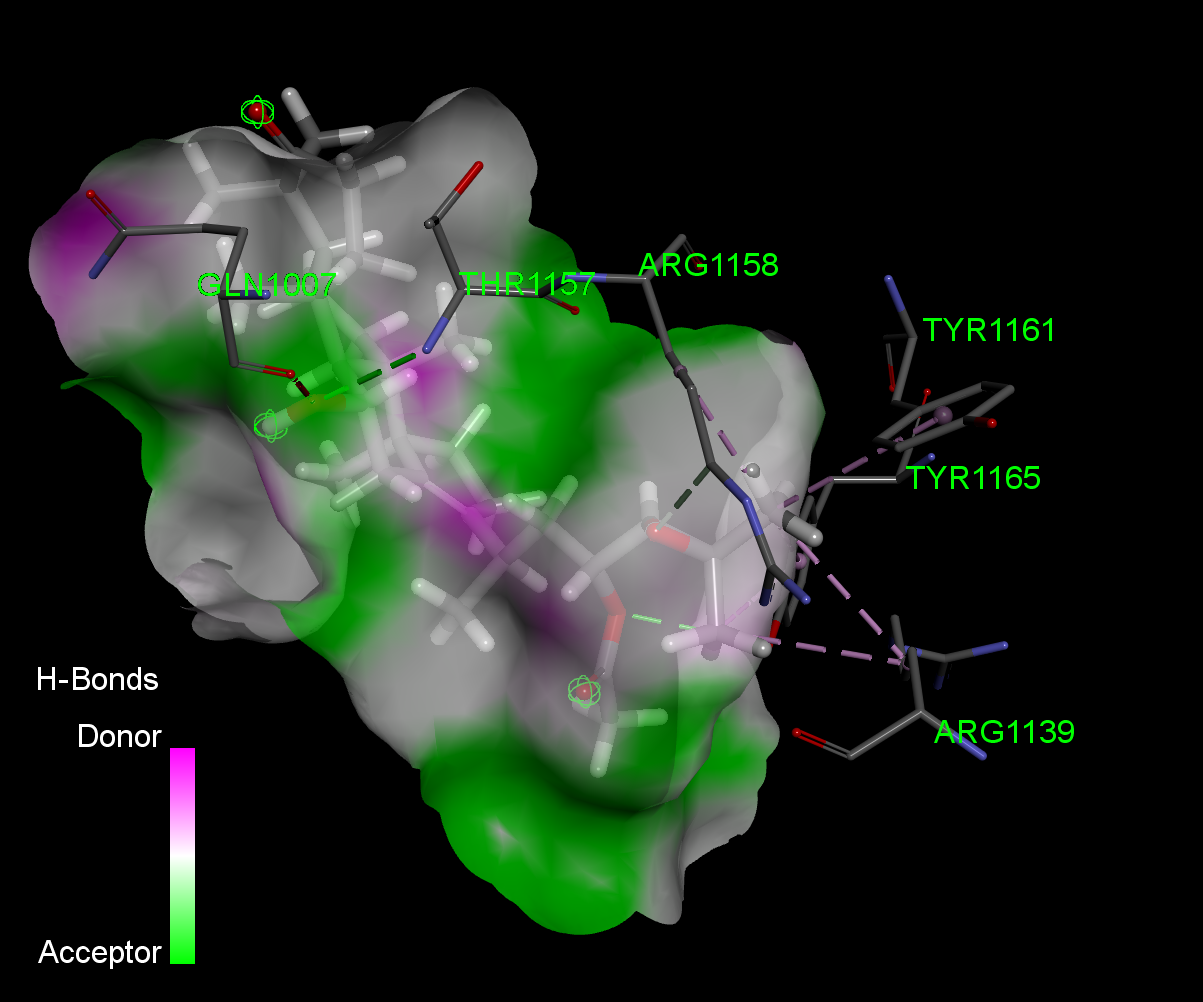

Supplement: Supplementary file 10 [file DataSheet2.zip › Treat/IGFBM2.png]

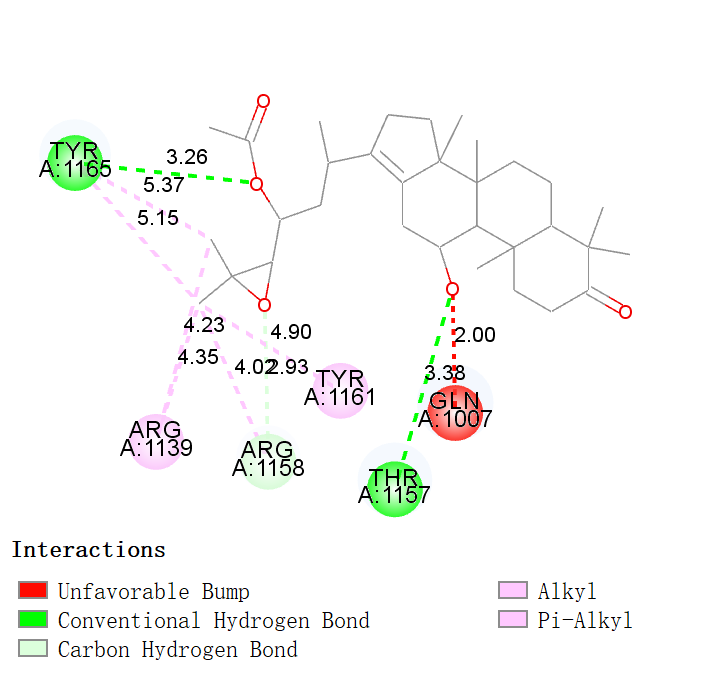

Supplement: Supplementary file 10 [file DataSheet2.zip › Treat/IGFBM3.png]

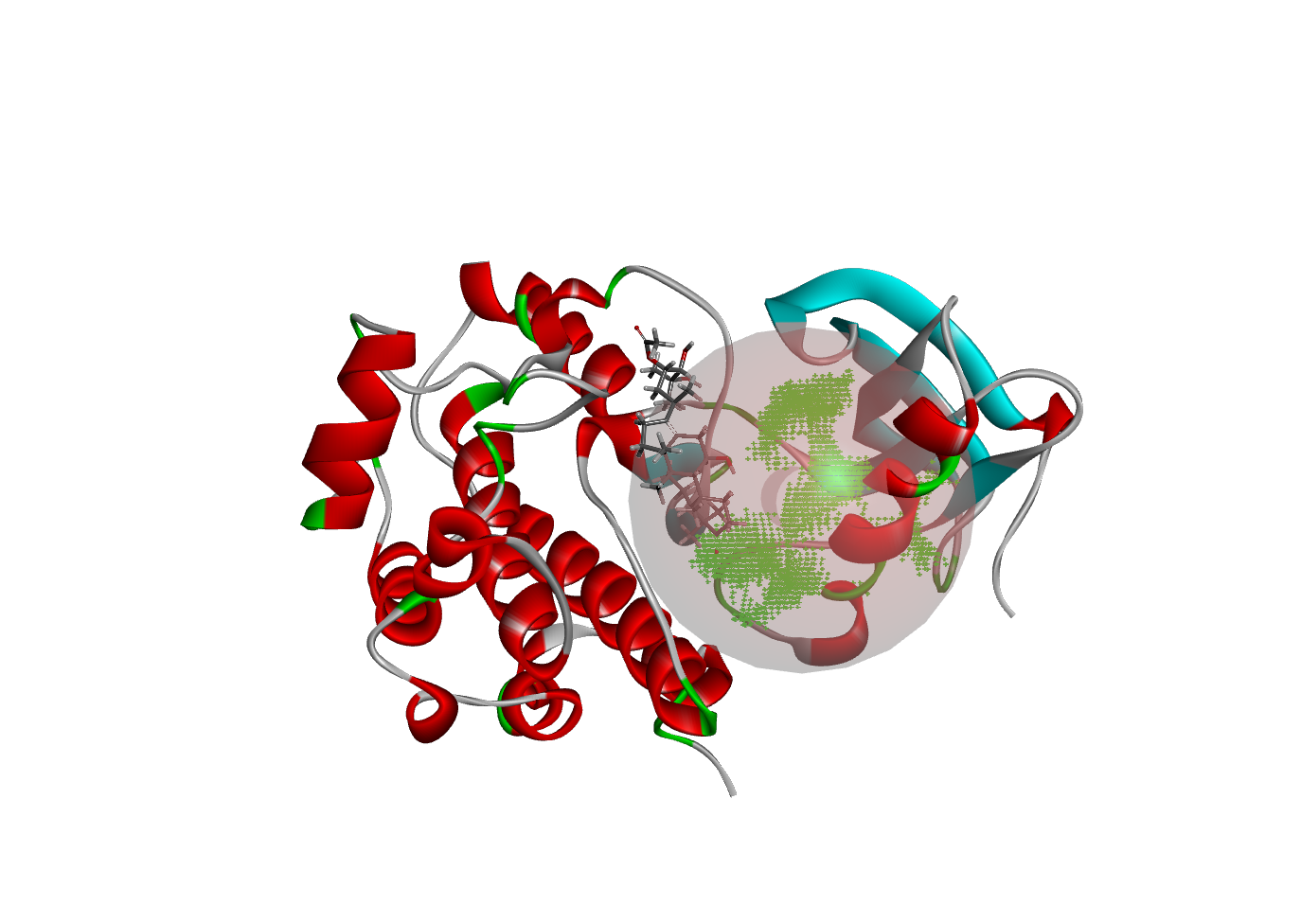

Supplement: Supplementary file 10 [file DataSheet2.zip › Treat/IGFE23 1.png]

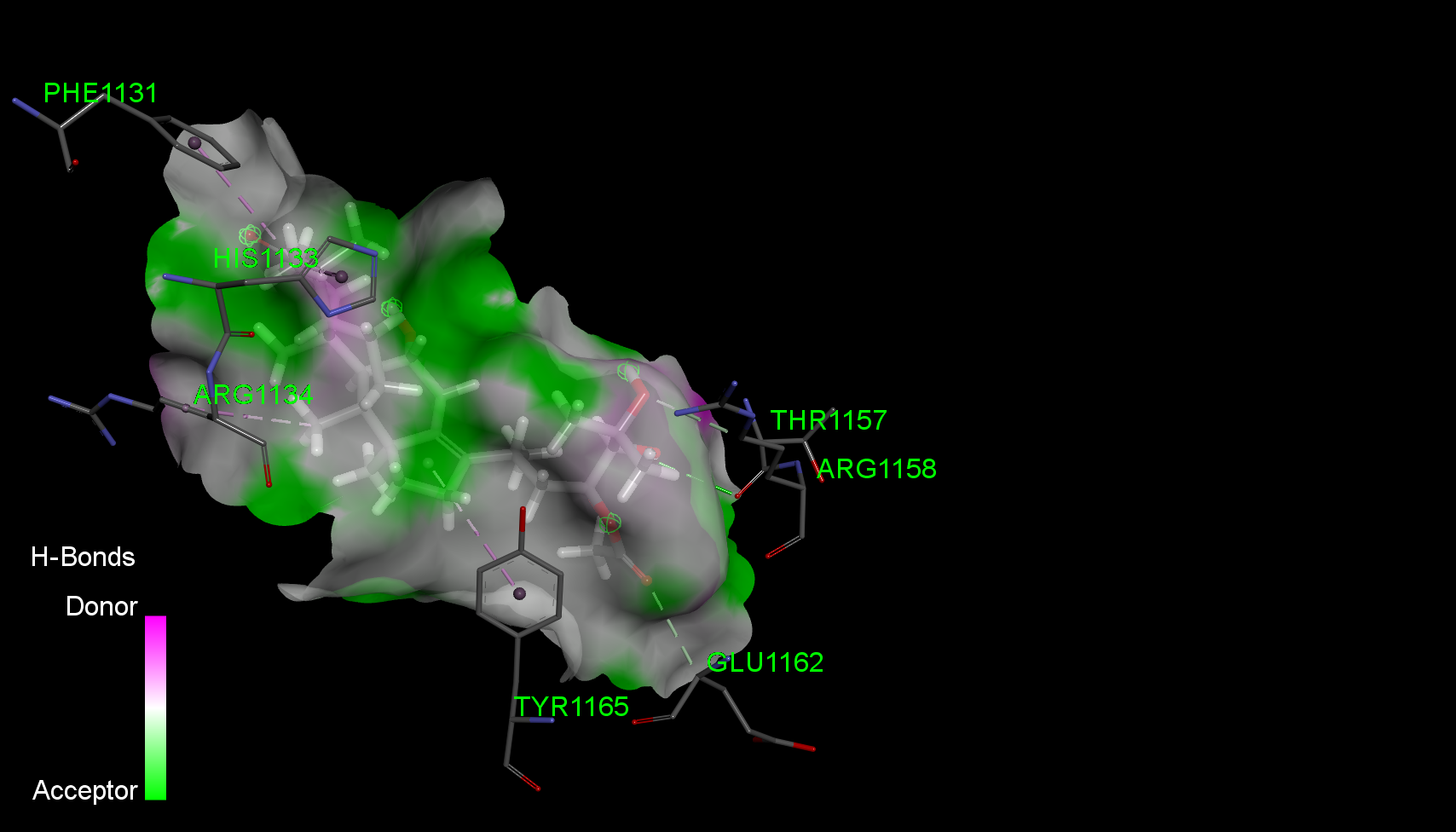

Supplement: Supplementary file 10 [file DataSheet2.zip › Treat/IGFE23 2.png]

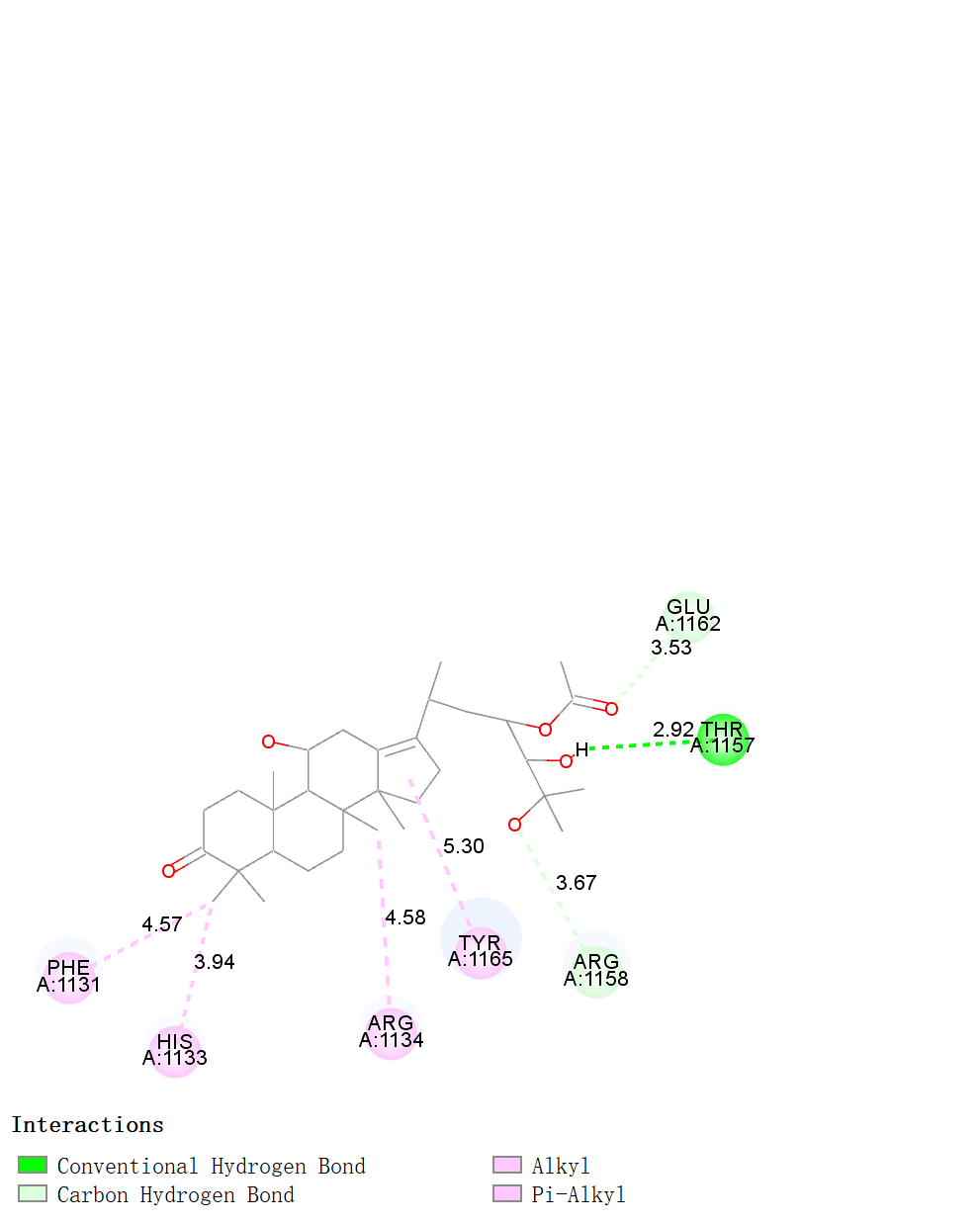

Supplement: Supplementary file 10 [file DataSheet2.zip › Treat/IGFE23 3.png]

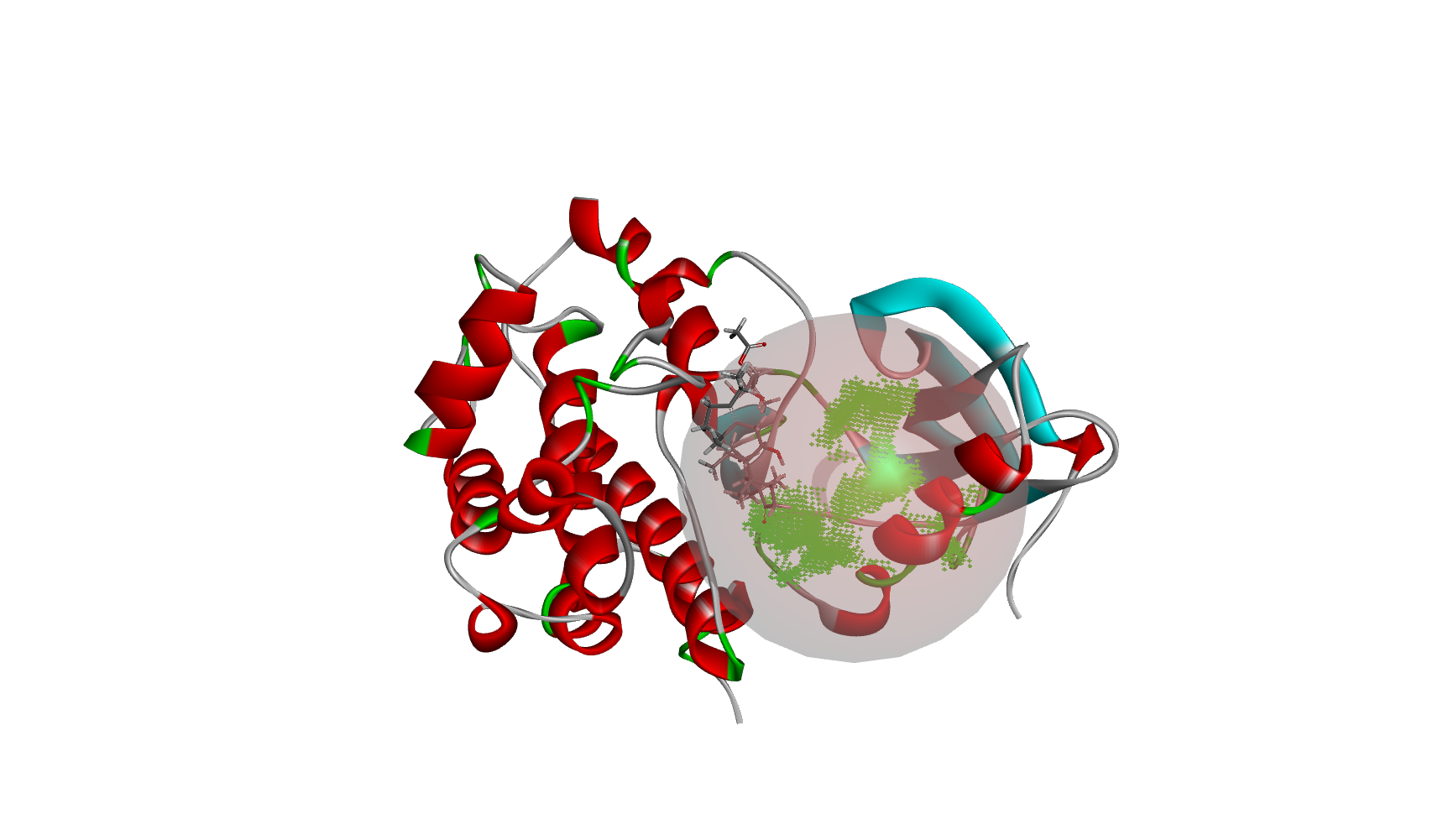

Supplement: Supplementary file 10 [file DataSheet2.zip › Treat/IGFE24 1.png]

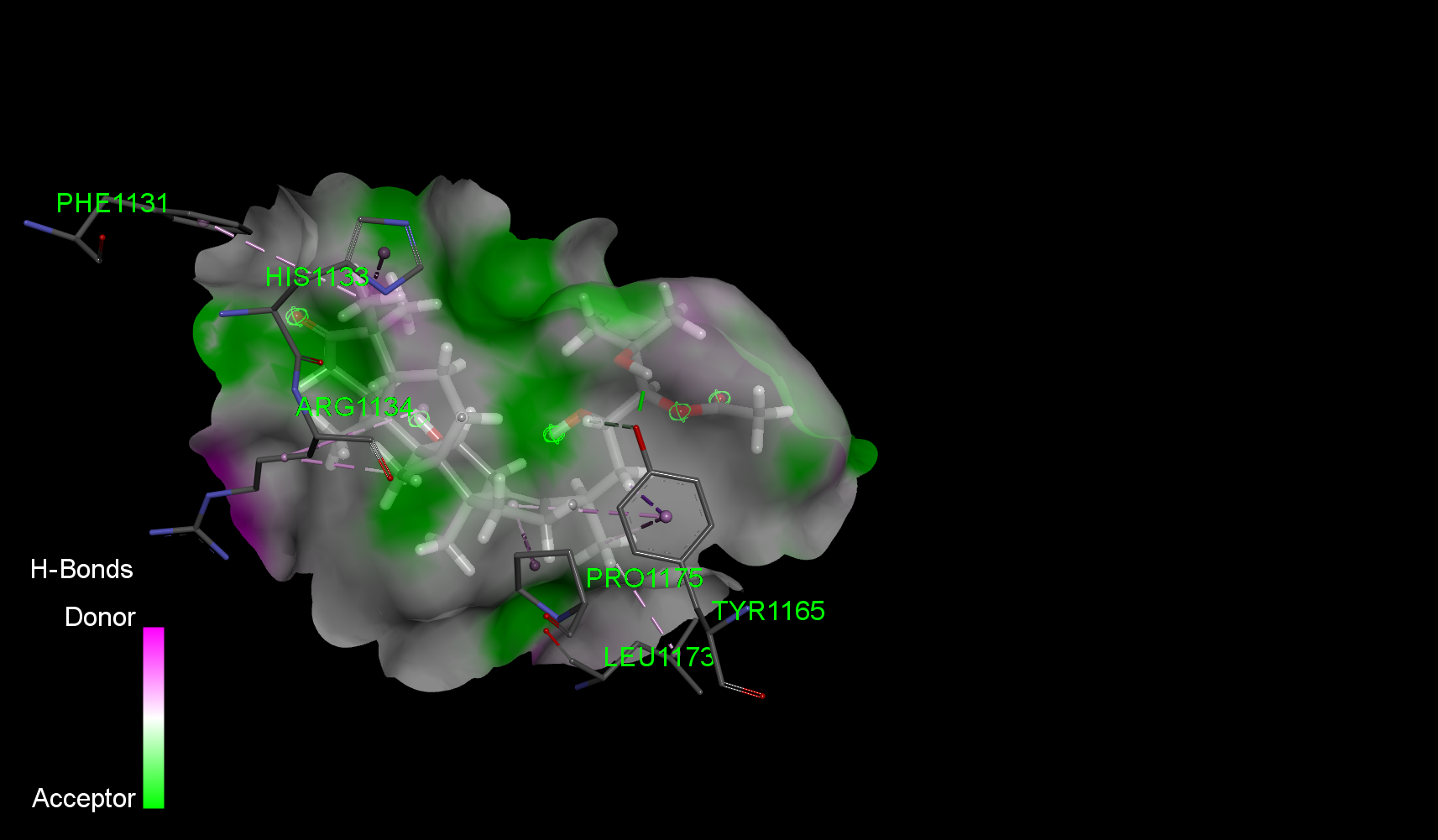

Supplement: Supplementary file 10 [file DataSheet2.zip › Treat/IGFE24 2.png]

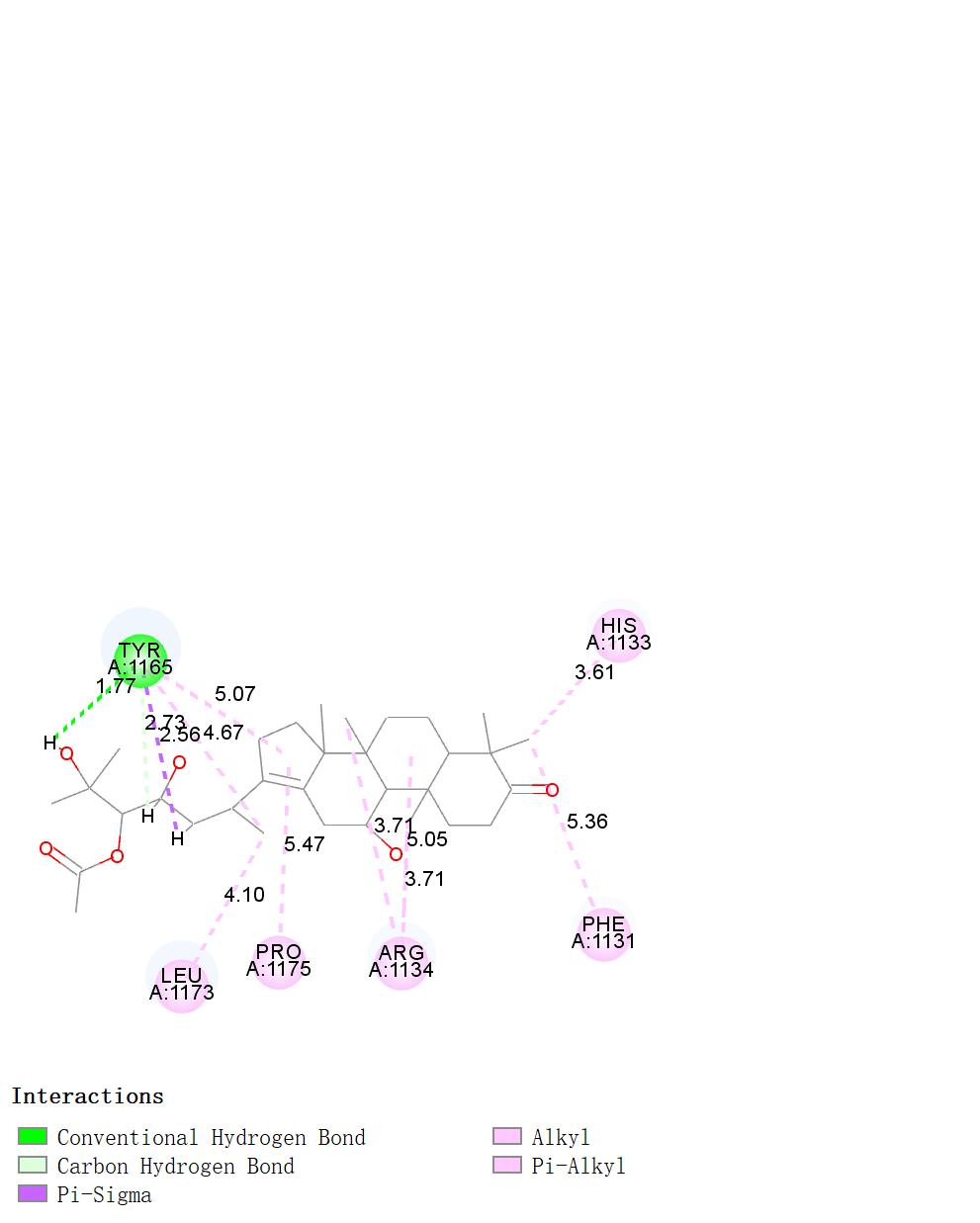

Supplement: Supplementary file 10 [file DataSheet2.zip › Treat/IGFE24 3.png]

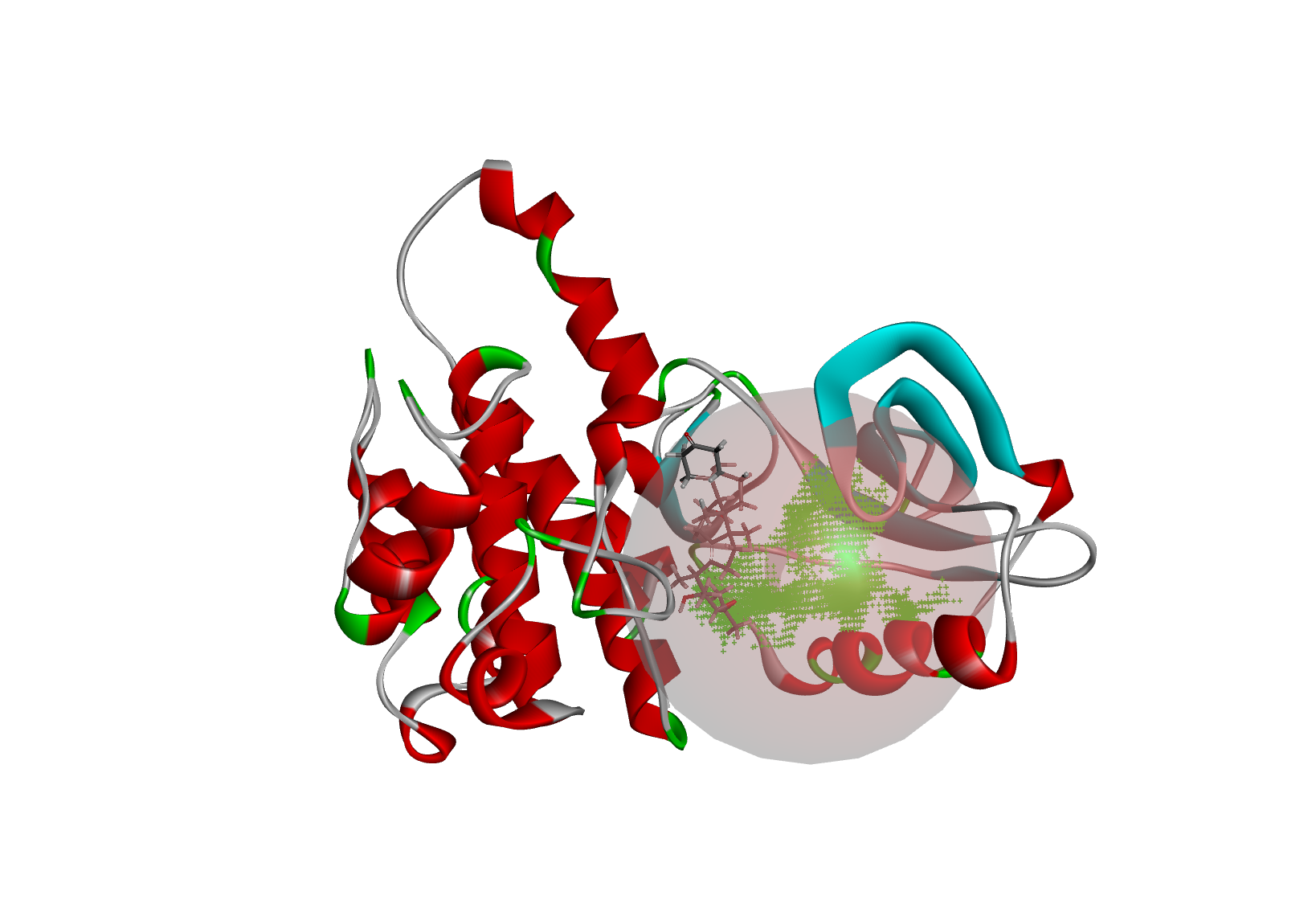

Supplement: Supplementary file 10 [file DataSheet2.zip › Treat/IGFalisolB 1.png]

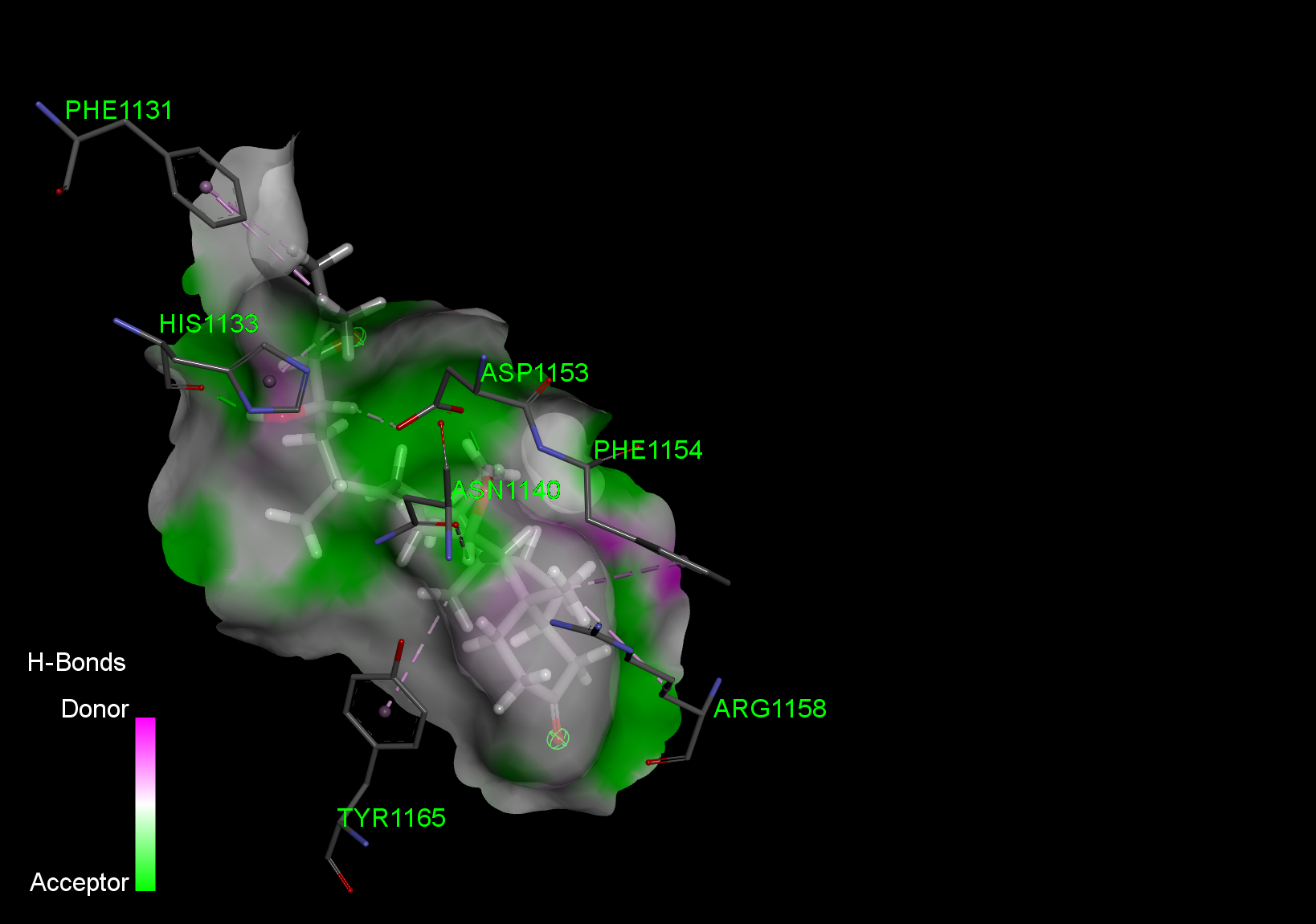

Supplement: Supplementary file 10 [file DataSheet2.zip › Treat/IGFalisolB 2.png]

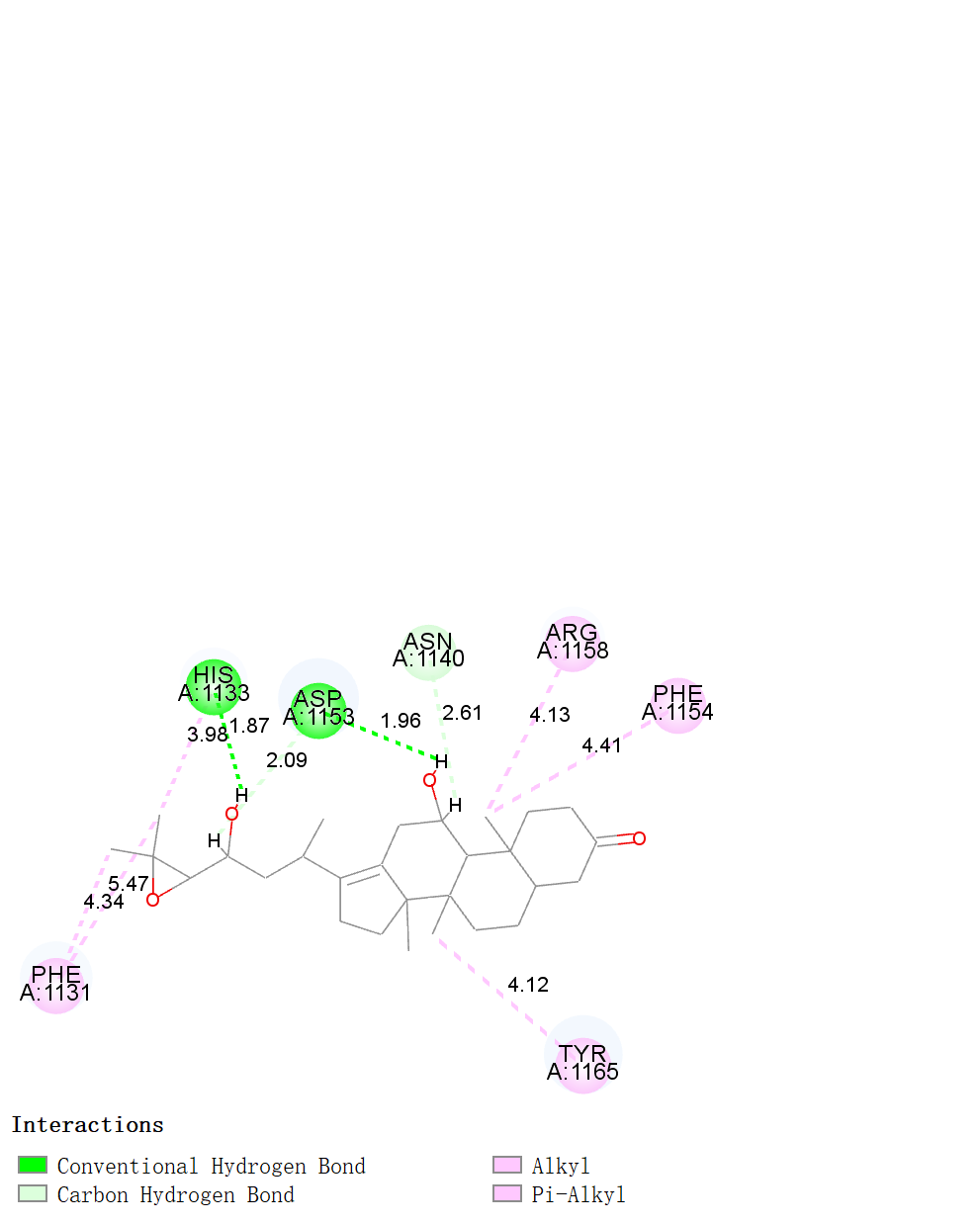

Supplement: Supplementary file 10 [file DataSheet2.zip › Treat/IGFalisolB 3.png]

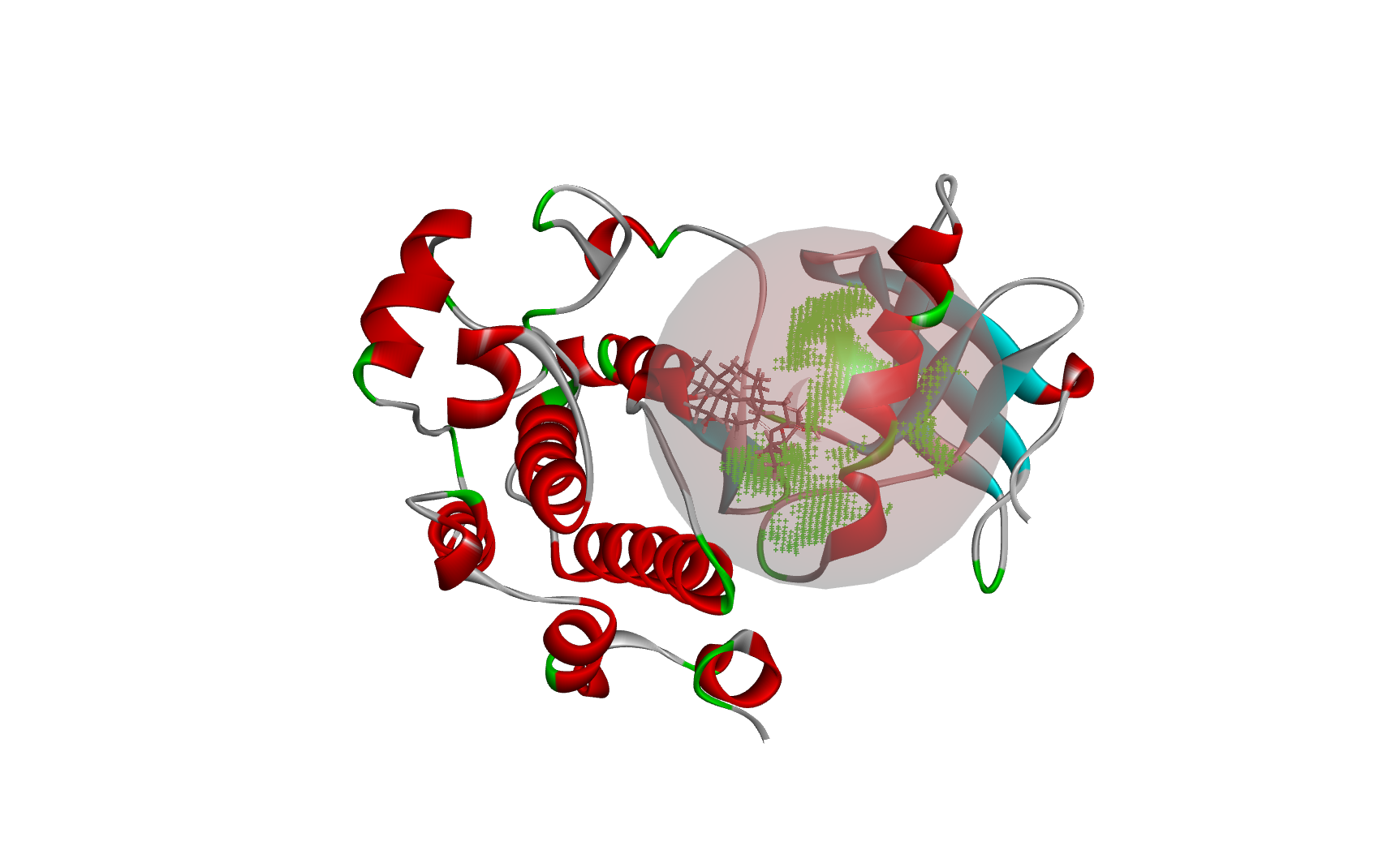

Supplement: Supplementary file 10 [file DataSheet2.zip › Treat/IGFalisolC 1.png]

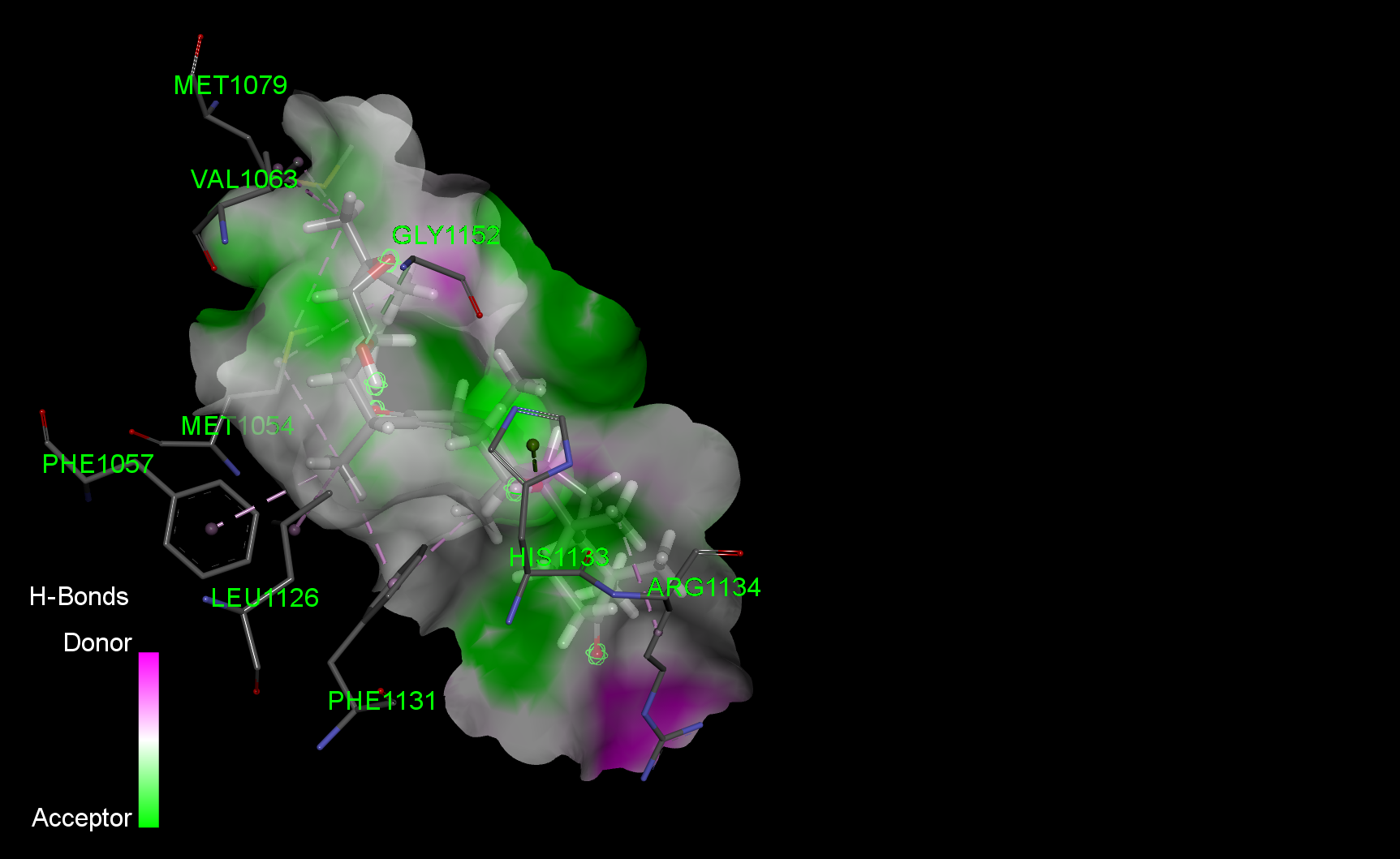

Supplement: Supplementary file 10 [file DataSheet2.zip › Treat/IGFalisolC 2.png]

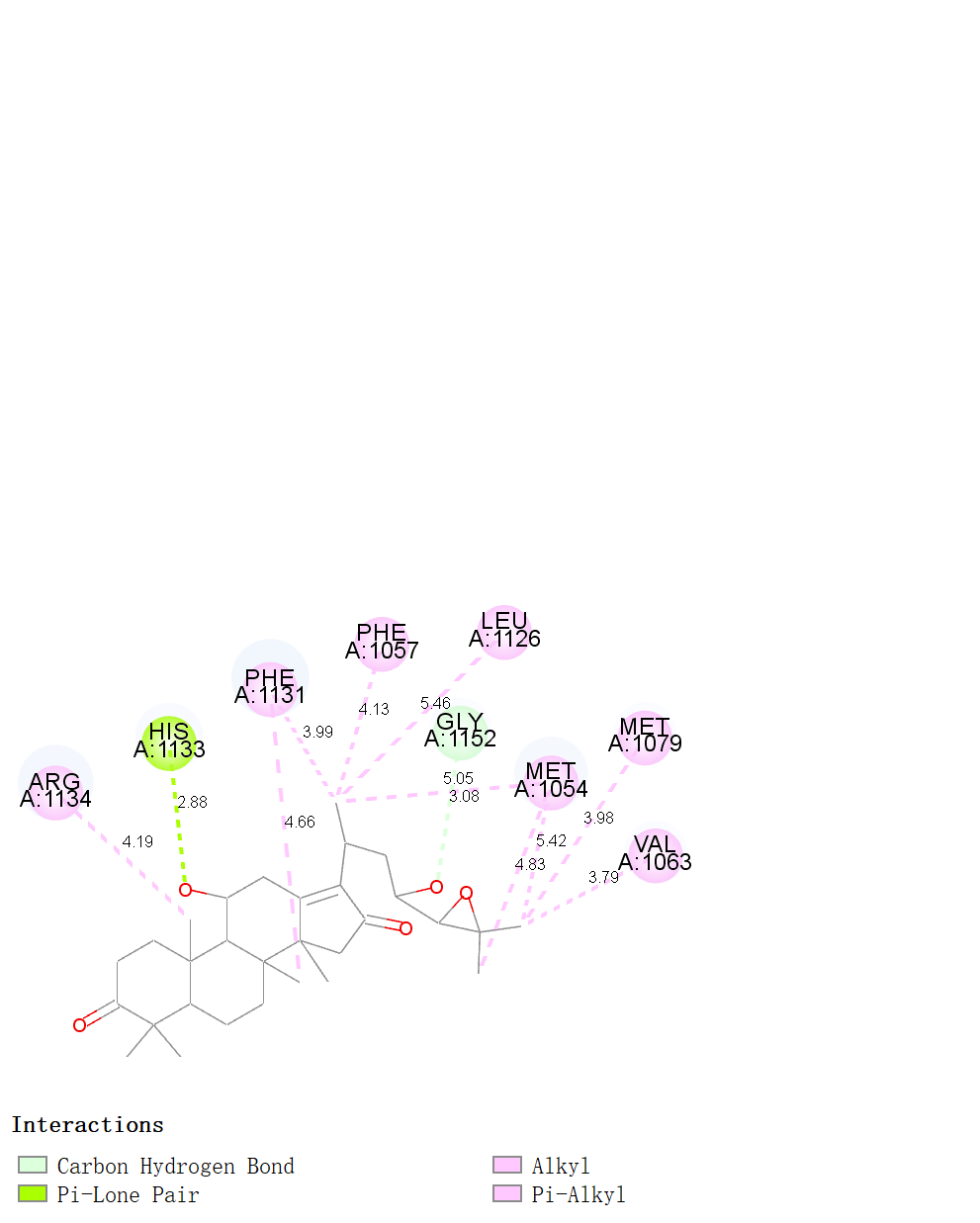

Supplement: Supplementary file 10 [file DataSheet2.zip › Treat/IGFalisolC 3.png]

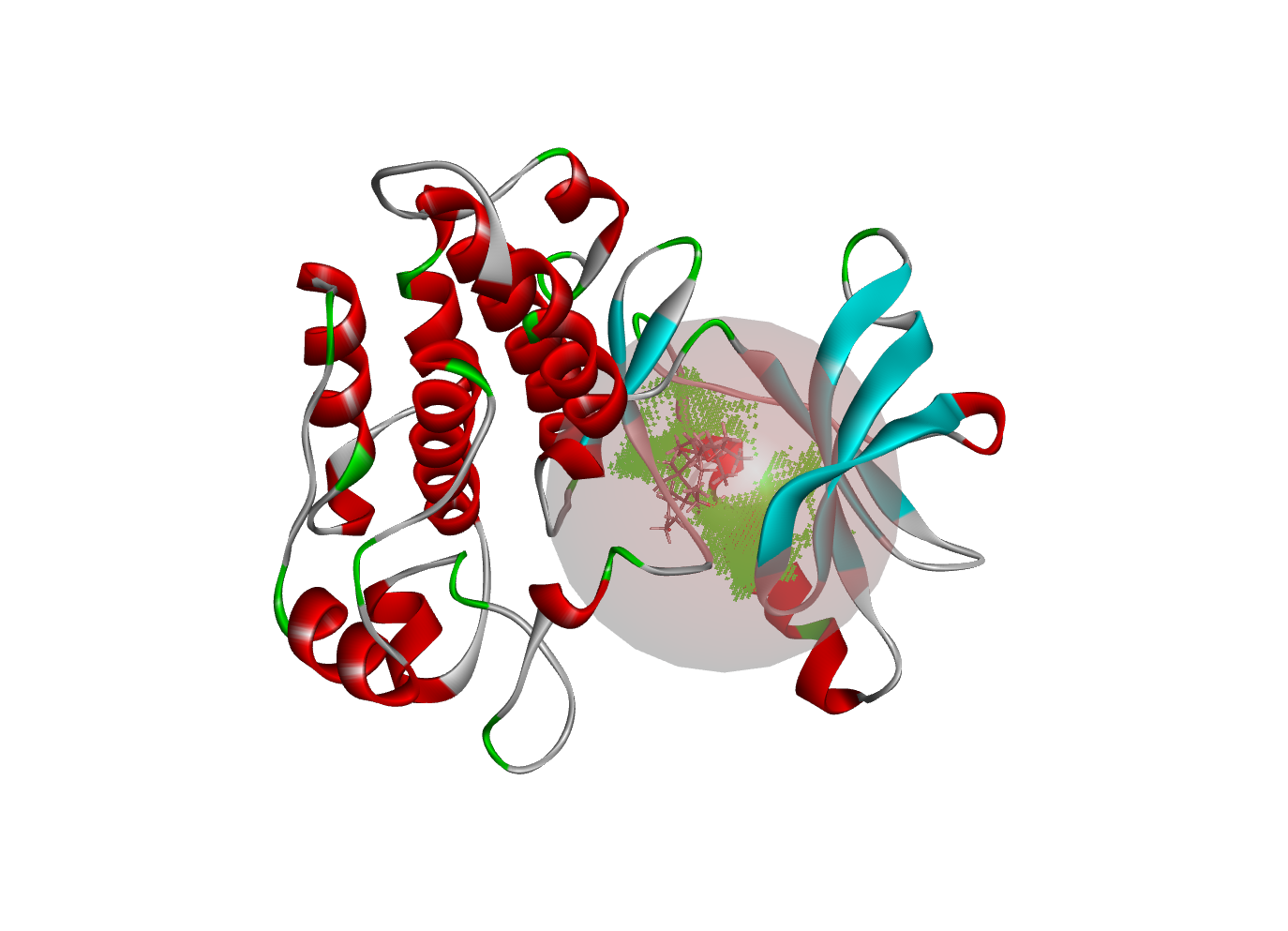

Supplement: Supplementary file 10 [file DataSheet2.zip › Treat/IGFb23 1.png]

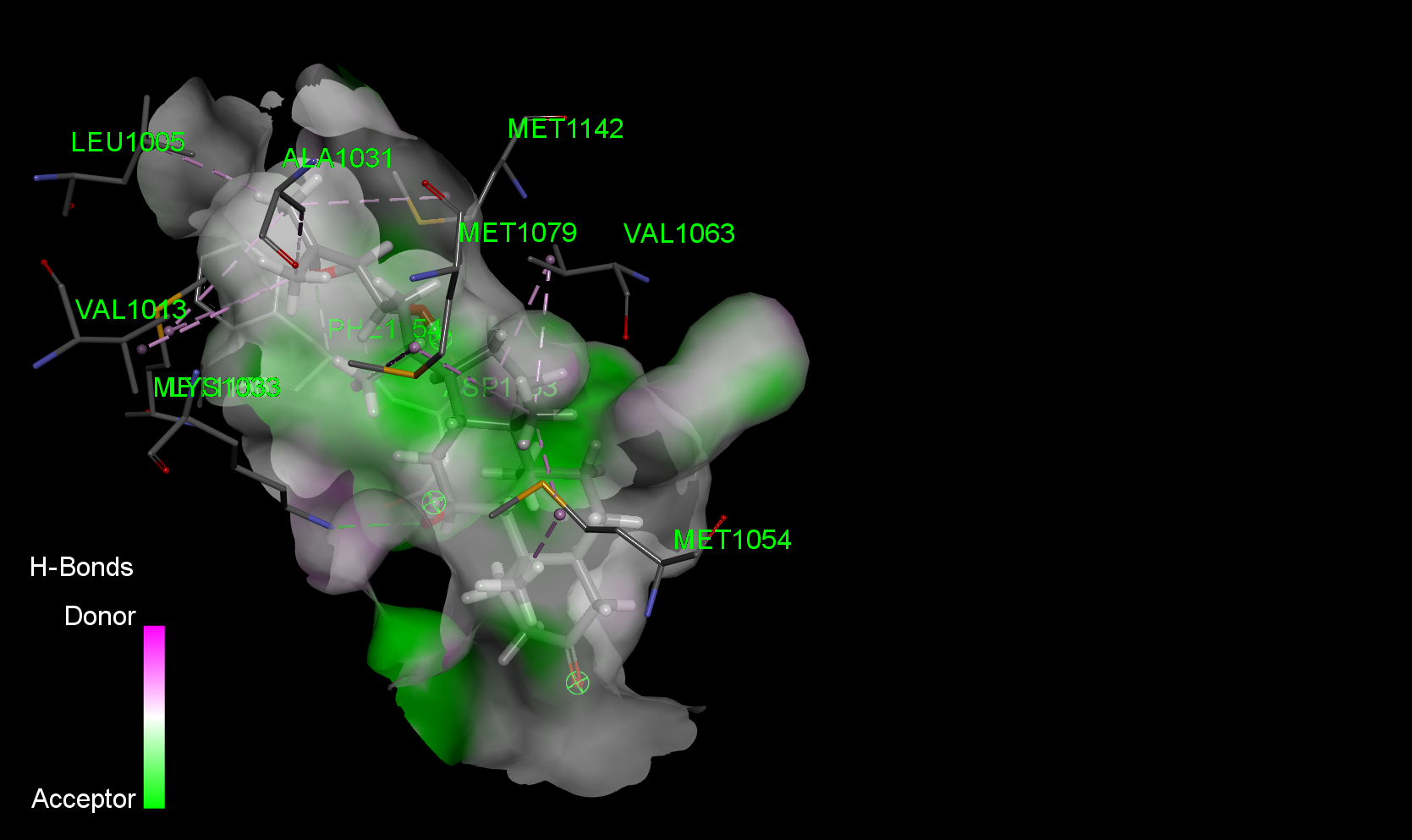

Supplement: Supplementary file 10 [file DataSheet2.zip › Treat/IGFb23 2.png]

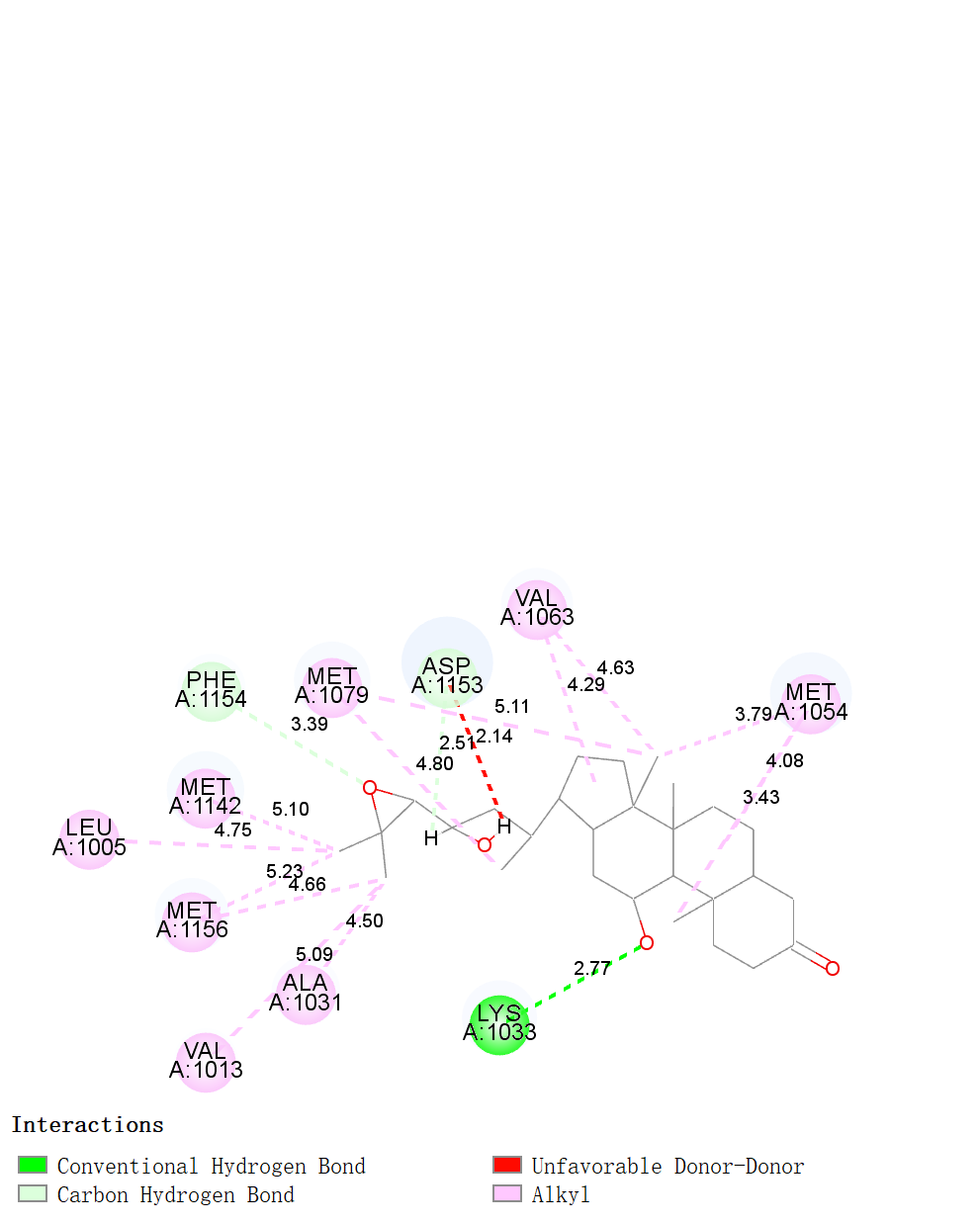

Supplement: Supplementary file 10 [file DataSheet2.zip › Treat/IGFb23 3.png]

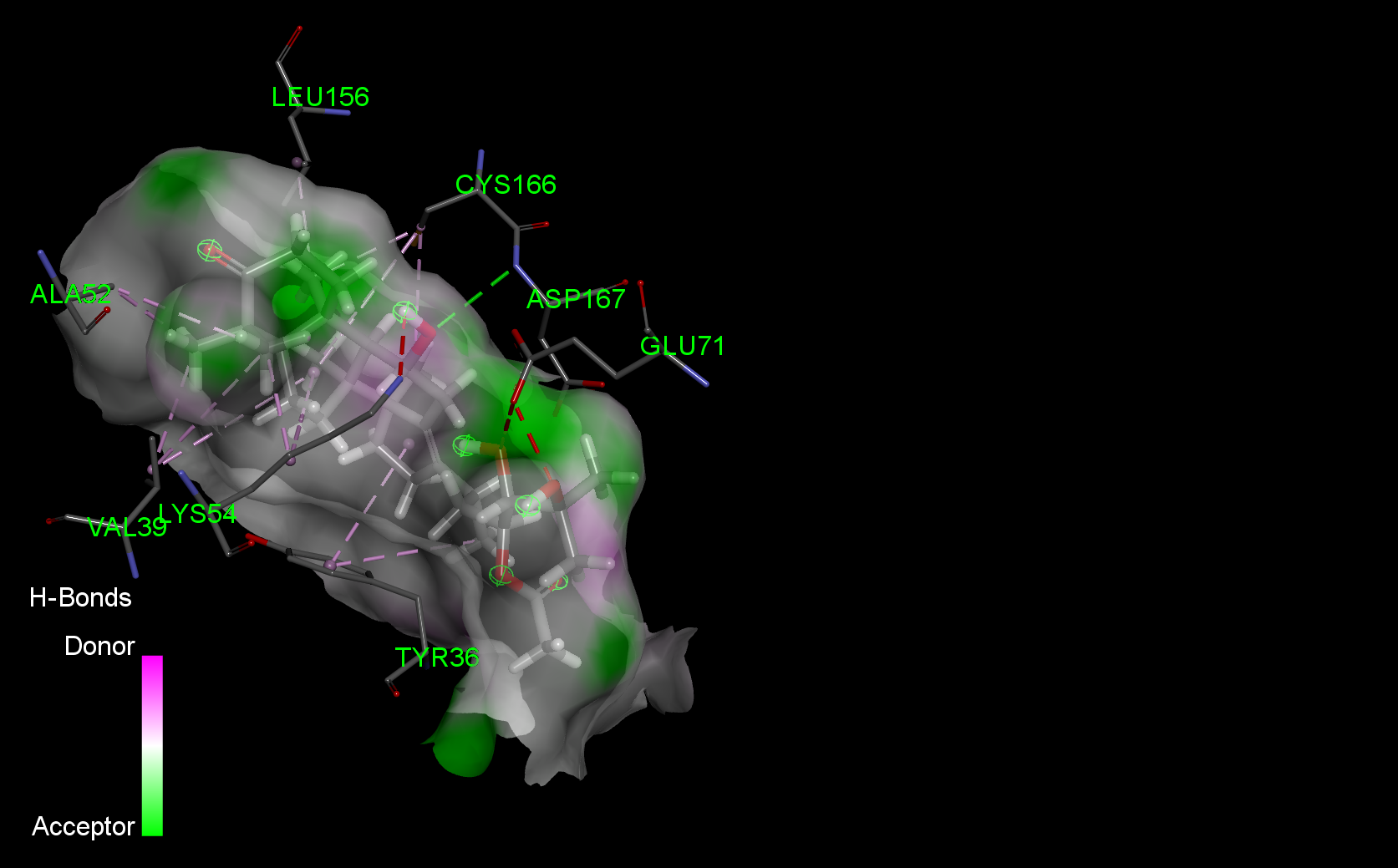

Supplement: Supplementary file 10 [file DataSheet2.zip › Treat/MAPKE23 2.png]

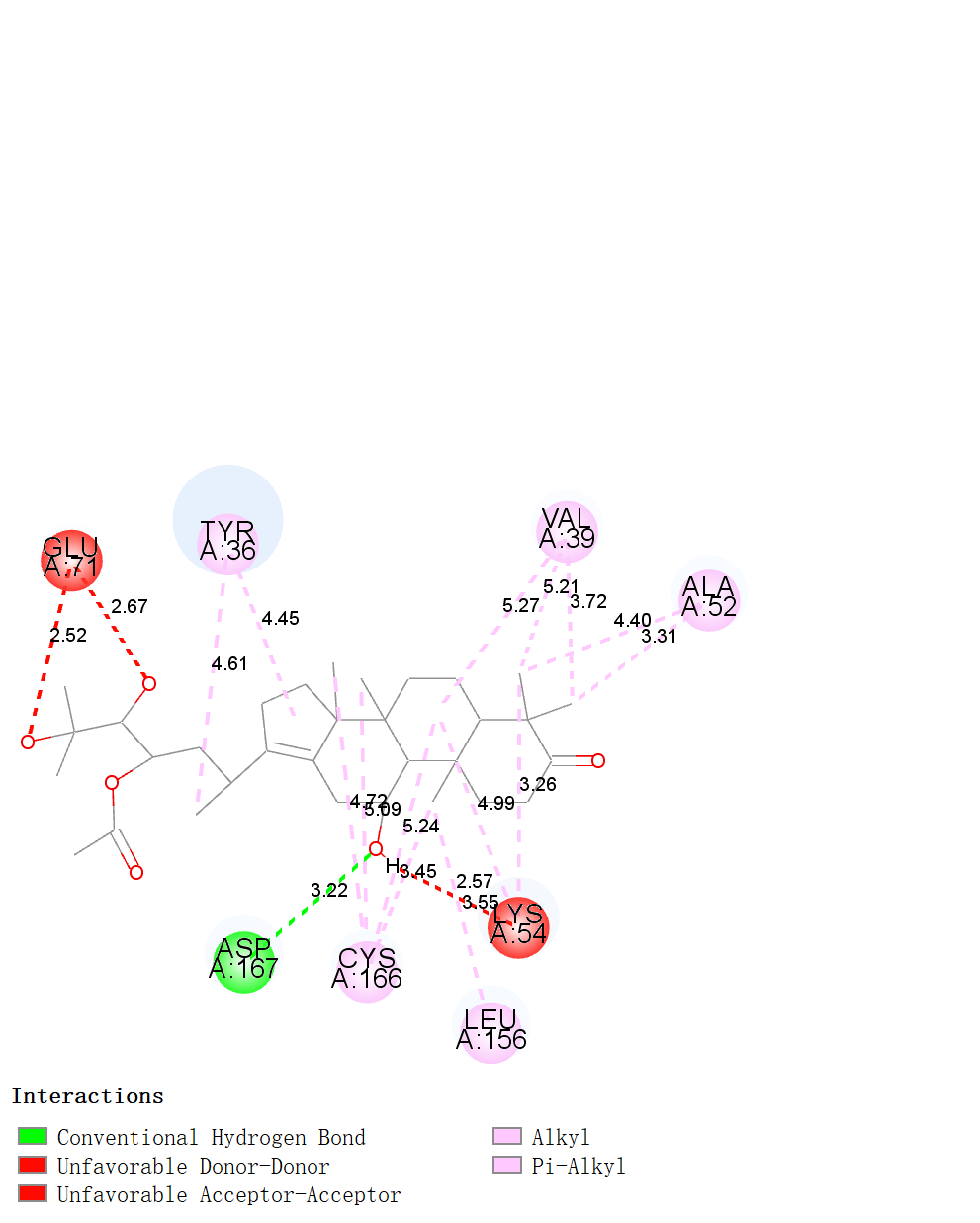

Supplement: Supplementary file 10 [file DataSheet2.zip › Treat/MAPKE23 3.png]

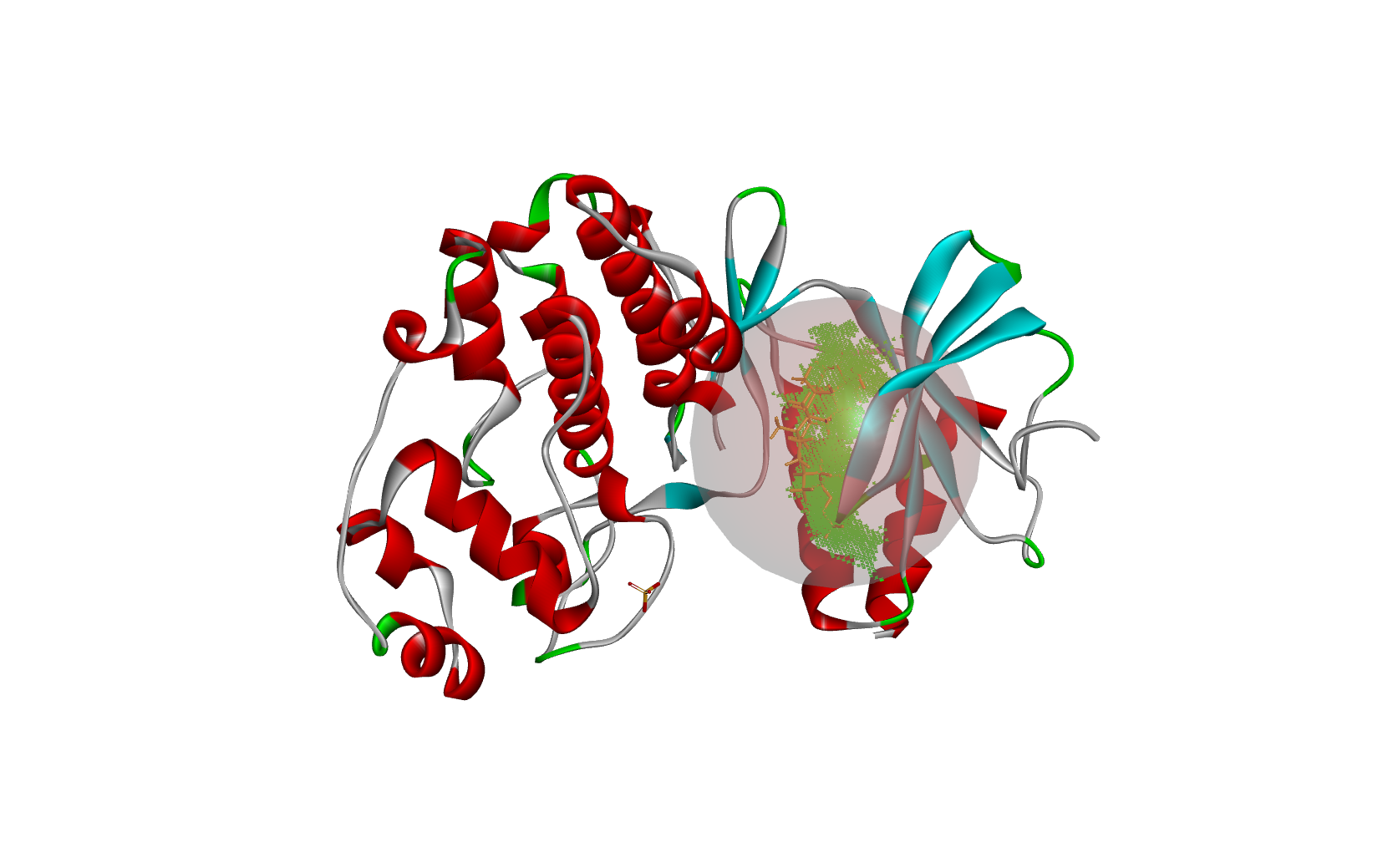

Supplement: Supplementary file 10 [file DataSheet2.zip › Treat/MAPKE23.png]
