# Supplementary material for: Exploring the mechanism of Alisma orientale for the treatment of pregnancy induced hypertension and potential hepato-nephrotoxicity by using network pharmacology, network toxicology, molecular docking and molecular dynamics simulation
Source: Front Pharmacol. 2022 Nov 15;13:1027112. doi: 10.3389/fphar.2022.1027112 (PMC9705790; doi:10.3389/fphar.2022.1027112)
Supplement: Supplementary file 4 [file DataSheet8.ZIP › Supplement Materials/GOKEGG/Toxic/Metascape.zip!/Enrichment_GO/ColorByCluster.pdf]

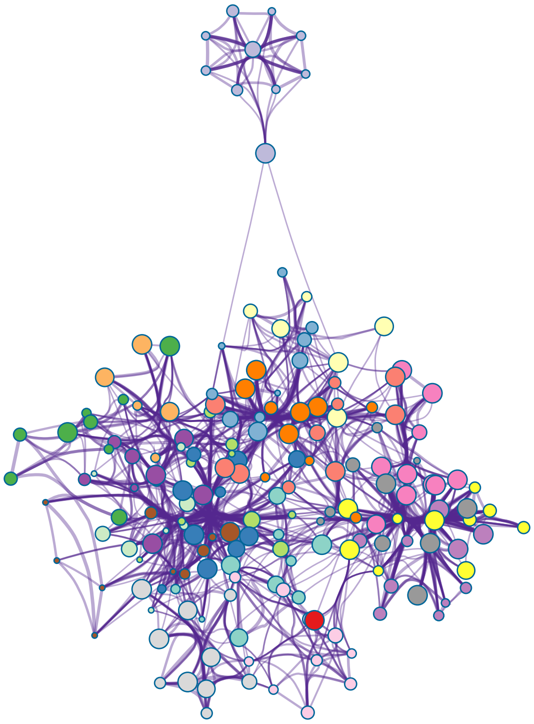

- Pathways in cancer
- response to hormone
- response to nutrient levels
- cellular response to nitrogen compound
- positive regulation of protein localization
- PI3K-Akt signaling pathway
- response to steroid hormone
- positive regulation of protein phosphorylation
- protein phosphorylation
- Chemical carcinogenesis - receptor activation
- regulation of defense response
- regulation of hormone levels
- response to bacterium
- positive regulation of lipid metabolic process
- response to oxygen levels
- regulation of miRNA transcription
- Breast cancer
- response to UV
- positive regulation of cell migration
- multi-organism reproductive process

created by
http://metascape.org
