# Supplementary material for: Exploring the mechanism of Alisma orientale for the treatment of pregnancy induced hypertension and potential hepato-nephrotoxicity by using network pharmacology, network toxicology, molecular docking and molecular dynamics simulation
Source: Front Pharmacol. 2022 Nov 15;13:1027112. doi: 10.3389/fphar.2022.1027112 (PMC9705790; doi:10.3389/fphar.2022.1027112)
Supplement: Supplementary file 4 [file DataSheet8.ZIP › Supplement Materials/GOKEGG/Toxic/Metascape.zip!/Enrichment_heatmap/HeatmapSelectedGO.pdf]

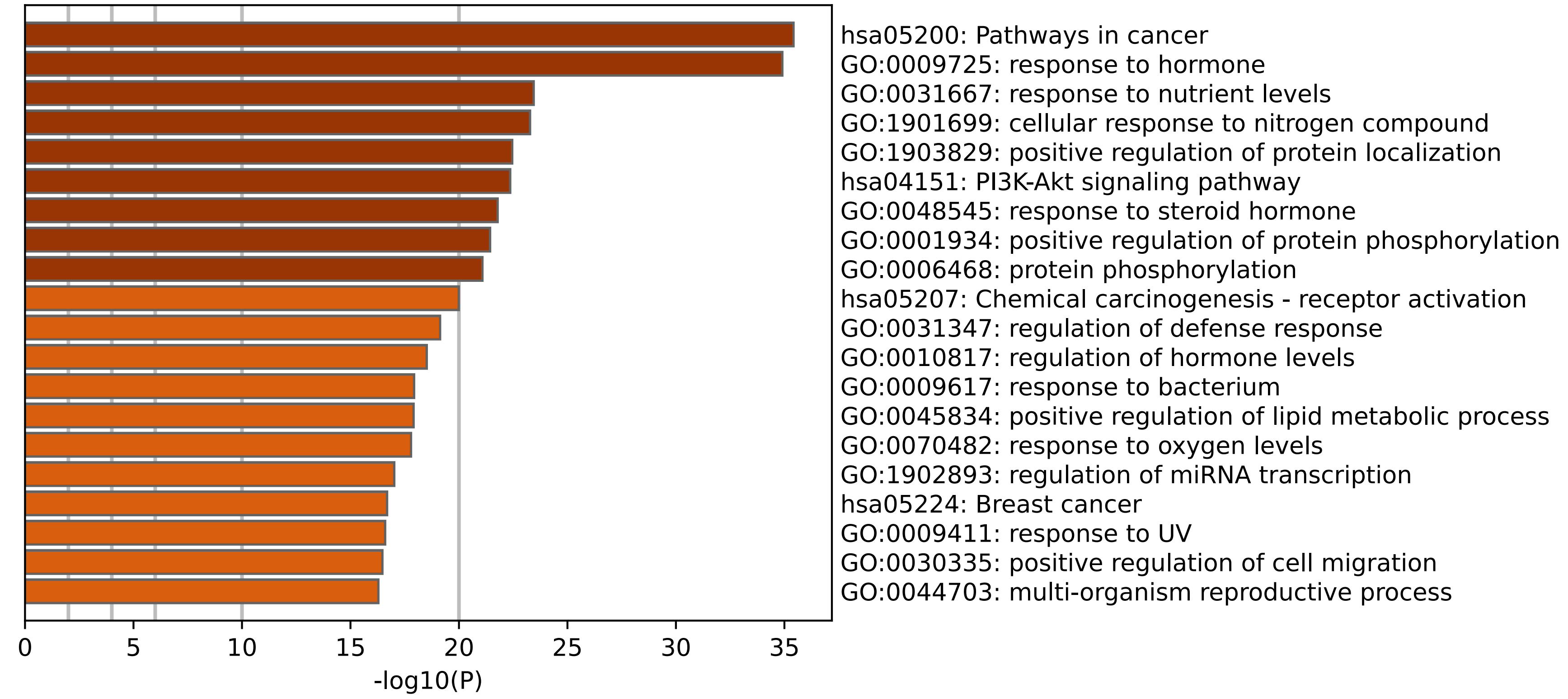
hsa05200: Pathways in cancer
GO:0009725: response to hormone
GO:0031667: response to nutrient levels
GO:1901699: cellular response to nitrogen compound
GO:1903829: positive regulation of protein localization
hsa04151: PI3K-Akt signaling pathway
GO:0048545: response to steroid hormone
GO:0001934: positive regulation of protein phosphorylation
GO:0006468: protein phosphorylation
hsa05207: Chemical carcinogenesis - receptor activation
GO:0031347: regulation of defense response
GO:0010817: regulation of hormone levels
GO:0009617: response to bacterium
GO:0045834: positive regulation of lipid metabolic process
GO:0070482: response to oxygen levels
GO:1902893: regulation of miRNA transcription
hsa05224: Breast cancer
GO:0009411: response to UV
GO:0030335: positive regulation of cell migration
GO:0044703: multi-organism reproductive process
0
5
10
15
20
25
30
35
-log10(P)
