# Supplementary material for: Exploring the mechanism of Alisma orientale for the treatment of pregnancy induced hypertension and potential hepato-nephrotoxicity by using network pharmacology, network toxicology, molecular docking and molecular dynamics simulation
Source: Front Pharmacol. 2022 Nov 15;13:1027112. doi: 10.3389/fphar.2022.1027112 (PMC9705790; doi:10.3389/fphar.2022.1027112)
Supplement: Supplementary file 4 [file DataSheet8.ZIP › Supplement Materials/GOKEGG/Toxic/Metascape.zip!/Enrichment_heatmap/HeatmapSelectedGOTop100.pdf]

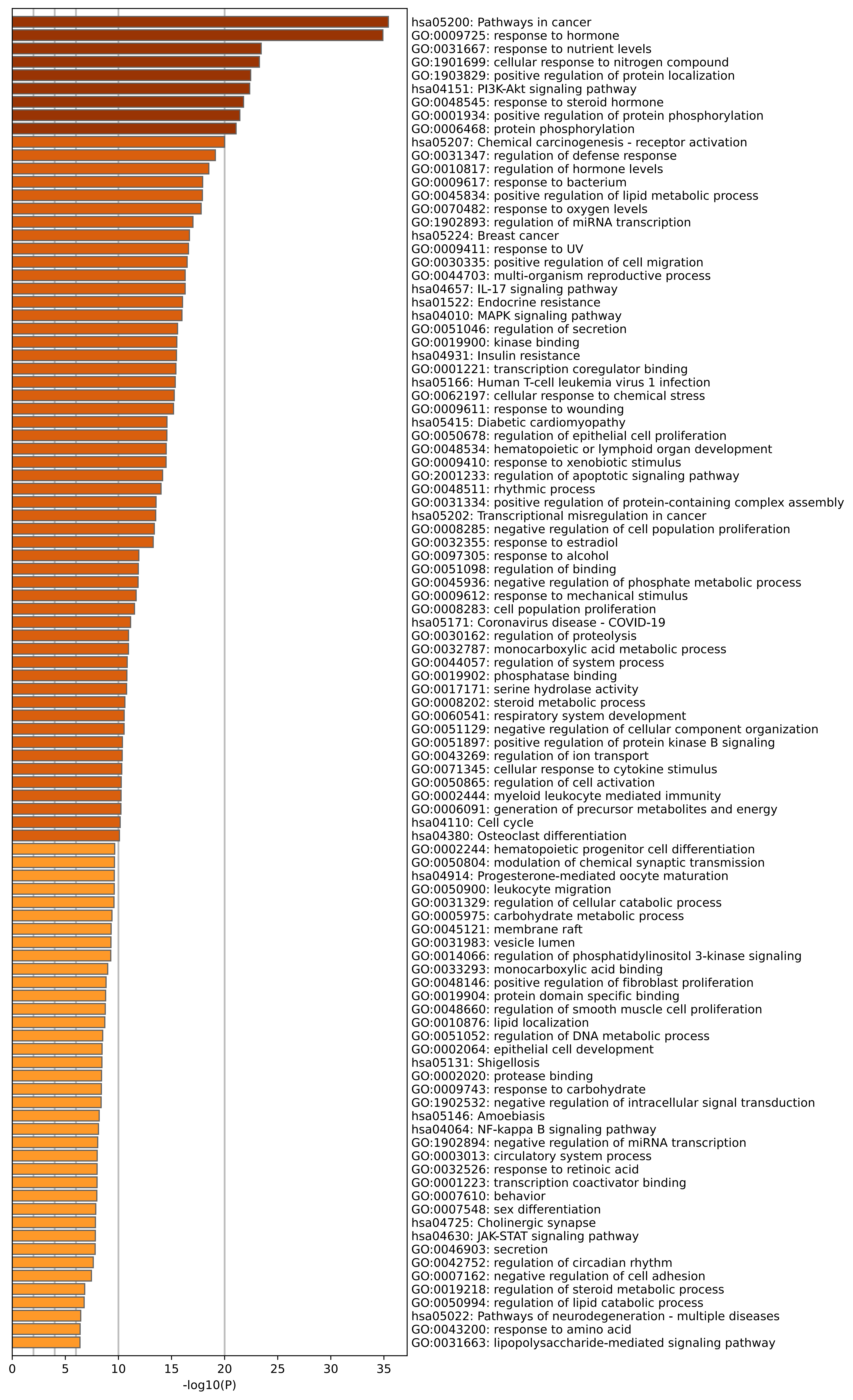
hsa05200: Pathways in cancer
GO:0009725: response to hormone
GO:0031667: response to nutrient levels
GO:1901699: cellular response to nitrogen compound
GO:1903829: positive regulation of protein localization
hsa04151: PI3K-Akt signaling pathway
GO:0048545: response to steroid hormone
GO:0001934: positive regulation of protein phosphorylation
GO:0006468: protein phosphorylation
hsa05207: Chemical carcinogenesis - receptor activation
GO:0031347: regulation of defense response
GO:0010817: regulation of hormone levels
GO:0009617: response to bacterium
GO:0045834: positive regulation of lipid metabolic process
GO:0070482: response to oxygen levels
GO:1902893: regulation of miRNA transcription
hsa05224: Breast cancer
GO:0009411: response to UV
GO:0030335: positive regulation of cell migration
GO:0044703: multi-organism reproductive process
hsa04657: IL-17 signaling pathway
hsa01522: Endocrine resistance
hsa04010: MAPK signaling pathway
GO:0051046: regulation of secretion
GO:0019900: kinase binding
hsa04931: Insulin resistance
GO:0001221: transcription coregulator binding
hsa05166: Human T-cell leukemia virus 1 infection
GO:0062197: cellular response to chemical stress
GO:0009611: response to wounding
hsa05415: Diabetic cardiomyopathy
GO:0050678: regulation of epithelial cell proliferation
GO:0048534: hematopoietic or lymphoid organ development
GO:0009410: response to xenobiotic stimulus
GO:2001233: regulation of apoptotic signaling pathway
GO:0048511: rhythmic process
GO:0031334: positive regulation of protein-containing complex assembly
hsa05202: Transcriptional misregulation in cancer
GO:0008285: negative regulation of cell population proliferation
GO:0032355: response to estradiol
GO:0097305: response to alcohol
GO:0051098: regulation of binding
GO:0045936: negative regulation of phosphate metabolic process
GO:0009612: response to mechanical stimulus
GO:0008283: cell population proliferation
hsa05171: Coronavirus disease - COVID-19
GO:0030162: regulation of proteolysis
GO:0032787: monocarboxylic acid metabolic process
GO:0044057: regulation of system process
GO:0019902: phosphatase binding
GO:0017171: serine hydrolase activity
GO:0008202: steroid metabolic process
GO:0060541: respiratory system development
GO:0051129: negative regulation of cellular component organization
GO:0051897: positive regulation of protein kinase B signaling
GO:0043269: regulation of ion transport
GO:0071345: cellular response to cytokine stimulus
GO:0050865: regulation of cell activation
GO:0002444: myeloid leukocyte mediated immunity
GO:0006091: generation of precursor metabolites and energy
hsa04110: Cell cycle
hsa04380: Osteoclast differentiation
GO:0002244: hematopoietic progenitor cell differentiation
GO:0050804: modulation of chemical synaptic transmission
hsa04914: Progesterone-mediated oocyte maturation
GO:0050900: leukocyte migration
GO:0031329: regulation of cellular catabolic process
GO:0005975: carbohydrate metabolic process
GO:0045121: membrane raft
GO:0031983: vesicle lumen
GO:0014066: regulation of phosphatidylinositol 3-kinase signaling
GO:0033293: monocarboxylic acid binding
GO:0048146: positive regulation of fibroblast proliferation
GO:0019904: protein domain specific binding
GO:0048660: regulation of smooth muscle cell proliferation
GO:0010876: lipid localization
GO:0051052: regulation of DNA metabolic process
GO:0002064: epithelial cell development
hsa05131: Shigellosis
GO:0002020: protease binding
GO:0009743: response to carbohydrate
GO:1902532: negative regulation of intracellular signal transduction
hsa05146: Amoebiasis
hsa04064: NF-kappa B signaling pathway
GO:1902894: negative regulation of miRNA transcription
GO:0003013: circulatory system process
GO:0032526: response to retinoic acid
GO:0001223: transcription coactivator binding
GO:0007610: behavior
GO:0007548: sex differentiation
hsa04725: Cholinergic synapse
hsa04630: JAK-STAT signaling pathway
GO:0046903: secretion
GO:0042752: regulation of circadian rhythm
GO:0007162: negative regulation of cell adhesion
GO:0019218: regulation of steroid metabolic process
GO:0050994: regulation of lipid catabolic process
hsa05022: Pathways of neurodegeneration - multiple diseases
GO:0043200: response to amino acid
GO:0031663: lipopolysaccharide-mediated signaling pathway
0
5
10
15
20
25
30
35
-log10(P)
