# Supplementary material for: Exploring the mechanism of Alisma orientale for the treatment of pregnancy induced hypertension and potential hepato-nephrotoxicity by using network pharmacology, network toxicology, molecular docking and molecular dynamics simulation
Source: Front Pharmacol. 2022 Nov 15;13:1027112. doi: 10.3389/fphar.2022.1027112 (PMC9705790; doi:10.3389/fphar.2022.1027112)
Supplement: Supplementary file 4 [file DataSheet8.ZIP › Supplement Materials/GOKEGG/Toxic/Metascape.zip!/Enrichment_heatmap/HeatmapSelectedGOParent.pdf]

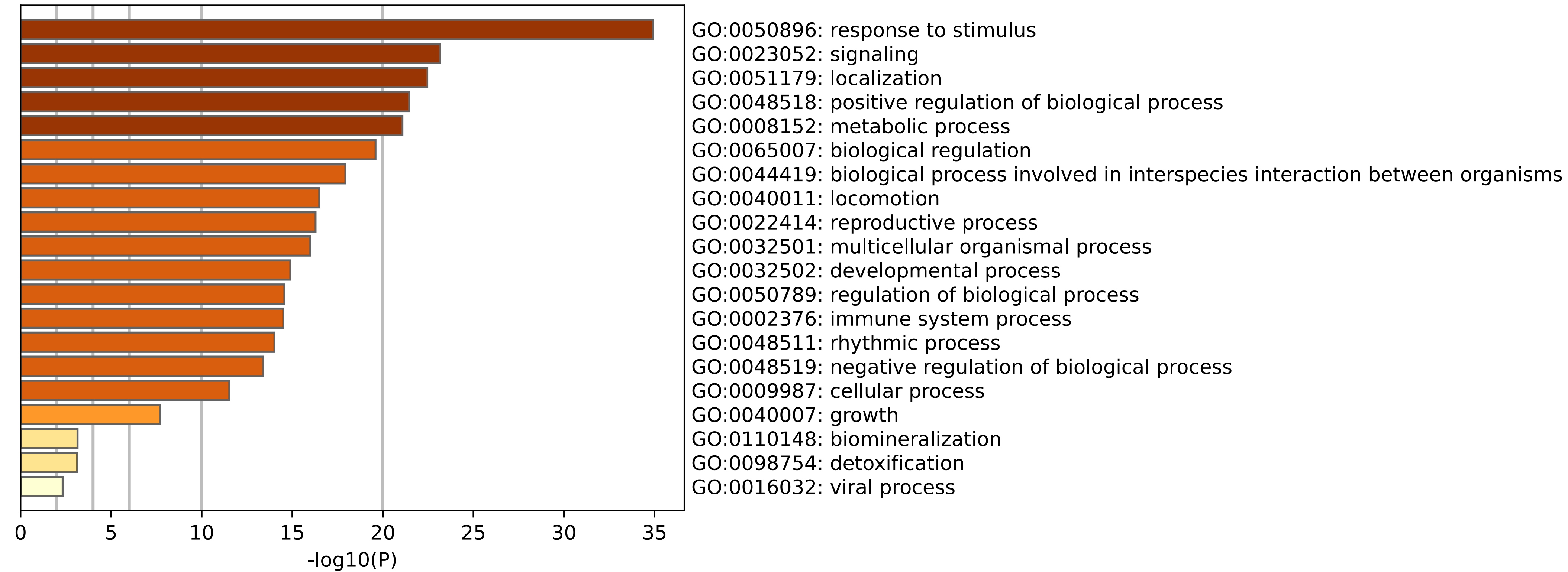
GO:0050896: response to stimulus
GO:0023052: signaling
GO:0051179: localization
GO:0048518: positive regulation of biological process
GO:0008152: metabolic process
GO:0065007: biological regulation
GO:0044419: biological process involved in interspecies interaction between organisms
GO:0040011: locomotion
GO:0022414: reproductive process
GO:0032501: multicellular organismal process
GO:0032502: developmental process
GO:0050789: regulation of biological process
GO:0002376: immune system process
GO:0048511: rhythmic process
GO:0048519: negative regulation of biological process
GO:0009987: cellular process
GO:0040007: growth
GO:0110148: biomineralization
GO:0098754: detoxification
GO:0016032: viral process
0
5
10
15
20
25
30
35
-log10(P)
