# Supplementary material for: Exploring the mechanism of Alisma orientale for the treatment of pregnancy induced hypertension and potential hepato-nephrotoxicity by using network pharmacology, network toxicology, molecular docking and molecular dynamics simulation
Source: Front Pharmacol. 2022 Nov 15;13:1027112. doi: 10.3389/fphar.2022.1027112 (PMC9705790; doi:10.3389/fphar.2022.1027112)
Supplement: Supplementary file 4 [file DataSheet8.ZIP › Supplement Materials/GOKEGG/Toxic/Metascape.zip!/Enrichment_PPI/MyList_MCODE_ALL_PPIColorByCluster.pdf]

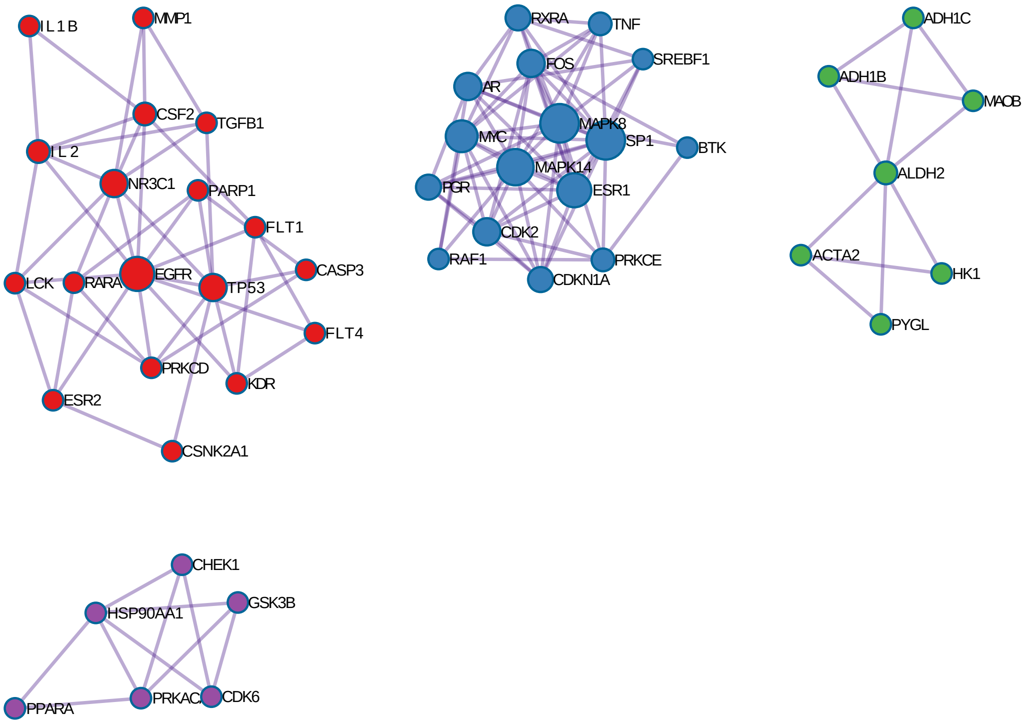

IL1B
MMP1
CSF2
TGFB1
IL2
NR3C1
PARP1
FLT1
CASP3
LCK
RARA
EGFR
TP53
FLT4
PRKCD
KDR
ESR2
CSNK2A1
RXRA
TNF
SREBF1
FOS
AR
MAPK8
SP1
BTK
MYC
MAPK14
PGR
ESR1
CDK2
RAF1
PRKCE
CDKN1A
ADH1C
ADH1B
MAOB
ALDH2
ACTA2
HK1
PYGL
CHEK1
GSK3B
HSP90AA1
PRKAC
CDK6
PPARA

MCODE1
MCODE2
MCODE3
MCODE4

created by
http://metascape.org
