# Supplementary material for: Exploring the mechanism of Alisma orientale for the treatment of pregnancy induced hypertension and potential hepato-nephrotoxicity by using network pharmacology, network toxicology, molecular docking and molecular dynamics simulation
Source: Front Pharmacol. 2022 Nov 15;13:1027112. doi: 10.3389/fphar.2022.1027112 (PMC9705790; doi:10.3389/fphar.2022.1027112)
Supplement: Supplementary file 4 [file DataSheet8.ZIP › Supplement Materials/GOKEGG/Treat/Metascape.zip!/Enrichment_GO/ColorByCluster.pdf]

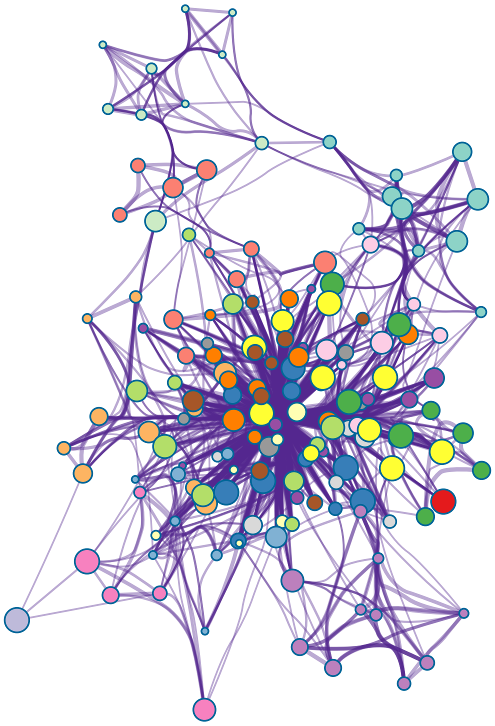

- Pathways in cancer
- response to hormone
- nuclear receptor activity
- Prostate cancer
- Endocrine resistance
- transmembrane receptor protein tyrosine kinase sign
- Prolactin signaling pathway
- regulation of defense response
- Chemical carcinogenesis - reactive oxygen species
- sex differentiation
- Diabetic cardiomyopathy
- regulation of system process
- response to radiation
- regulation of binding
- positive regulation of protein localization
- response to lipopolysaccharide
- Hepatocellular carcinoma
- regulation of apoptotic signaling pathway
- negative regulation of intracellular signal transductio
- regulation of hormone levels

created by
http://metascape.org
