# Supplementary material for: Exploring the mechanism of Alisma orientale for the treatment of pregnancy induced hypertension and potential hepato-nephrotoxicity by using network pharmacology, network toxicology, molecular docking and molecular dynamics simulation
Source: Front Pharmacol. 2022 Nov 15;13:1027112. doi: 10.3389/fphar.2022.1027112 (PMC9705790; doi:10.3389/fphar.2022.1027112)
Supplement: Supplementary file 4 [file DataSheet8.ZIP › Supplement Materials/GOKEGG/Treat/Metascape.zip!/Enrichment_heatmap/HeatmapSelectedGO.pdf]

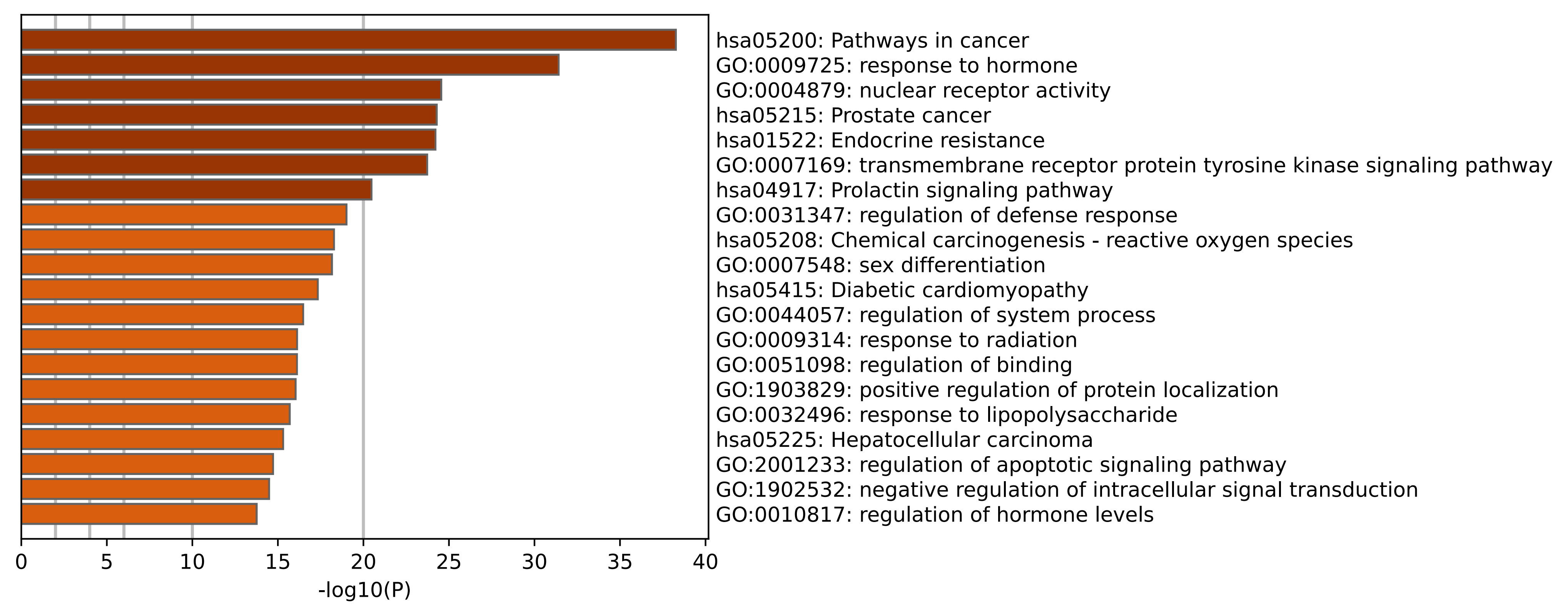
hsa05200: Pathways in cancer
GO:0009725: response to hormone
GO:0004879: nuclear receptor activity
hsa05215: Prostate cancer
hsa01522: Endocrine resistance
GO:0007169: transmembrane receptor protein tyrosine kinase signaling pathway
hsa04917: Prolactin signaling pathway
GO:0031347: regulation of defense response
hsa05208: Chemical carcinogenesis - reactive oxygen species
GO:0007548: sex differentiation
hsa05415: Diabetic cardiomyopathy
GO:0044057: regulation of system process
GO:0009314: response to radiation
GO:0051098: regulation of binding
GO:1903829: positive regulation of protein localization
GO:0032496: response to lipopolysaccharide
hsa05225: Hepatocellular carcinoma
GO:2001233: regulation of apoptotic signaling pathway
GO:1902532: negative regulation of intracellular signal transduction
GO:0010817: regulation of hormone levels
0
5
10
15
20
25
30
35
40
-log10(P)
