# Supplementary material for: Exploring the mechanism of Alisma orientale for the treatment of pregnancy induced hypertension and potential hepato-nephrotoxicity by using network pharmacology, network toxicology, molecular docking and molecular dynamics simulation
Source: Front Pharmacol. 2022 Nov 15;13:1027112. doi: 10.3389/fphar.2022.1027112 (PMC9705790; doi:10.3389/fphar.2022.1027112)
Supplement: Supplementary file 4 [file DataSheet8.ZIP › Supplement Materials/GOKEGG/Treat/Metascape.zip!/Enrichment_heatmap/HeatmapSelectedGOTop100.pdf]

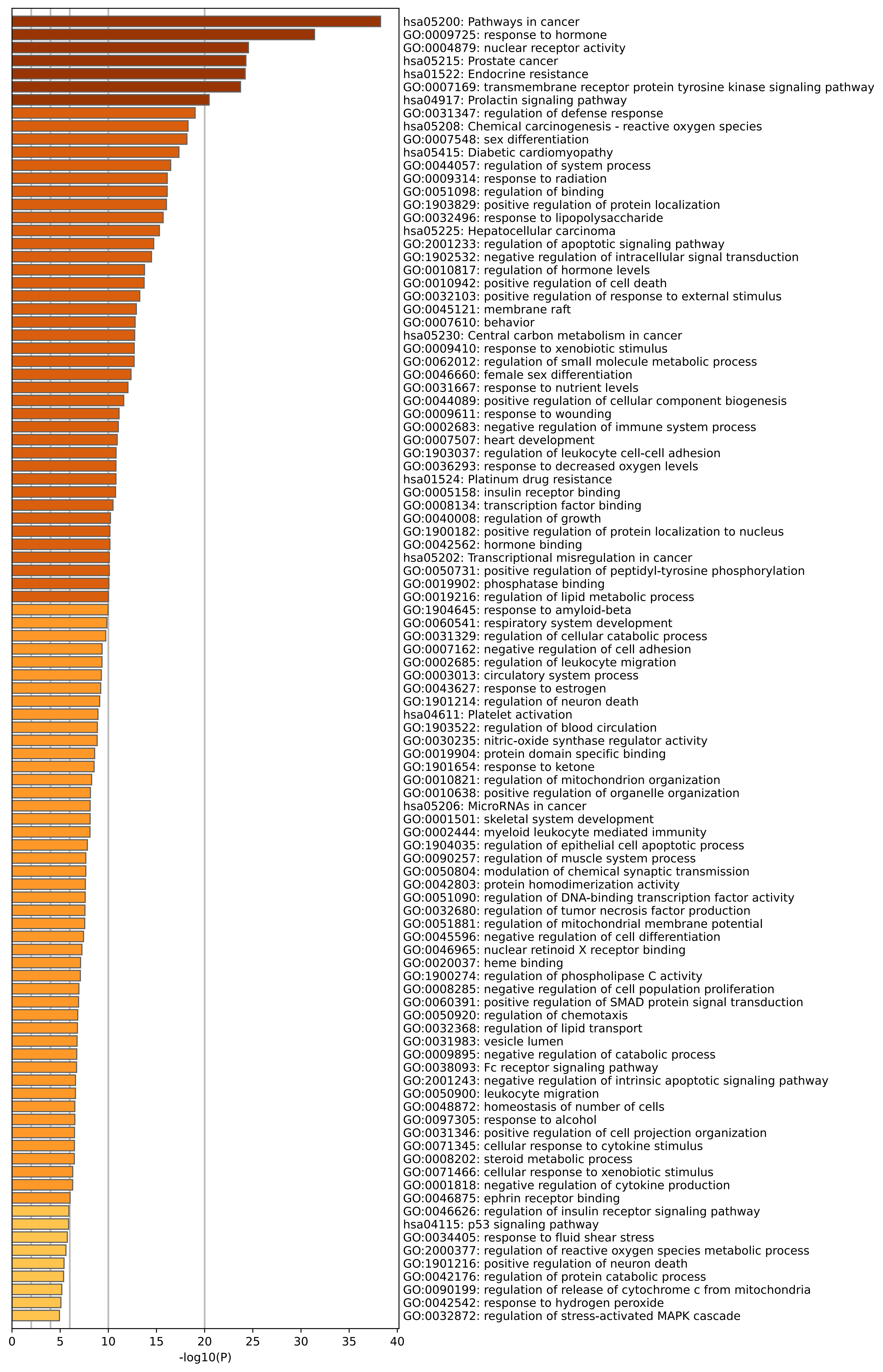
hsa05200: Pathways in cancer
GO:0009725: response to hormone
GO:0004879: nuclear receptor activity
hsa05215: Prostate cancer
hsa01522: Endocrine resistance
GO:0007169: transmembrane receptor protein tyrosine kinase signaling pathway
hsa04917: Prolactin signaling pathway
GO:0031347: regulation of defense response
hsa05208: Chemical carcinogenesis - reactive oxygen species
GO:0007548: sex differentiation
hsa05415: Diabetic cardiomyopathy
GO:0044057: regulation of system process
GO:0009314: response to radiation
GO:0051098: regulation of binding
GO:1903829: positive regulation of protein localization
GO:0032496: response to lipopolysaccharide
hsa05225: Hepatocellular carcinoma
GO:2001233: regulation of apoptotic signaling pathway
GO:1902532: negative regulation of intracellular signal transduction
GO:0010817: regulation of hormone levels
GO:0010942: positive regulation of cell death
GO:0032103: positive regulation of response to external stimulus
GO:0045121: membrane raft
GO:0007610: behavior
hsa05230: Central carbon metabolism in cancer
GO:0009410: response to xenobiotic stimulus
GO:0062012: regulation of small molecule metabolic process
GO:0046660: female sex differentiation
GO:0031667: response to nutrient levels
GO:0044089: positive regulation of cellular component biogenesis
GO:0009611: response to wounding
GO:0002683: negative regulation of immune system process
GO:0007507: heart development
GO:1903037: regulation of leukocyte cell-cell adhesion
GO:0036293: response to decreased oxygen levels
hsa01524: Platinum drug resistance
GO:0005158: insulin receptor binding
GO:0008134: transcription factor binding
GO:0040008: regulation of growth
GO:1900182: positive regulation of protein localization to nucleus
GO:0042562: hormone binding
hsa05202: Transcriptional misregulation in cancer
GO:0050731: positive regulation of peptidyl-tyrosine phosphorylation
GO:0019902: phosphatase binding
GO:0019216: regulation of lipid metabolic process
GO:1904645: response to amyloid-beta
GO:0060541: respiratory system development
GO:0031329: regulation of cellular catabolic process
GO:0007162: negative regulation of cell adhesion
GO:0002685: regulation of leukocyte migration
GO:0003013: circulatory system process
GO:0043627: response to estrogen
GO:1901214: regulation of neuron death
hsa04611: Platelet activation
GO:1903522: regulation of blood circulation
GO:0030235: nitric-oxide synthase regulator activity
GO:0019904: protein domain specific binding
GO:1901654: response to ketone
GO:0010821: regulation of mitochondrion organization
GO:0010638: positive regulation of organelle organization
hsa05206: MicroRNAs in cancer
GO:0001501: skeletal system development
GO:0002444: myeloid leukocyte mediated immunity
GO:1904035: regulation of epithelial cell apoptotic process
GO:0090257: regulation of muscle system process
GO:0050804: modulation of chemical synaptic transmission
GO:0042803: protein homodimerization activity
GO:0051090: regulation of DNA-binding transcription factor activity
GO:0032680: regulation of tumor necrosis factor production
GO:0051881: regulation of mitochondrial membrane potential
GO:0045596: negative regulation of cell differentiation
GO:0046965: nuclear retinoid X receptor binding
GO:0020037: heme binding
GO:1900274: regulation of phospholipase C activity
GO:0008285: negative regulation of cell population proliferation
GO:0060391: positive regulation of SMAD protein signal transduction
GO:0050920: regulation of chemotaxis
GO:0032368: regulation of lipid transport
GO:0031983: vesicle lumen
GO:0009895: negative regulation of catabolic process
GO:0038093: Fc receptor signaling pathway
GO:2001243: negative regulation of intrinsic apoptotic signaling pathway
GO:0050900: leukocyte migration
GO:0048872: homeostasis of number of cells
GO:0097305: response to alcohol
GO:0031346: positive regulation of cell projection organization
GO:0071345: cellular response to cytokine stimulus
GO:0008202: steroid metabolic process
GO:0071466: cellular response to xenobiotic stimulus
GO:0001818: negative regulation of cytokine production
GO:0046875: ephrin receptor binding
GO:0046626: regulation of insulin receptor signaling pathway
hsa04115: p53 signaling pathway
GO:0034405: response to fluid shear stress
GO:2000377: regulation of reactive oxygen species metabolic process
GO:1901216: positive regulation of neuron death
GO:0042176: regulation of protein catabolic process
GO:0090199: regulation of release of cytochrome c from mitochondria
GO:0042542: response to hydrogen peroxide
GO:0032872: regulation of stress-activated MAPK cascade
0
5
10
15
20
25
30
35
40
-log10(P)
