# Supplementary material for: Exploring the mechanism of Alisma orientale for the treatment of pregnancy induced hypertension and potential hepato-nephrotoxicity by using network pharmacology, network toxicology, molecular docking and molecular dynamics simulation
Source: Front Pharmacol. 2022 Nov 15;13:1027112. doi: 10.3389/fphar.2022.1027112 (PMC9705790; doi:10.3389/fphar.2022.1027112)
Supplement: Supplementary file 4 [file DataSheet8.ZIP › Supplement Materials/GOKEGG/Treat/Metascape.zip!/Enrichment_PPI/MyList_MCODE_ALL_PPIColorByCluster.pdf]

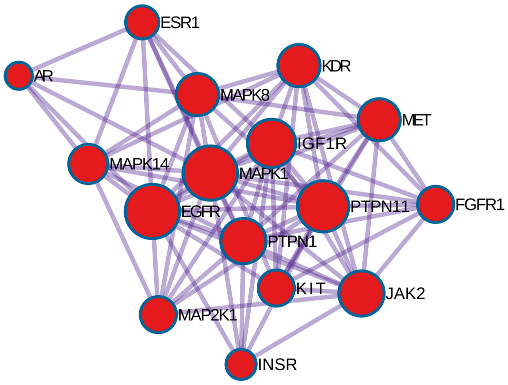
ESR1
AR
KDR
MAPK8
MET
IGF1R
MAPK14
MAPK1
FGFR1
EGFR
PTPN11
PTPN1
KIT
JAK2
MAP2K1
INSR

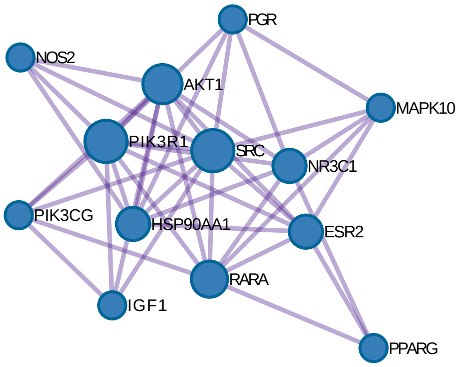
PGR
NOS2
AKT1
MAPK10
PIK3R1
SRC
NR3C1
PIK3CG
HSP90AA1
ESR2
RARA
IGF1
PPARG

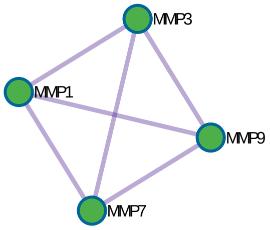
MMP3
MMP1
MMP9
MMP7

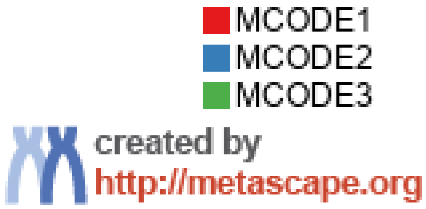
MCODE1
MCODE2
MCODE3
created by
http://metascape.org
