# Supplementary material for: Exploring the mechanism of Alisma orientale for the treatment of pregnancy induced hypertension and potential hepato-nephrotoxicity by using network pharmacology, network toxicology, molecular docking and molecular dynamics simulation
Source: Front Pharmacol. 2022 Nov 15;13:1027112. doi: 10.3389/fphar.2022.1027112 (PMC9705790; doi:10.3389/fphar.2022.1027112)
Supplement: Supplementary file 5 [file DataSheet9.ZIP › Revise Nephrotoxicity/all.tz_808bp4.zip!/Enrichment_GO/ColorByCluster.pdf]

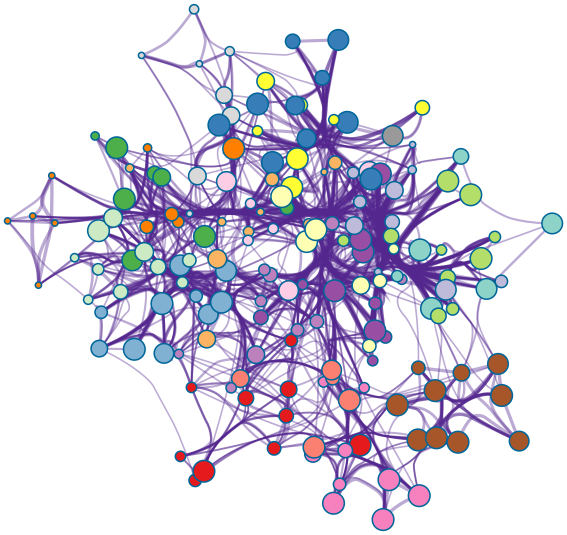

- Pathways in cancer
- response to hormone
- cellular response to nitrogen compound
- positive regulation of protein localization
- response to steroid hormone
- response to nutrient levels
- positive regulation of protein phosphorylation
- protein phosphorylation
- Chemical carcinogenesis - receptor activation
- regulation of defense response
- response to bacterium
- positive regulation of lipid metabolic process
- enzyme-linked receptor protein signaling pathway
- positive regulation of cell death
- regulation of miRNA transcription
- regulation of secretion
- response to oxygen levels
- multi-organism reproductive process
- IL-17 signaling pathway
- response to inorganic substance

created by
http://metascape.org
