# Supplementary material for: Exploring the mechanism of Alisma orientale for the treatment of pregnancy induced hypertension and potential hepato-nephrotoxicity by using network pharmacology, network toxicology, molecular docking and molecular dynamics simulation
Source: Front Pharmacol. 2022 Nov 15;13:1027112. doi: 10.3389/fphar.2022.1027112 (PMC9705790; doi:10.3389/fphar.2022.1027112)
Supplement: Supplementary file 5 [file DataSheet9.ZIP › Revise Nephrotoxicity/all.tz_808bp4.zip!/Enrichment_GO/ColorByPValue.pdf]

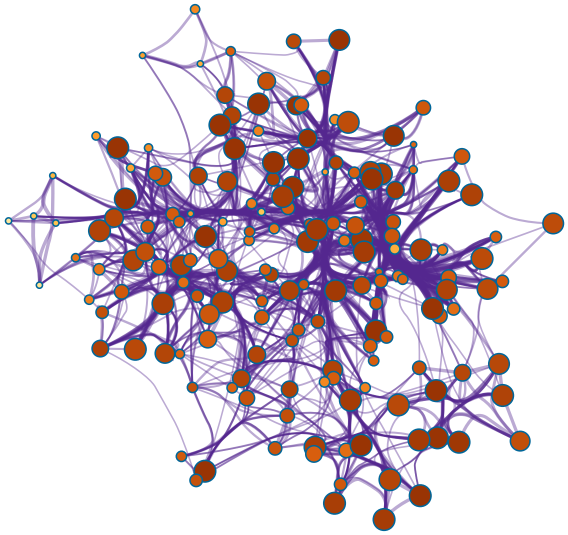

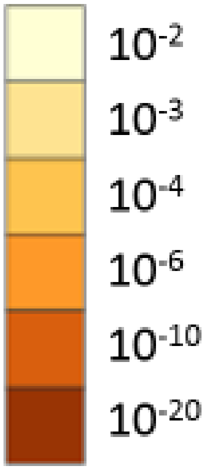

created by
http://metascape.org
