# Supplementary material for: Exploring the mechanism of Alisma orientale for the treatment of pregnancy induced hypertension and potential hepato-nephrotoxicity by using network pharmacology, network toxicology, molecular docking and molecular dynamics simulation
Source: Front Pharmacol. 2022 Nov 15;13:1027112. doi: 10.3389/fphar.2022.1027112 (PMC9705790; doi:10.3389/fphar.2022.1027112)
Supplement: Supplementary file 5 [file DataSheet9.ZIP › Revise Nephrotoxicity/all.tz_808bp4.zip!/Enrichment_heatmap/HeatmapSelectedGO.pdf]

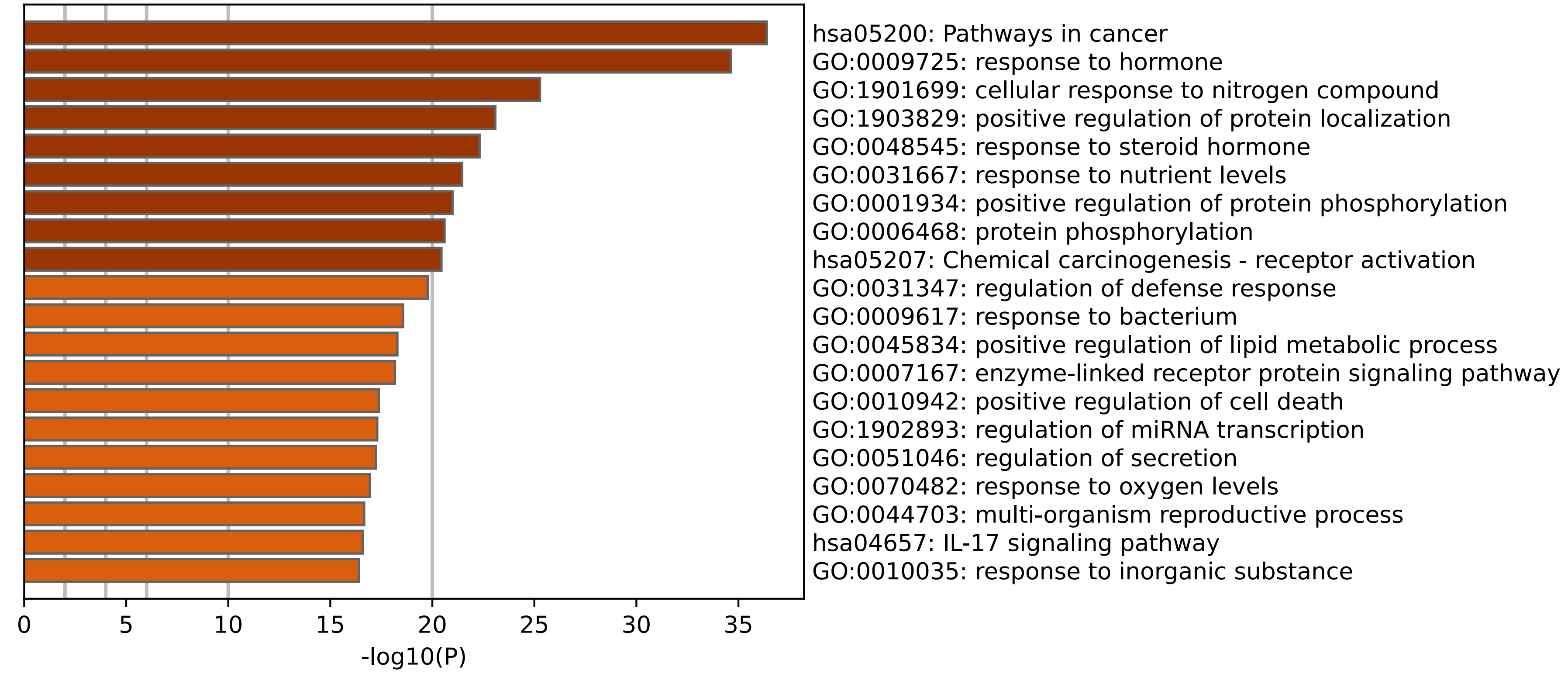
hsa05200: Pathways in cancer
GO:0009725: response to hormone
GO:1901699: cellular response to nitrogen compound
GO:1903829: positive regulation of protein localization
GO:0048545: response to steroid hormone
GO:0031667: response to nutrient levels
GO:0001934: positive regulation of protein phosphorylation
GO:0006468: protein phosphorylation
hsa05207: Chemical carcinogenesis - receptor activation
GO:0031347: regulation of defense response
GO:0009617: response to bacterium
GO:0045834: positive regulation of lipid metabolic process
GO:0007167: enzyme-linked receptor protein signaling pathway
GO:0010942: positive regulation of cell death
GO:1902893: regulation of miRNA transcription
GO:0051046: regulation of secretion
GO:0070482: response to oxygen levels
GO:0044703: multi-organism reproductive process
hsa04657: IL-17 signaling pathway
GO:0010035: response to inorganic substance
0
5
10
15
20
25
30
35
-log10(P)
