# Supplementary material for: Exploring the mechanism of Alisma orientale for the treatment of pregnancy induced hypertension and potential hepato-nephrotoxicity by using network pharmacology, network toxicology, molecular docking and molecular dynamics simulation
Source: Front Pharmacol. 2022 Nov 15;13:1027112. doi: 10.3389/fphar.2022.1027112 (PMC9705790; doi:10.3389/fphar.2022.1027112)
Supplement: Supplementary file 5 [file DataSheet9.ZIP › Revise Nephrotoxicity/all.tz_808bp4.zip!/Enrichment_heatmap/HeatmapSelectedGOTop100.pdf]

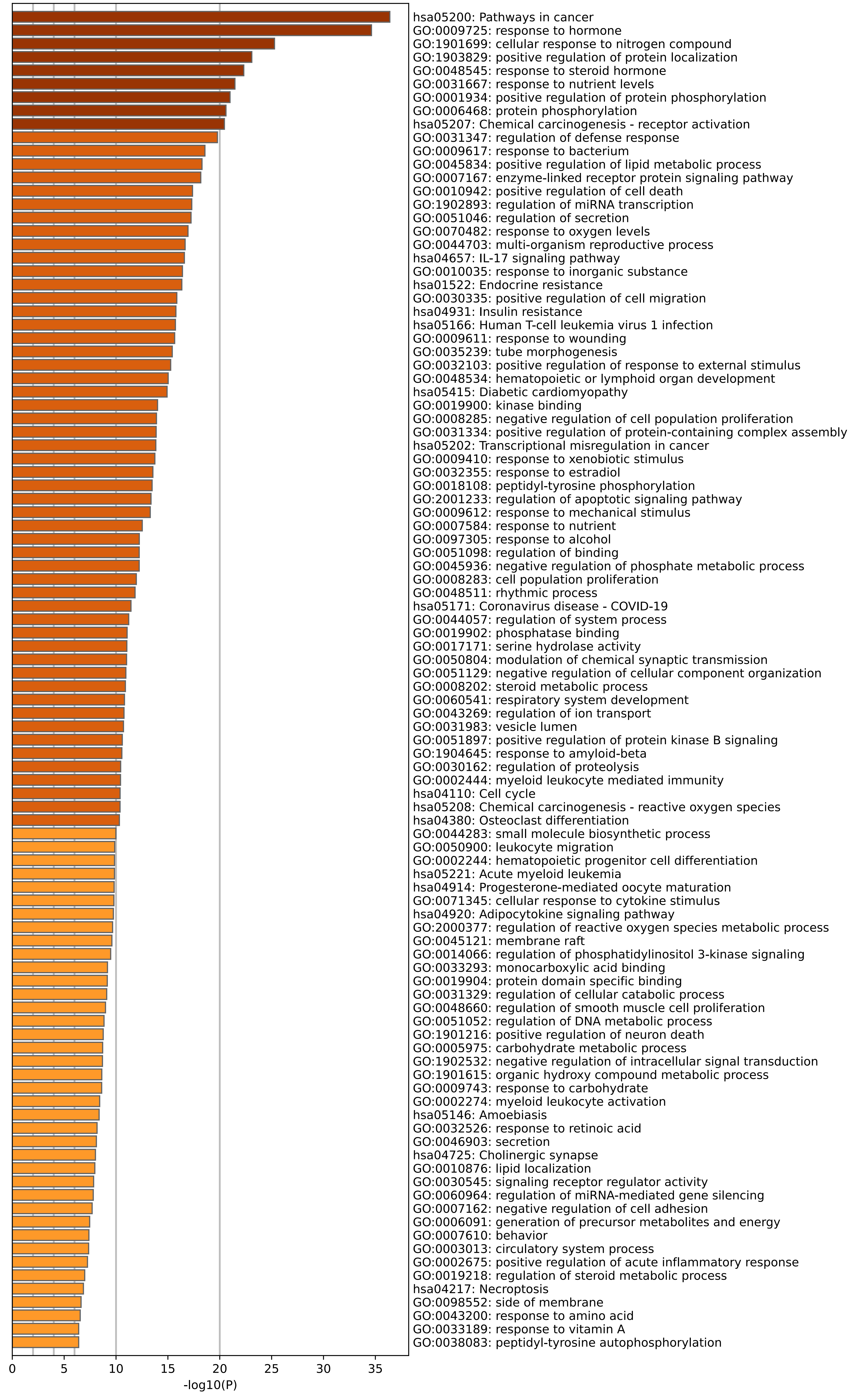
hsa05200: Pathways in cancer
GO:0009725: response to hormone
GO:1901699: cellular response to nitrogen compound
GO:1903829: positive regulation of protein localization
GO:0048545: response to steroid hormone
GO:0031667: response to nutrient levels
GO:0001934: positive regulation of protein phosphorylation
GO:0006468: protein phosphorylation
hsa05207: Chemical carcinogenesis - receptor activation
GO:0031347: regulation of defense response
GO:0009617: response to bacterium
GO:0045834: positive regulation of lipid metabolic process
GO:0007167: enzyme-linked receptor protein signaling pathway
GO:0010942: positive regulation of cell death
GO:1902893: regulation of miRNA transcription
GO:0051046: regulation of secretion
GO:0070482: response to oxygen levels
GO:0044703: multi-organism reproductive process
hsa04657: IL-17 signaling pathway
GO:0010035: response to inorganic substance
hsa01522: Endocrine resistance
GO:0030335: positive regulation of cell migration
hsa04931: Insulin resistance
hsa05166: Human T-cell leukemia virus 1 infection
GO:0009611: response to wounding
GO:0035239: tube morphogenesis
GO:0032103: positive regulation of response to external stimulus
GO:0048534: hematopoietic or lymphoid organ development
hsa05415: Diabetic cardiomyopathy
GO:0019900: kinase binding
GO:0008285: negative regulation of cell population proliferation
GO:0031334: positive regulation of protein-containing complex assembly
hsa05202: Transcriptional misregulation in cancer
GO:0009410: response to xenobiotic stimulus
GO:0032355: response to estradiol
GO:0018108: peptidyl-tyrosine phosphorylation
GO:2001233: regulation of apoptotic signaling pathway
GO:0009612: response to mechanical stimulus
GO:0007584: response to nutrient
GO:0097305: response to alcohol
GO:0051098: regulation of binding
GO:0045936: negative regulation of phosphate metabolic process
GO:0008283: cell population proliferation
GO:0048511: rhythmic process
hsa05171: Coronavirus disease - COVID-19
GO:0044057: regulation of system process
GO:0019902: phosphatase binding
GO:0017171: serine hydrolase activity
GO:0050804: modulation of chemical synaptic transmission
GO:0051129: negative regulation of cellular component organization
GO:0008202: steroid metabolic process
GO:0060541: respiratory system development
GO:0043269: regulation of ion transport
GO:0031983: vesicle lumen
GO:0051897: positive regulation of protein kinase B signaling
GO:1904645: response to amyloid-beta
GO:0030162: regulation of proteolysis
GO:0002444: myeloid leukocyte mediated immunity
hsa04110: Cell cycle
hsa05208: Chemical carcinogenesis - reactive oxygen species
hsa04380: Osteoclast differentiation
GO:0044283: small molecule biosynthetic process
GO:0050900: leukocyte migration
GO:0002244: hematopoietic progenitor cell differentiation
hsa05221: Acute myeloid leukemia
hsa04914: Progesterone-mediated oocyte maturation
GO:0071345: cellular response to cytokine stimulus
hsa04920: Adipocytokine signaling pathway
GO:2000377: regulation of reactive oxygen species metabolic process
GO:0045121: membrane raft
GO:0014066: regulation of phosphatidylinositol 3-kinase signaling
GO:0033293: monocarboxylic acid binding
GO:0019904: protein domain specific binding
GO:0031329: regulation of cellular catabolic process
GO:0048660: regulation of smooth muscle cell proliferation
GO:0051052: regulation of DNA metabolic process
GO:1901216: positive regulation of neuron death
GO:0005975: carbohydrate metabolic process
GO:1902532: negative regulation of intracellular signal transduction
GO:1901615: organic hydroxy compound metabolic process
GO:0009743: response to carbohydrate
GO:0002274: myeloid leukocyte activation
hsa05146: Amoebiasis
GO:0032526: response to retinoic acid
GO:0046903: secretion
hsa04725: Cholinergic synapse
GO:0010876: lipid localization
GO:0030545: signaling receptor regulator activity
GO:0060964: regulation of miRNA-mediated gene silencing
GO:0007162: negative regulation of cell adhesion
GO:0006091: generation of precursor metabolites and energy
GO:0007610: behavior
GO:0003013: circulatory system process
GO:0002675: positive regulation of acute inflammatory response
GO:0019218: regulation of steroid metabolic process
hsa04217: Necroptosis
GO:0098552: side of membrane
GO:0043200: response to amino acid
GO:0033189: response to vitamin A
GO:0038083: peptidyl-tyrosine autophosphorylation
0
5
10
15
20
25
30
35
-log10(P)
