# Supplementary material for: Exploring the mechanism of Alisma orientale for the treatment of pregnancy induced hypertension and potential hepato-nephrotoxicity by using network pharmacology, network toxicology, molecular docking and molecular dynamics simulation
Source: Front Pharmacol. 2022 Nov 15;13:1027112. doi: 10.3389/fphar.2022.1027112 (PMC9705790; doi:10.3389/fphar.2022.1027112)
Supplement: Supplementary file 5 [file DataSheet9.ZIP › Revise Nephrotoxicity/all.tz_808bp4.zip!/Enrichment_PPI/MyList_MCODE_ALL_PPIColorByCluster.pdf]

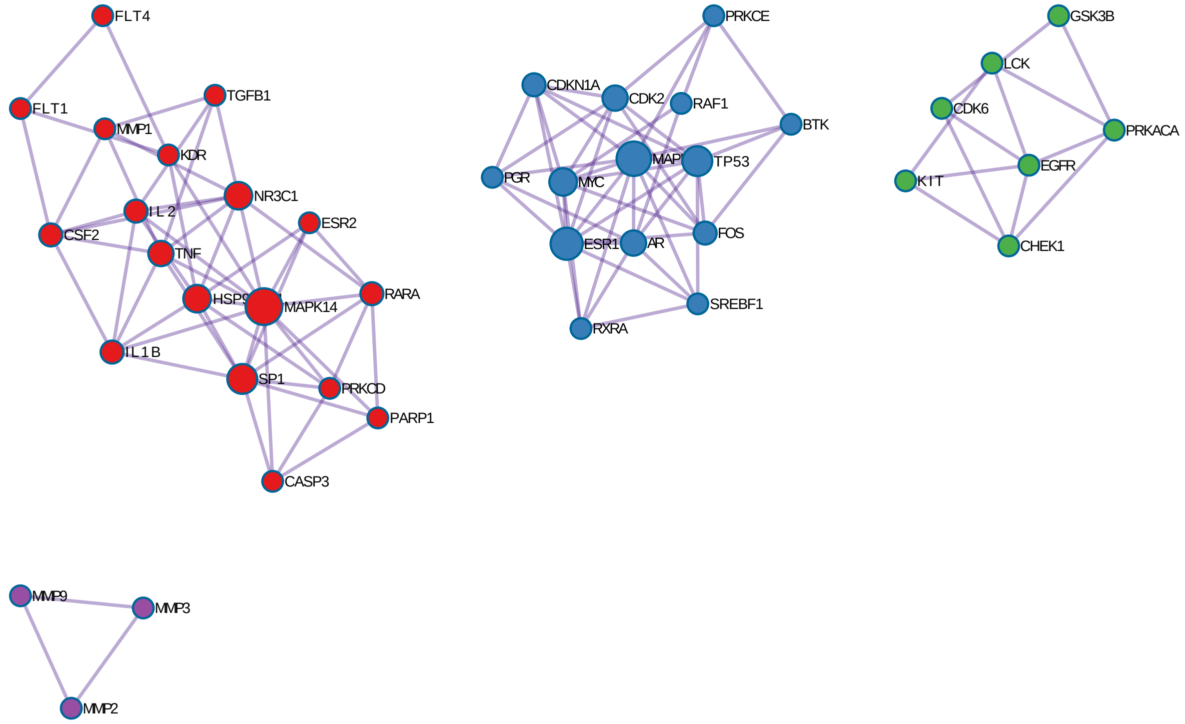
FLT4
FLT1
TGFB1
MMP1
KDR
NR3C1
IL2
ESR2
CSF2
TNF
RARA
MAPK14
IL1B
SP1
PRKCD
PARP1
CASP3
PRKCE
CDKN1A
CDK2
RAF1
BTK
PGR
MYC
TP53
ESR1
AR
FOS
SREBF1
RXRA
GSK3B
LCK
CDK6
PRKACA
KIT
EGFR
CHEK1
MMP9
MMP3
MMP2

MCODE1
MCODE2
MCODE3
MCODE4

created by
http://metascape.org
