# Supplementary material for: Exploring the mechanism of Alisma orientale for the treatment of pregnancy induced hypertension and potential hepato-nephrotoxicity by using network pharmacology, network toxicology, molecular docking and molecular dynamics simulation
Source: Front Pharmacol. 2022 Nov 15;13:1027112. doi: 10.3389/fphar.2022.1027112 (PMC9705790; doi:10.3389/fphar.2022.1027112)
Supplement: Supplementary file 5 [file DataSheet9.ZIP › Revise Nephrotoxicity/all.tz_808bp4.zip!/AnalysisReport.html]

## Metascape Gene List Analysis Report

metascape.org^1^

### Bar Graph Summary

Figure 1. Bar graph of enriched terms across input gene lists, colored by p-values.

Metascape only visualizes the top 20 clusters. Up to 100 enriched clusters can be viewed here.

The top-level Gene Ontology biological processes can be viewed here.

### Gene Lists

User-provided gene identifiers are first converted into their corresponding H. sapiens Entrez gene IDs using the latest version of the database (last updated on 2022-10-01). If multiple identifiers correspond to the same Entrez gene ID, they will be considered as a single Entrez gene ID in downstream analyses. The gene lists are summarized in Table 1.

Table 1. Statistics of input gene lists.

| Name | Total | Unique |
| --- | --- | --- |
| MyList | 111 | 111 |

### Pathway and Process Enrichment Analysis

For each given gene list, pathway and process enrichment analysis has been carried out with the following ontology sources: GO Biological Processes, GO Cellular Components, GO Molecular Functions and KEGG Pathway. All genes in the genome have been used as the enrichment background. Terms with a p-value < 0.01, a minimum count of 3, and an enrichment factor > 1.5 (the enrichment factor is the ratio between the observed counts and the counts expected by chance) are collected and grouped into clusters based on their membership similarities. More specifically, p-values are calculated based on the cumulative hypergeometric distribution^2^, and q-values are calculated using the Benjamini-Hochberg procedure to account for multiple testings^3^. Kappa scores^4^ are used as the similarity metric when performing hierarchical clustering on the enriched terms, and sub-trees with a similarity of > 0.3 are considered a cluster. The most statistically significant term within a cluster is chosen to represent the cluster.

Table 2. Top 20 clusters with their representative enriched terms (one per cluster). "Count" is the number of genes in the user-provided lists with membership in the given ontology term. "%" is the percentage of all of the user-provided genes that are found in the given ontology term (only input genes with at least one ontology term annotation are included in the calculation). "Log10(P)" is the p-value in log base 10. "Log10(q)" is the multi-test adjusted p-value in log base 10.

| GO | Category | Description | Count | % | Log10(P) | Log10(q) |
| --- | --- | --- | --- | --- | --- | --- |
| hsa05200 | KEGG Pathway | Pathways in cancer | 37 | 33.33 | -36.39 | -32.04 |
| GO:0009725 | GO Biological Processes | response to hormone | 40 | 36.04 | -34.63 | -30.57 |
| GO:1901699 | GO Biological Processes | cellular response to nitrogen compound | 31 | 27.93 | -25.28 | -21.77 |
| GO:1903829 | GO Biological Processes | positive regulation of protein localization | 26 | 23.42 | -23.09 | -19.78 |
| GO:0048545 | GO Biological Processes | response to steroid hormone | 22 | 19.82 | -22.32 | -19.11 |
| GO:0031667 | GO Biological Processes | response to nutrient levels | 25 | 22.52 | -21.46 | -18.28 |
| GO:0001934 | GO Biological Processes | positive regulation of protein phosphorylation | 29 | 26.13 | -20.99 | -17.84 |
| GO:0006468 | GO Biological Processes | protein phosphorylation | 28 | 25.23 | -20.60 | -17.50 |
| hsa05207 | KEGG Pathway | Chemical carcinogenesis - receptor activation | 19 | 17.12 | -20.45 | -17.37 |
| GO:0031347 | GO Biological Processes | regulation of defense response | 27 | 24.32 | -19.77 | -16.74 |
| GO:0009617 | GO Biological Processes | response to bacterium | 27 | 24.32 | -18.57 | -15.61 |
| GO:0045834 | GO Biological Processes | positive regulation of lipid metabolic process | 16 | 14.41 | -18.29 | -15.38 |
| GO:0007167 | GO Biological Processes | enzyme-linked receptor protein signaling pathway | 25 | 22.52 | -18.17 | -15.31 |
| GO:0010942 | GO Biological Processes | positive regulation of cell death | 24 | 21.62 | -17.37 | -14.58 |
| GO:1902893 | GO Biological Processes | regulation of miRNA transcription | 12 | 10.81 | -17.30 | -14.53 |
| GO:0051046 | GO Biological Processes | regulation of secretion | 24 | 21.62 | -17.23 | -14.47 |
| GO:0070482 | GO Biological Processes | response to oxygen levels | 19 | 17.12 | -16.94 | -14.23 |
| GO:0044703 | GO Biological Processes | multi-organism reproductive process | 16 | 14.41 | -16.66 | -13.99 |
| hsa04657 | KEGG Pathway | IL-17 signaling pathway | 13 | 11.71 | -16.59 | -13.92 |
| GO:0010035 | GO Biological Processes | response to inorganic substance | 22 | 19.82 | -16.40 | -13.77 |

To further capture the relationships between the terms, a subset of enriched terms have been selected and rendered as a network plot, where terms with a similarity > 0.3 are connected by edges. We select the terms with the best p-values from each of the 20 clusters, with the constraint that there are no more than 15 terms per cluster and no more than 250 terms in total. The network is visualized using Cytoscape^5^, where each node represents an enriched term and is colored first by its cluster ID (Figure 2.a) and then by its p-value (Figure 2.b). These networks can be interactively viewed in Cytoscape through the .cys files (contained in the Zip package, which also contains a publication-quality version as a PDF) or within a browser by clicking on the web icon. For clarity, term labels are only shown for one term per cluster, so it is recommended to use Cytoscape or a browser to visualize the network in order to inspect all node labels. We can also export the network into a PDF file within Cytoscape, and then edit the labels using Adobe Illustrator for publication purposes. To switch off all labels, delete the "Label" mapping under the "Style" tab within Cytoscape, and then export the network view.

Figure 2. Network of enriched terms: (a) colored by cluster ID, where nodes that share the same cluster ID are typically close to each other; (b) colored by p-value, where terms containing more genes tend to have a more significant p-value.

|  |  |
| --- | --- |
|  |  |
|  |  |

### Protein-protein Interaction Enrichment Analysis

For each given gene list, protein-protein interaction enrichment analysis has been carried out with the following databases: STRING^6^, BioGrid^7^, OmniPath^8^, InWeb\_IM^9^.Only physical interactions in STRING (physical score > 0.132) and BioGrid are used (details). The resultant network contains the subset of proteins that form physical interactions with at least one other member in the list. If the network contains between 3 and 500 proteins, the Molecular Complex Detection (MCODE) algorithm^10^ has been applied to identify densely connected network components. The MCODE networks identified for individual gene lists have been gathered and are shown in Figure 3.

Pathway and process enrichment analysis has been applied to each MCODE component independently, and the three best-scoring terms by p-value have been retained as the functional description of the corresponding components, shown in the tables underneath corresponding network plots within Figure 3.

Figure 3. Protein-protein interaction network and MCODE components identified in the gene lists.

| GO | Description | Log10(P) |
| --- | --- | --- |
| hsa05200 | Pathways in cancer | -36.7 |
| GO:0009725 | response to hormone | -32.3 |
| GO:0071407 | cellular response to organic cyclic compound | -30.0 |

| Color | MCODE | GO | Description | Log10(P) |
| --- | --- | --- | --- | --- |
|  | MCODE\_1 | GO:0009725 | response to hormone | -13.2 |
|  | MCODE\_1 | hsa04657 | IL-17 signaling pathway | -13.2 |
|  | MCODE\_1 | GO:0030097 | hemopoiesis | -12.1 |
|  | MCODE\_2 | hsa05200 | Pathways in cancer | -14.6 |
|  | MCODE\_2 | GO:0001221 | transcription coregulator binding | -13.7 |
|  | MCODE\_2 | hsa05224 | Breast cancer | -12.7 |
|  | MCODE\_3 | GO:0004672 | protein kinase activity | -12.1 |
|  | MCODE\_3 | GO:0016773 | phosphotransferase activity, alcohol group as acceptor | -11.6 |
|  | MCODE\_3 | GO:0006468 | protein phosphorylation | -11.4 |
|  | MCODE\_4 | GO:0071492 | cellular response to UV-A | -10.4 |
|  | MCODE\_4 | GO:0070141 | response to UV-A | -10.1 |
|  | MCODE\_4 | GO:0030574 | collagen catabolic process | -8.6 |

### Reference

1. Zhou et al., Metascape provides a biologist-oriented resource for the analysis of systems-level datasets. Nature Communications (2019) 10(1):1523.
2. Zar, J.H. Biostatistical Analysis 1999 4th edn., NJ Prentice Hall, pp. 523
3. Hochberg Y., Benjamini Y. More powerful procedures for multiple significance testing. Statistics in Medicine (1990) 9:811-818.
4. Cohen, J. A coefficient of agreement for nominal scales. Educ. Psychol. Meas. (1960) 20:27-46.
5. Shannon P. et al., Cytoscape: a software environment for integrated models of biomolecular interaction networks. Genome Res (2003) 11:2498-2504.
6. Szklarczyk D. et al. STRING v11: protein-protein association networks with increased coverage, supporting functional discovery in genome-wide experimental datasets. Nucleic Acids Res. (2019) 47:D607-613.
7. Stark C. et al. BioGRID: a general repository for interaction datasets. Nucleic Acids Res. (2006) 34:D535-539.
8. Turei D. et al. A scored human protein-protein interaction network to catalyze genomic interpretation. Nat. Methods. (2016) 13:966-967.
9. Li T. et al. A scored human protein-protein interaction network to catalyze genomic interpretation. Nat. Methods. (2017) 14:61-64.
10. Bader, G.D. et al. An automated method for finding molecular complexes in large protein interaction networks. BMC bioinformatics (2003) 4:2.
