# Supplementary material for: Exploring the mechanism of Alisma orientale for the treatment of pregnancy induced hypertension and potential hepato-nephrotoxicity by using network pharmacology, network toxicology, molecular docking and molecular dynamics simulation
Source: Front Pharmacol. 2022 Nov 15;13:1027112. doi: 10.3389/fphar.2022.1027112 (PMC9705790; doi:10.3389/fphar.2022.1027112)
Supplement: Supplementary file 4 [file DataSheet8.ZIP › Supplement Materials/Molecular Docking/Toxic/CASP3472.zip!/./ligand.rtf]

* ----

* Built RTF for tmpgNDYKx.mol2

*	by user swissdock	Fri Jul 1 16:21:50 CEST 2022

* ----

*

22	0

MASS	201 CB	12.011000

MASS	202 C=O	12.011000

MASS	203 O=C	15.999400

MASS	204 OR	15.999400

MASS	205 CR	12.011000

MASS	206 HCMM	1.007940

MASS	207 HOCC	1.007940

AUTOGENERATE ANGLES DIHE

DEFA FIRS NONE LAST NONE

RESI LIG -0.000

GROUP

ATOM C	CB	0.0825

ATOM C1	CB	-0.1500

ATOM C2	CB	0.0825

ATOM C3	CB	-0.1500

ATOM C4	CB	0.0862

ATOM C6	CB	0.0862

ATOM C7	CB	-0.1500

ATOM C8	CB	-0.1435

ATOM C9	CB	-0.1500

ATOM C10 CB	0.0825

ATOM C11 CB	0.0862

ATOM C13 CB	0.0862

ATOM C5	C=O	0.3976

ATOM O	O=C -0.5700

ATOM C12 C=O	0.3976

ATOM O1	O=C -0.5700

ATOM O2	OR	-0.5325

ATOM O3	OR	-0.5325

ATOM O4	OR	-0.5325

ATOM C14 CR	0.1435

ATOM H	HCMM 0.1500

ATOM H1	HCMM 0.1500

ATOM H2	HCMM 0.1500

ATOM H3	HCMM 0.1500

ATOM H4	HOCC 0.4500

ATOM H5	HOCC 0.4500

ATOM H6	HOCC 0.4500

ATOM H7	HCMM 0.0000

ATOM H8	HCMM 0.0000

ATOM H9	HCMM 0.0000

BOND C	C1

BOND C	C13

BOND C	O4

BOND C1	C2

BOND C1	H

BOND C2	C3

BOND C2	O3

BOND C3	C4

BOND C3	H1

BOND C4	C5

BOND C4	C13

BOND C5	O

BOND C5	C6

BOND C6	C7

BOND C6	C11

BOND C7	C8

BOND C7	H2

BOND C8	C9

BOND C8	C14

BOND C9	C10

BOND C9	H3

BOND C10 C11

BOND C10 O2

BOND C11 C12

BOND C12 O1

BOND C12 C13

BOND O2	H4

BOND O3	H5

BOND O4	H6

BOND C14 H7

BOND C14 H8

BOND C14 H9

IMPH C	C1	C13 O4

IMPH C1	C2	C	H

IMPH C2	C3	C1	O3

IMPH C3	C4	C2	H1

IMPH C4	C13 C3	C5

IMPH C5	C6	C4	O

IMPH C6	C11 C5	C7

IMPH C7	C8	C6	H2

IMPH C8	C9	C7	C14

IMPH C9	C10 C8	H3

IMPH C11 C10 C6	C12

IMPH C12 C13 C11 O1

IMPH C10 C11 C9	O2

IMPH C14 H7	C8	H8

IMPH C14 H7	C8	H9

IC C	C1	C2	C3	1.48	120.71	-0.05 119.61 1.48

IC C	C1	C2	O3	1.48	120.71 179.93 121.43 1.40

IC C	C13	C4	C3	1.44	119.73	-0.09 119.86 1.42

IC C	C13	C4	C5	1.44	119.73 179.92 119.22 1.51

IC C	C13	C12 C11	1.44	121.63 179.71 121.15 1.51

IC C	C13	C12 O1	1.44	121.63	-0.26 119.40 1.22

IC C1	C	C13 C4	1.48	119.48	0.08 119.73 1.48

IC C1	C	C13 C12	1.48	119.48 -179.94 121.63 1.51

IC C1	C	O4	H6	1.48	118.82 -179.86 114.30 0.95

IC C1	C2	C3	C4	1.41	119.61	0.04 120.61 1.42

IC C1	C2	C3	H1	1.41	119.61 -179.93 118.96 1.08

IC C1	C2	O3	H5	1.41	121.43	-0.46 114.62 0.95

IC C2	C1	C	C13	1.41	120.71	-0.02 119.48 1.44

IC C2	C1	C	O4	1.41	120.71 -179.99 118.82 1.40

IC C2	C3	C4	C13	1.48	120.61	0.03 119.86 1.48

IC C2	C3	C4	C5	1.48	120.61 -179.98 120.92 1.51

IC C3	C2	C1	H	1.48	119.61 180.00 120.75 1.08

IC C3	C2	O3	H5	1.48	118.96 179.52 114.62 0.95

IC C3	C4	C13 C12	1.42	119.86 179.94 118.64 1.51

IC C3	C4	C5	C6	1.42	120.92 -179.60 120.48 1.51

IC C3	C4	C5	O	1.42	120.92	0.32 119.73 1.22

IC C4	C3	C2	O3	1.42	120.61 -179.93 118.96 1.40

IC C4	C13	C	O4	1.48	119.73 -179.94 121.71 1.40

IC C4	C13	C12 C11	1.48	118.64	-0.31 121.15 1.51

IC C4	C13	C12 O1	1.48	118.64 179.72 119.40 1.22

IC C4	C5	C6	C7	1.51	120.48 179.58 119.69 1.48

IC C4	C5	C6	C11	1.51	120.48	-0.36 120.53 1.42

IC C6	C7	C8	C9	1.48	120.52	-0.09 119.44 1.48

IC C6	C7	C8	C14	1.48	120.52 179.91 119.73 1.53

IC C6	C11	C10 C9	1.42	119.68	-0.01 119.67 1.40

IC C6	C11	C10 O2	1.42	119.68 179.92 121.49 1.40

IC C6	C11	C12 C13	1.42	119.98	0.35 121.15 1.51

IC C6	C11	C12 O1	1.42	119.98 -179.68 119.46 1.22

IC C6	C5	C4	C13	1.51	120.48	0.39 119.22 1.48

IC C7	C6	C11 C10	1.48	119.78	0.01 119.68 1.49

IC C7	C6	C11 C12	1.48	119.78 -179.95 119.98 1.51

IC C7	C6	C5	O	1.48	119.69	-0.34 119.79 1.22

IC C7	C8	C9	C10	1.40	119.44	0.09 120.92 1.40

IC C7	C8	C9	H3	1.40	119.44 -179.97 120.18 1.08

IC C7	C8	C14 H7	1.40	119.73 -175.51 111.47 1.10

IC C7	C8	C14 H8	1.40	119.73 -55.18 109.48 1.10

IC C7	C8	C14 H9	1.40	119.73	64.22 109.15 1.10

IC C8	C7	C6	C11	1.40	120.52	0.04 119.78 1.42

IC C8	C7	C6	C5	1.40	120.52 -179.90 119.69 1.51

IC C8	C9	C10 C11	1.48	120.92	-0.04 119.67 1.49

IC C8	C9	C10 O2	1.48	120.92 -179.97 118.85 1.40

IC C9	C8	C7	H2	1.48	119.44 179.86 119.72 1.08

IC C9	C8	C14 H7	1.48	120.83	4.49 111.47 1.10

IC C9	C8	C14 H8	1.48	120.83 124.82 109.48 1.10

IC C9	C8	C14 H9	1.48	120.83 -115.77 109.15 1.10

IC C9	C10	C11 C12	1.40	119.67 179.95 120.34 1.51

IC C9	C10	O2	H4	1.40	118.85 179.89 113.80 0.95

IC C10 C9	C8	C14	1.40	120.92 -179.91 120.83 1.53

IC C10 C11	C6	C5	1.49	119.68 179.95 120.53 1.51

IC C10 C11	C12 C13	1.49	120.34 -179.61 121.15 1.51

IC C10 C11	C12 O1	1.49	120.34	0.36 119.46 1.22

IC C11 C6	C7	H2	1.42	119.78 -179.91 119.76 1.08

IC C11 C6	C5	O	1.42	120.53 179.72 119.79 1.22

IC C11 C10	C9	H3	1.49	119.67 -179.98 118.89 1.08

IC C11 C10	O2	H4	1.49	121.49	-0.04 113.80 0.95

IC C13 C	C1	H	1.44	119.48 179.94 118.55 1.08

IC C13 C	O4	H6	1.44	121.71	0.16 114.30 0.95

IC C13 C4	C3	H1	1.48	119.86 -180.00 120.43 1.08

IC C13 C4	C5	O	1.48	119.22 -179.69 119.73 1.22

IC C5	C4	C3	H1	1.51	120.92	-0.01 120.43 1.08

IC C5	C4	C13 C12	1.51	119.22	-0.05 118.64 1.51

IC C5	C6	C7	H2	1.51	119.69	0.15 119.76 1.08

IC C5	C6	C11 C12	1.51	120.53	-0.01 119.98 1.51

IC C12 C11	C10 O2	1.51	120.34	-0.13 121.49 1.40

IC C12 C13	C	O4	1.51	121.63	0.03 121.71 1.40

IC O2	C10	C9	H3	1.40	118.85	0.09 118.89 1.08

IC O3	C2	C1	H	1.40	121.43	-0.03 120.75 1.08

IC O3	C2	C3	H1	1.40	118.96	0.09 118.96 1.08

IC O4	C	C1	H	1.40	118.82	-0.03 118.55 1.08

IC C14 C8	C7	H2	1.53	119.73	-0.13 119.72 1.08

IC C14 C8	C9	H3	1.53	120.83	0.02 120.18 1.08

IC C1	C13 *C	O4	0.00	0.00 180.00	0.00 0.00

IC C2	C	*C1	H	0.00	0.00 180.00	0.00 0.00

IC C3	C1	*C2	O3	0.00	0.00 180.00	0.00 0.00

IC C4	C2	*C3	H1	0.00	0.00 180.00	0.00 0.00

IC C13 C3	*C4	C5	0.00	0.00 180.00	0.00 0.00

IC C6	C4	*C5	O	0.00	0.00 180.00	0.00 0.00

IC C11 C5	*C6	C7	0.00	0.00 180.00	0.00 0.00

IC C8	C6	*C7	H2	0.00	0.00 180.00	0.00 0.00

IC C9	C7	*C8	C14	0.00	0.00 180.00	0.00 0.00

IC C10 C8	*C9	H3	0.00	0.00 180.00	0.00 0.00

IC C10 C6	*C11 C12	0.00	0.00 180.00	0.00 0.00

IC C13 C11 *C12 O1	0.00	0.00 180.00	0.00 0.00

IC C11 C9	*C10 O2	0.00	0.00 180.00	0.00 0.00

IC H7	C8	*C14 H8	0.00	0.00 120.00	0.00 0.00

IC H7	C8	*C14 H9	0.00	0.00 -120.00	0.00 0.00

END
